# Supplementary material for: Genetic evolution of influenza viruses among selected countries in Latin America, 2017–2018
Source: PLoS One. 2020 Mar 10;15(3):e0227962. doi: 10.1371/journal.pone.0227962 (PMC7064222; doi:10.1371/journal.pone.0227962)
Supplement: S1 Table — (DOCX) [file pone.0227962.s001.docx]

**S1 Table. Sequences from participating countries available in GISAID included in the study.**

| *We acknowledge the authors, originating and submitting laboratories of the sequences from GISAID’s EpiFlu*™ Database on which this research is based. The list is detailed below. | | | | | | | | |  |
| --- | --- | --- | --- | --- | --- | --- | --- | --- | --- |
| *All submitters of data may be contacted directly via the GISAID website* ***www.gisaid.org*** | | | | | | | | |  |
| **EPI** | **Segment ID** | **Virus** | **Country** | **Collection date** | **Isolate name** | **Originating Lab** | **Submitting Lab** | **Authors** | |
|  |  |  |  |  |  |  |  |  | |
| EPI_ISL_281610 | EPI1282597 | A/H1pdm09 | Argentina | 2018-Jul-15 | A/Argentina/224/2018 | Instituto Nacional de Enfermedades Infecciosas | Centers for Disease Control and Prevention |  | |
| EPI_ISL_281611 | EPI1282587 | A/H1pdm09 | Argentina | 2018-Jul-19 | A/Argentina/275/2018 | Instituto Nacional de Enfermedades Infecciosas | Centers for Disease Control and Prevention |  | |
| EPI_ISL_281612 | EPI1282579 | A/H1pdm09 | Argentina | 2018-Jul-10 | A/Argentina/227/2018 | Instituto Nacional de Enfermedades Infecciosas | Centers for Disease Control and Prevention |  | |
| EPI_ISL_309491 | EPI1282426 | A/H1pdm09 | Argentina | 2018-Jul-19 | A/Argentina/222/2018 | Instituto Nacional de Enfermedades Infecciosas | Centers for Disease Control and Prevention |  | |
| EPI_ISL_319766 | EPI1282328 | A/H1pdm09 | Argentina | 2018-Jul-24 | A/Argentina/264/2018 | Instituto Nacional de Enfermedades Infecciosas | Centers for Disease Control and Prevention |  | |
| EPI_ISL_319767 | EPI1266738 | A/H1pdm09 | Argentina | 2018-May-18 | A/Argentina/34/2018 | Instituto Nacional de Enfermedades Infecciosas | Centers for Disease Control and Prevention |  | |
| EPI_ISL_319768 | EPI1266730 | A/H1pdm09 | Argentina | 2018-Jun-21 | A/Argentina/78/2018 | Instituto Nacional de Enfermedades Infecciosas | Centers for Disease Control and Prevention |  | |
| EPI_ISL_319769 | EPI1266722 | A/H1pdm09 | Argentina | 2018-Jun-08 | A/Argentina/79/2018 | Instituto Nacional de Enfermedades Infecciosas | Centers for Disease Control and Prevention |  | |
| EPI_ISL_319770 | EPI1266714 | A/H1pdm09 | Argentina | 2018-Jun-01 | A/Argentina/77/2018 | Instituto Nacional de Enfermedades Infecciosas | Centers for Disease Control and Prevention |  | |
| EPI_ISL_319771 | EPI1266706 | A/H1pdm09 | Argentina | 2018-May-26 | A/Argentina/39/2018 | Instituto Nacional de Enfermedades Infecciosas | Centers for Disease Control and Prevention |  | |
| EPI_ISL_319772 | EPI1266699 | A/H1pdm09 | Argentina | 2018-May-28 | A/Argentina/46/2018 | Instituto Nacional de Enfermedades Infecciosas | Centers for Disease Control and Prevention |  | |
| EPI_ISL_319773 | EPI1266691 | A/H1pdm09 | Argentina | 2018-Jun-11 | A/Argentina/57/2018 | Instituto Nacional de Enfermedades Infecciosas | Centers for Disease Control and Prevention |  | |
| EPI_ISL_319774 | EPI1266683 | A/H1pdm09 | Argentina | 2018-May-23 | A/Argentina/64/2018 | Instituto Nacional de Enfermedades Infecciosas | Centers for Disease Control and Prevention |  | |
| EPI_ISL_319775 | EPI1266675 | A/H1pdm09 | Argentina | 2018-Jun-07 | A/Argentina/65/2018 | Instituto Nacional de Enfermedades Infecciosas | Centers for Disease Control and Prevention |  | |
| EPI_ISL_319776 | EPI1266668 | A/H1pdm09 | Argentina | 2018-Jun-05 | A/Argentina/68/2018 | Instituto Nacional de Enfermedades Infecciosas | Centers for Disease Control and Prevention |  | |
| EPI_ISL_319777 | EPI1266661 | A/H1pdm09 | Argentina | 2018-Jun-06 | A/Argentina/73/2018 | Instituto Nacional de Enfermedades Infecciosas | Centers for Disease Control and Prevention |  | |
| EPI_ISL_322891 | EPI1266653 | A/H1pdm09 | Argentina | 2018-Jun-12 | A/Argentina/75/2018 | Instituto Nacional de Enfermedades Infecciosas | Centers for Disease Control and Prevention |  | |
| EPI_ISL_322904 | EPI1227652 | A/H1pdm09 | Argentina | 2018-Jan-29 | A/Argentina/10/2018 | Instituto Nacional de Enfermedades Infecciosas | Centers for Disease Control and Prevention |  | |
| EPI_ISL_322923 | EPI1314179 | A/H1pdm09 | Argentina | 2018-Jul-21 | A/Argentina/13762/2018 | CEMIC University Hospital | Centers for Disease Control and Prevention |  | |
| EPI_ISL_322924 | EPI1312740 | A/H1pdm09 | Argentina | 2018-Aug-15 | A/Argentina/13808/2018 | CEMIC University Hospital | Centers for Disease Control and Prevention |  | |
| EPI_ISL_322925 | EPI1312677 | A/H1pdm09 | Argentina | 2018-Jul-17 | A/Argentina/13764/2018 | CEMIC University Hospital | Centers for Disease Control and Prevention |  | |
| EPI_ISL_329866 | EPI1312606 | A/H1pdm09 | Argentina | 2018-Aug-15 | A/Argentina/13806/2018 | CEMIC University Hospital | Centers for Disease Control and Prevention |  | |
| EPI_ISL_329908 | EPI1312390 | A/H1pdm09 | Argentina | 2018-Aug-06 | A/Argentina/13782/2018 | CEMIC University Hospital | Centers for Disease Control and Prevention |  | |
| EPI_ISL_330164 | EPI1312359 | A/H1pdm09 | Argentina | 2018-Aug-13 | A/Argentina/13802/2018 | CEMIC University Hospital | Centers for Disease Control and Prevention |  | |
| EPI_ISL_330168 | EPI1311064 | A/H1pdm09 | Argentina | 2018-Aug-18 | A/Argentina/13824/2018 | CEMIC University Hospital | Centers for Disease Control and Prevention |  | |
| EPI_ISL_330195 | EPI1310737 | A/H1pdm09 | Argentina | 2018-Jul-31 | A/Argentina/13779/2018 | CEMIC University Hospital | Centers for Disease Control and Prevention |  | |
| EPI_ISL_330204 | EPI1078507 | A/H1pdm09 | Argentina | 2017-Jun-01 | A/Argentina/12780/2017 | CEMIC University Hospital | Centers for Disease Control and Prevention |  | |
| EPI_ISL_330212 | EPI1078499 | A/H1pdm09 | Argentina | 2017-May-21 | A/Argentina/12776/2017 | CEMIC University Hospital | Centers for Disease Control and Prevention |  | |
| EPI_ISL_330440 | EPI1078491 | A/H1pdm09 | Argentina | 2017-May-31 | A/Argentina/12777/2017 | CEMIC University Hospital | Centers for Disease Control and Prevention |  | |
| EPI_ISL_275743 | EPI1268536 | A/H3 | Argentina | 2018-Jun-07 | A/Argentina/56/2018 | Instituto Nacional de Enfermedades Infecciosas | Centers for Disease Control and Prevention |  | |
| EPI_ISL_275744 | EPI1268533 | A/H3 | Argentina | 2018-Jun-18 | A/Argentina/71/2018 | Instituto Nacional de Enfermedades Infecciosas | Centers for Disease Control and Prevention |  | |
| EPI_ISL_275745 | EPI1266388 | A/H3 | Argentina | 2018-May-17 | A/Argentina/37/2018 | Instituto Nacional de Enfermedades Infecciosas | Centers for Disease Control and Prevention |  | |
| EPI_ISL_275746 | EPI1266381 | A/H3 | Argentina | 2018-Jun-14 | A/Argentina/70/2018 | Instituto Nacional de Enfermedades Infecciosas | Centers for Disease Control and Prevention |  | |
| EPI_ISL_275747 | EPI1227969 | A/H3 | Argentina | 2018-Jan-29 | A/Argentina/11/2018 | Instituto Nacional de Enfermedades Infecciosas | Centers for Disease Control and Prevention |  | |
| EPI_ISL_275748 | EPI1047947 | A/H3 | Argentina | 2017-Jun-26 | A/Argentina/1810/2017 | Instituto Nacional de Enfermedades Infecciosas | Centers for Disease Control and Prevention |  | |
| EPI_ISL_275749 | EPI1047930 | A/H3 | Argentina | 2017-Jun-25 | A/Argentina/1566/2017 | Instituto Nacional de Enfermedades Infecciosas | Centers for Disease Control and Prevention |  | |
| EPI_ISL_275751 | EPI1047922 | A/H3 | Argentina | 2017-Jun-16 | A/Argentina/1334/2017 | Instituto Nacional de Enfermedades Infecciosas | Centers for Disease Control and Prevention |  | |
| EPI_ISL_281465 | EPI1047913 | A/H3 | Argentina | 2017-May-15 | A/Argentina/164/2017 | Instituto Nacional de Enfermedades Infecciosas | Centers for Disease Control and Prevention |  | |
| EPI_ISL_281466 | EPI1047904 | A/H3 | Argentina | 2017-Jun-16 | A/Argentina/1277/2017 | Instituto Nacional de Enfermedades Infecciosas | Centers for Disease Control and Prevention |  | |
| EPI_ISL_281467 | EPI1047896 | A/H3 | Argentina | 2017-Jun-13 | A/Argentina/984/2017 | Instituto Nacional de Enfermedades Infecciosas | Centers for Disease Control and Prevention |  | |
| EPI_ISL_281468 | EPI1047887 | A/H3 | Argentina | 2017-Jun-07 | A/Argentina/768/2017 | Instituto Nacional de Enfermedades Infecciosas | Centers for Disease Control and Prevention |  | |
| EPI_ISL_281469 | EPI1047879 | A/H3 | Argentina | 2017-Jun-06 | A/Argentina/892/2017 | Instituto Nacional de Enfermedades Infecciosas | Centers for Disease Control and Prevention |  | |
| EPI_ISL_281470 | EPI1079404 | A/H3 | Argentina | 2017-Jun-30 | A/Argentina/13060/2017 | CEMIC University Hospital | Centers for Disease Control and Prevention |  | |
| EPI_ISL_281471 | EPI1079396 | A/H3 | Argentina | 2017-Jun-29 | A/Argentina/13035/2017 | CEMIC University Hospital | Centers for Disease Control and Prevention |  | |
| EPI_ISL_281472 | EPI1077456 | A/H3 | Argentina | 2017-Jul-19 | A/Argentina/13323/2017 | CEMIC University Hospital | Centers for Disease Control and Prevention |  | |
| EPI_ISL_281473 | EPI1077448 | A/H3 | Argentina | 2017-Jul-24 | A/Argentina/13235/2017 | CEMIC University Hospital | Centers for Disease Control and Prevention |  | |
| EPI_ISL_281474 | EPI1077440 | A/H3 | Argentina | 2017-Jul-20 | A/Argentina/13198/2017 | CEMIC University Hospital | Centers for Disease Control and Prevention |  | |
| EPI_ISL_281475 | EPI1077432 | A/H3 | Argentina | 2017-Jul-18 | A/Argentina/13178/2017 | CEMIC University Hospital | Centers for Disease Control and Prevention |  | |
| EPI_ISL_281476 | EPI1077424 | A/H3 | Argentina | 2017-Jul-18 | A/Argentina/13176/2017 | CEMIC University Hospital | Centers for Disease Control and Prevention |  | |
| EPI_ISL_281477 | EPI1077416 | A/H3 | Argentina | 2017-Jul-18 | A/Argentina/13169/2017 | CEMIC University Hospital | Centers for Disease Control and Prevention |  | |
| EPI_ISL_281478 | EPI1077408 | A/H3 | Argentina | 2017-Jul-10 | A/Argentina/13127/2017 | CEMIC University Hospital | Centers for Disease Control and Prevention |  | |
| EPI_ISL_281479 | EPI1077400 | A/H3 | Argentina | 2017-Jul-10 | A/Argentina/13121/2017 | CEMIC University Hospital | Centers for Disease Control and Prevention |  | |
| EPI_ISL_281480 | EPI1077392 | A/H3 | Argentina | 2017-Jul-06 | A/Argentina/13076/2017 | CEMIC University Hospital | Centers for Disease Control and Prevention |  | |
| EPI_ISL_281722 | EPI1077384 | A/H3 | Argentina | 2017-Jun-30 | A/Argentina/13059/2017 | CEMIC University Hospital | Centers for Disease Control and Prevention |  | |
| EPI_ISL_281723 | EPI1077376 | A/H3 | Argentina | 2017-Jun-29 | A/Argentina/13034/2017 | CEMIC University Hospital | Centers for Disease Control and Prevention |  | |
| EPI_ISL_309531 | EPI1077368 | A/H3 | Argentina | 2017-Jun-29 | A/Argentina/13010/2017 | CEMIC University Hospital | Centers for Disease Control and Prevention |  | |
| EPI_ISL_319732 | EPI1077360 | A/H3 | Argentina | 2017-Jun-23 | A/Argentina/12924/2017 | CEMIC University Hospital | Centers for Disease Control and Prevention |  | |
| EPI_ISL_319733 | EPI1077352 | A/H3 | Argentina | 2017-Jun-21 | A/Argentina/12902/2017 | CEMIC University Hospital | Centers for Disease Control and Prevention |  | |
| EPI_ISL_320270 | EPI1077344 | A/H3 | Argentina | 2017-Jun-15 | A/Argentina/12878/2017 | CEMIC University Hospital | Centers for Disease Control and Prevention |  | |
| EPI_ISL_320271 | EPI1077336 | A/H3 | Argentina | 2017-May-15 | A/Argentina/12875/2017 | CEMIC University Hospital | Centers for Disease Control and Prevention |  | |
| EPI_ISL_281642 | EPI1078716 | B/Vic | Argentina | 2017-Jul-27 | B/Argentina/13339/2017 | CEMIC University Hospital | Centers for Disease Control and Prevention |  | |
| EPI_ISL_309641 | EPI1228640 | B/Vic | Argentina | 2017-Dec-27 | B/Argentina/8/2017 | Instituto Nacional de Enfermedades Infecciosas | Centers for Disease Control and Prevention |  | |
| EPI_ISL_309642 | EPI1228648 | B/Vic | Argentina | 2018-Jan-02 | B/Argentina/1/2018 | Instituto Nacional de Enfermedades Infecciosas | Centers for Disease Control and Prevention |  | |
| EPI_ISL_313120 | EPI1247017 | B/Vic | Argentina | 2017-Dec-21 | B/Argentina/7/2017 | Instituto Nacional de Enfermedades Infecciosas | Centers for Disease Control and Prevention |  | |
| EPI_ISL_313121 | EPI1247025 | B/Vic | Argentina | 2018-Jan-29 | B/Argentina/9/2018 | Instituto Nacional de Enfermedades Infecciosas | Centers for Disease Control and Prevention |  | |
| EPI_ISL_319674 | EPI1265924 | B/Vic | Argentina | 2018-Jun-08 | B/Argentina/58/2018 | Instituto Nacional de Enfermedades Infecciosas | Centers for Disease Control and Prevention |  | |
| EPI_ISL_319675 | EPI1265932 | B/Vic | Argentina | 2018-Jun-04 | B/Argentina/42/2018 | Instituto Nacional de Enfermedades Infecciosas | Centers for Disease Control and Prevention |  | |
| EPI_ISL_329930 | EPI1311235 | B/Vic | Argentina | 2018-Jul-06 | B/Argentina/13738/2018 | CEMIC University Hospital | Centers for Disease Control and Prevention |  | |
| EPI_ISL_281637 | EPI1281961 | B/Yam | Argentina | 2018-Jul-24 | B/Argentina/277/2018 | Instituto Nacional de Enfermedades Infecciosas | Centers for Disease Control and Prevention |  | |
| EPI_ISL_281638 | EPI1281945 | B/Yam | Argentina | 2018-Jul-20 | B/Argentina/232/2018 | Instituto Nacional de Enfermedades Infecciosas | Centers for Disease Control and Prevention |  | |
| EPI_ISL_281639 | EPI1281914 | B/Yam | Argentina | 2018-Jul-23 | B/Argentina/263/2018 | Instituto Nacional de Enfermedades Infecciosas | Centers for Disease Control and Prevention |  | |
| EPI_ISL_281640 | EPI1281910 | B/Yam | Argentina | 2018-Jul-10 | B/Argentina/206/2018 | Instituto Nacional de Enfermedades Infecciosas | Centers for Disease Control and Prevention |  | |
| EPI_ISL_281641 | EPI1281902 | B/Yam | Argentina | 2018-Jul-13 | B/Argentina/218/2018 | Instituto Nacional de Enfermedades Infecciosas | Centers for Disease Control and Prevention |  | |
| EPI_ISL_309657 | EPI1281894 | B/Yam | Argentina | 2018-Jul-30 | B/Argentina/360/2018 | Instituto Nacional de Enfermedades Infecciosas | Centers for Disease Control and Prevention |  | |
| EPI_ISL_319676 | EPI1281890 | B/Yam | Argentina | 2018-Jul-27 | B/Argentina/335/2018 | Instituto Nacional de Enfermedades Infecciosas | Centers for Disease Control and Prevention |  | |
| EPI_ISL_322840 | EPI1265940 | B/Yam | Argentina | 2018-May-31 | B/Argentina/40/2018 | Instituto Nacional de Enfermedades Infecciosas | Centers for Disease Control and Prevention |  | |
| EPI_ISL_322841 | EPI1228766 | B/Yam | Argentina | 2018-Feb-12 | B/Argentina/14/2018 | Instituto Nacional de Enfermedades Infecciosas | Centers for Disease Control and Prevention |  | |
| EPI_ISL_322842 | EPI1312972 | B/Yam | Argentina | 2018-Aug-14 | B/Argentina/13803/2018 | CEMIC University Hospital | Centers for Disease Control and Prevention |  | |
| EPI_ISL_322843 | EPI1312764 | B/Yam | Argentina | 2018-Jul-23 | B/Argentina/13769/2018 | CEMIC University Hospital | Centers for Disease Control and Prevention |  | |
| EPI_ISL_322844 | EPI1312756 | B/Yam | Argentina | 2018-Jul-25 | B/Argentina/13771/2018 | CEMIC University Hospital | Centers for Disease Control and Prevention |  | |
| EPI_ISL_322848 | EPI1312748 | B/Yam | Argentina | 2018-Jul-26 | B/Argentina/13763/2018 | CEMIC University Hospital | Centers for Disease Control and Prevention |  | |
| EPI_ISL_322850 | EPI1311267 | B/Yam | Argentina | 2018-Aug-14 | B/Argentina/13804/2018 | CEMIC University Hospital | Centers for Disease Control and Prevention |  | |
| EPI_ISL_329932 | EPI1311251 | B/Yam | Argentina | 2018-Aug-06 | B/Argentina/13785/2018 | CEMIC University Hospital | Centers for Disease Control and Prevention |  | |
| EPI_ISL_329934 | EPI1078709 | B/Yam | Argentina | 2017-Jul-27 | B/Argentina/13252/2017 | CEMIC University Hospital | Centers for Disease Control and Prevention |  | |
| EPI_ISL_330213 | EPI1078701 | B/Yam | Argentina | 2017-Jul-27 | B/Argentina/13248/2017 | CEMIC University Hospital | Centers for Disease Control and Prevention |  | |
| EPI_ISL_330214 | EPI1078693 | B/Yam | Argentina | 2017-Jul-25 | B/Argentina/13240/2017 | CEMIC University Hospital | Centers for Disease Control and Prevention |  | |
| EPI_ISL_330215 | EPI1078685 | B/Yam | Argentina | 2017-Jul-17 | B/Argentina/13162/2017 | CEMIC University Hospital | Centers for Disease Control and Prevention |  | |
| EPI_ISL_330241 | EPI1078677 | B/Yam | Argentina | 2017-Jun-30 | B/Argentina/13047/2017 | CEMIC University Hospital | Centers for Disease Control and Prevention |  | |
| EPI_ISL_269785 | EPI1254840 | A/H1pdm09 | Brazil | 2018-Feb-22 | A/Pernambuco/151633-IEC/2018 | Evandro Chagas Institute | Evandro Chagas Institute |  | |
| EPI_ISL_273296 | EPI1254832 | A/H1pdm09 | Brazil | 2018-Mar-05 | A/Paraiba/151520-IEC/2018 | Evandro Chagas Institute | Evandro Chagas Institute |  | |
| EPI_ISL_273297 | EPI1254830 | A/H1pdm09 | Brazil | 2018-Mar-27 | A/Para/151778-IEC/2018 | Evandro Chagas Institute | Evandro Chagas Institute |  | |
| EPI_ISL_281608 | EPI1238756 | A/H1pdm09 | Brazil | 2018-Feb-27 | A/Mato Grosso do Sul/511644/2018 | Instituto Adolfo Lutz | Instituto Adolfo Lutz | Santos,Katia;Silva,Daniela;Benega,Margarete;Santos,Cecilia;Paiva,Terezinha | |
| EPI_ISL_299593 | EPI1238755 | A/H1pdm09 | Brazil | 2018-Mar-14 | A/Goias/891438/2018 | Instituto Adolfo Lutz | Instituto Adolfo Lutz | Santos,Katia;Silva,Daniela;Benega,Margarete;Santos,Cecilia;Paiva,Terezinha | |
| EPI_ISL_306023 | EPI1238754 | A/H1pdm09 | Brazil | 2018-Mar-20 | A/Distrito Federal/754958/2018 | Instituto Adolfo Lutz | Instituto Adolfo Lutz | Santos,Katia;Silva,Daniela;Benega,Margarete;Santos,Cecilia;Paiva,Terezinha | |
| EPI_ISL_306028 | EPI1238752 | A/H1pdm09 | Brazil | 2018-Feb-14 | A/Goias/870796/2018 | Instituto Adolfo Lutz | Instituto Adolfo Lutz | Santos,Katia;Silva,Daniela;Benega,Margarete;Santos,Cecilia;Paiva,Terezinha | |
| EPI_ISL_306034 | EPI1179310 | A/H1pdm09 | Brazil | 2018-Jan-17 | A/Goias/857676/2018 | Instituto Adolfo Lutz | Instituto Adolfo Lutz | Santos,Katia;Silva,Daniela;Benega,Margarete;Paulino,Renato;Santos,Cecilia;Paiva,Terezinha | |
| EPI_ISL_306038 | EPI1036439 | A/H1pdm09 | Brazil | 2017-Jun-26 | A/Sao Paulo/545026/2017 | Instituto Adolfo Lutz | Instituto Adolfo Lutz | Santos,Katia;Silva,Daniela;Benega,Margarete;Santos,Cecilia;Paiva,Terezinha | |
| EPI_ISL_311499 | EPI1036438 | A/H1pdm09 | Brazil | 2017-Jun-19 | A/Sao Paulo/541135/2017 | Instituto Adolfo Lutz | Instituto Adolfo Lutz | Santos,Katia;Silva,Daniela;Benega,Margarete;Santos,Cecilia;Paiva,Terezinha | |
| EPI_ISL_311501 | EPI1023901 | A/H1pdm09 | Brazil | 2017-Jun-06 | A/Sao Paulo/537034/2017 | Instituto Adolfo Lutz | Instituto Adolfo Lutz | Santos,Katia;Silva,Daniela;Benega,Margarete;Santos,Cecilia;Paiva,Terezinha | |
| EPI_ISL_311503 | EPI1274443 | A/H1pdm09 | Brazil | 2018-May-02 | A/Bahia/561/2018 | Instituto Oswaldo Cruz FIOCRUZ - Laboratory of Respiratory Viruses and Measles (LVRS) | Centers for Disease Control and Prevention |  | |
| EPI_ISL_313567 | EPI1274435 | A/H1pdm09 | Brazil | 2018-May-15 | A/Bahia/564/2018 | Instituto Oswaldo Cruz FIOCRUZ - Laboratory of Respiratory Viruses and Measles (LVRS) | Centers for Disease Control and Prevention |  | |
| EPI_ISL_313568 | EPI1274427 | A/H1pdm09 | Brazil | 2018-Jun-06 | A/Minas Gerias/716/2018 | Instituto Oswaldo Cruz FIOCRUZ - Laboratory of Respiratory Viruses and Measles (LVRS) | Centers for Disease Control and Prevention |  | |
| EPI_ISL_313569 | EPI1249322 | A/H1pdm09 | Brazil | 2018-Mar-11 | A/Bahia/122/2018 | Instituto Oswaldo Cruz FIOCRUZ - Laboratory of Respiratory Viruses and Measles (LVRS) | Centers for Disease Control and Prevention |  | |
| EPI_ISL_313570 | EPI1249314 | A/H1pdm09 | Brazil | 2018-Apr-12 | A/Rio De Janeiro/233/2018 | Instituto Oswaldo Cruz FIOCRUZ - Laboratory of Respiratory Viruses and Measles (LVRS) | Centers for Disease Control and Prevention |  | |
| EPI_ISL_313571 | EPI1249306 | A/H1pdm09 | Brazil | 2018-Apr-21 | A/Sergipe/279/2018 | Instituto Oswaldo Cruz FIOCRUZ - Laboratory of Respiratory Viruses and Measles (LVRS) | Centers for Disease Control and Prevention |  | |
| EPI_ISL_313572 | EPI1249299 | A/H1pdm09 | Brazil | 2018-Apr-17 | A/Santa Catarina/258/2018 | Instituto Oswaldo Cruz FIOCRUZ - Laboratory of Respiratory Viruses and Measles (LVRS) | Centers for Disease Control and Prevention |  | |
| EPI_ISL_313573 | EPI1249291 | A/H1pdm09 | Brazil | 2018-Mar-23 | A/Alagoas/245/2018 | Instituto Oswaldo Cruz FIOCRUZ - Laboratory of Respiratory Viruses and Measles (LVRS) | Centers for Disease Control and Prevention |  | |
| EPI_ISL_313574 | EPI1249283 | A/H1pdm09 | Brazil | 2018-Mar-06 | A/Bahia/117/2018 | Instituto Oswaldo Cruz FIOCRUZ - Laboratory of Respiratory Viruses and Measles (LVRS) | Centers for Disease Control and Prevention |  | |
| EPI_ISL_313575 | EPI1249275 | A/H1pdm09 | Brazil | 2018-Apr-03 | A/Sergipe/221/2018 | Instituto Oswaldo Cruz FIOCRUZ - Laboratory of Respiratory Viruses and Measles (LVRS) | Centers for Disease Control and Prevention |  | |
| EPI_ISL_313576 | EPI1249267 | A/H1pdm09 | Brazil | 2018-Apr-11 | A/Sergipe/215/2018 | Instituto Oswaldo Cruz FIOCRUZ - Laboratory of Respiratory Viruses and Measles (LVRS) | Centers for Disease Control and Prevention |  | |
| EPI_ISL_313577 | EPI1249259 | A/H1pdm09 | Brazil | 2018-Apr-03 | A/Sergipe/214/2018 | Instituto Oswaldo Cruz FIOCRUZ - Laboratory of Respiratory Viruses and Measles (LVRS) | Centers for Disease Control and Prevention |  | |
| EPI_ISL_314557 | EPI1249251 | A/H1pdm09 | Brazil | 2018-Mar-10 | A/Parana/155/2018 | Instituto Oswaldo Cruz FIOCRUZ - Laboratory of Respiratory Viruses and Measles (LVRS) | Centers for Disease Control and Prevention |  | |
| EPI_ISL_314558 | EPI1249243 | A/H1pdm09 | Brazil | 2018-Mar-07 | A/Parana/154/2018 | Instituto Oswaldo Cruz FIOCRUZ - Laboratory of Respiratory Viruses and Measles (LVRS) | Centers for Disease Control and Prevention |  | |
| EPI_ISL_314562 | EPI1268245 | A/H1pdm09 | Brazil | 2018-May-03 | A/Acre/152563-IEC/2018 | National Influenza Center | Centers for Disease Control and Prevention |  | |
| EPI_ISL_316451 | EPI1268237 | A/H1pdm09 | Brazil | 2018-Apr-06 | A/Ceara/152545-IEC/2018 | National Influenza Center | Centers for Disease Control and Prevention |  | |
| EPI_ISL_316735 | EPI1268229 | A/H1pdm09 | Brazil | 2018-May-02 | A/Paraiba/152402-IEC/2018 | National Influenza Center | Centers for Disease Control and Prevention |  | |
| EPI_ISL_316736 | EPI1268221 | A/H1pdm09 | Brazil | 2018-Apr-21 | A/Acre/152338-IEC/2018 | National Influenza Center | Centers for Disease Control and Prevention |  | |
| EPI_ISL_316737 | EPI1268213 | A/H1pdm09 | Brazil | 2018-Apr-17 | A/Paraiba/152186-IEC/2018 | National Influenza Center | Centers for Disease Control and Prevention |  | |
| EPI_ISL_316738 | EPI1268205 | A/H1pdm09 | Brazil | 2018-Apr-10 | A/Acre/152155-IEC/2018 | National Influenza Center | Centers for Disease Control and Prevention |  | |
| EPI_ISL_316740 | EPI1268197 | A/H1pdm09 | Brazil | 2018-Apr-23 | A/Maranhao/152146-IEC/2018 | National Influenza Center | Centers for Disease Control and Prevention |  | |
| EPI_ISL_316741 | EPI1268189 | A/H1pdm09 | Brazil | 2018-May-30 | A/Paraiba/152911-IEC/2018 | National Influenza Center | Centers for Disease Control and Prevention |  | |
| EPI_ISL_316744 | EPI1268181 | A/H1pdm09 | Brazil | 2018-Apr-16 | A/Ceara/153529-IEC/2018 | National Influenza Center | Centers for Disease Control and Prevention |  | |
| EPI_ISL_316745 | EPI1268173 | A/H1pdm09 | Brazil | 2018-Apr-18 | A/Ceara/153525-IEC/2018 | National Influenza Center | Centers for Disease Control and Prevention |  | |
| EPI_ISL_316746 | EPI1268165 | A/H1pdm09 | Brazil | 2018-Apr-26 | A/Ceara/153518-IEC/2018 | National Influenza Center | Centers for Disease Control and Prevention |  | |
| EPI_ISL_316748 | EPI1268157 | A/H1pdm09 | Brazil | 2018-Apr-20 | A/Ceara/153517-IEC/2018 | National Influenza Center | Centers for Disease Control and Prevention |  | |
| EPI_ISL_316749 | EPI1268149 | A/H1pdm09 | Brazil | 2018-Apr-23 | A/Ceara/153516-IEC/2018 | National Influenza Center | Centers for Disease Control and Prevention |  | |
| EPI_ISL_319841 | EPI1268142 | A/H1pdm09 | Brazil | 2018-Apr-19 | A/Ceara/153515-IEC/2018 | National Influenza Center | Centers for Disease Control and Prevention |  | |
| EPI_ISL_319842 | EPI1268134 | A/H1pdm09 | Brazil | 2018-Apr-20 | A/Ceara/153514-IEC/2018 | National Influenza Center | Centers for Disease Control and Prevention |  | |
| EPI_ISL_319843 | EPI1268126 | A/H1pdm09 | Brazil | 2018-Apr-16 | A/Para/153282-IEC/2018 | National Influenza Center | Centers for Disease Control and Prevention |  | |
| EPI_ISL_319844 | EPI1268118 | A/H1pdm09 | Brazil | 2018-May-23 | A/Amazonas/153165-IEC/2018 | National Influenza Center | Centers for Disease Control and Prevention |  | |
| EPI_ISL_320200 | EPI1268110 | A/H1pdm09 | Brazil | 2018-May-15 | A/Acre/152954-IEC/2018 | National Influenza Center | Centers for Disease Control and Prevention |  | |
| EPI_ISL_320216 | EPI1271096 | A/H1pdm09 | Brazil | 2018-May-25 | A/Brazil/3713/2018 | Instituto Adolfo Lutz | Centers for Disease Control and Prevention |  | |
| EPI_ISL_320217 | EPI1267983 | A/H1pdm09 | Brazil | 2018-Jun-11 | A/Brazil/6359/2018 | Instituto Adolfo Lutz | Centers for Disease Control and Prevention |  | |
| EPI_ISL_320218 | EPI1267268 | A/H1pdm09 | Brazil | 2018-Apr-02 | A/Brazil/1469/2018 | Instituto Adolfo Lutz | Centers for Disease Control and Prevention |  | |
| EPI_ISL_320219 | EPI1267260 | A/H1pdm09 | Brazil | 2018-May-16 | A/Brazil/6406/2018 | Instituto Adolfo Lutz | Centers for Disease Control and Prevention |  | |
| EPI_ISL_320220 | EPI1267252 | A/H1pdm09 | Brazil | 2018-Jun-06 | A/Brazil/9363/2018 | Instituto Adolfo Lutz | Centers for Disease Control and Prevention |  | |
| EPI_ISL_320221 | EPI1267244 | A/H1pdm09 | Brazil | 2018-Jun-15 | A/Brazil/2817/2018 | Instituto Adolfo Lutz | Centers for Disease Control and Prevention |  | |
| EPI_ISL_320222 | EPI1262402 | A/H1pdm09 | Brazil | 2018-Apr-27 | A/Brazil/3169/2018 | Instituto Adolfo Lutz | Centers for Disease Control and Prevention |  | |
| EPI_ISL_320223 | EPI1262394 | A/H1pdm09 | Brazil | 2018-May-07 | A/Brazil/0344/2018 | Instituto Adolfo Lutz | Centers for Disease Control and Prevention |  | |
| EPI_ISL_320224 | EPI1262378 | A/H1pdm09 | Brazil | 2018-Mar-14 | A/Brazil/1438/2018 | Instituto Adolfo Lutz | Centers for Disease Control and Prevention |  | |
| EPI_ISL_320225 | EPI1262370 | A/H1pdm09 | Brazil | 2018-May-05 | A/Brazil/4428/2018 | Instituto Adolfo Lutz | Centers for Disease Control and Prevention |  | |
| EPI_ISL_320226 | EPI1262362 | A/H1pdm09 | Brazil | 2018-Feb-27 | A/Brazil/1644/2018 | Instituto Adolfo Lutz | Centers for Disease Control and Prevention |  | |
| EPI_ISL_320227 | EPI1262338 | A/H1pdm09 | Brazil | 2018-Jun-08 | A/Brazil/1524/2018 | Instituto Adolfo Lutz | Centers for Disease Control and Prevention |  | |
| EPI_ISL_320228 | EPI1262330 | A/H1pdm09 | Brazil | 2018-Jun-06 | A/Brazil/0561/2018 | Instituto Adolfo Lutz | Centers for Disease Control and Prevention |  | |
| EPI_ISL_320229 | EPI1262314 | A/H1pdm09 | Brazil | 2018-Jun-08 | A/Brazil/0280/2018 | Instituto Adolfo Lutz | Centers for Disease Control and Prevention |  | |
| EPI_ISL_320230 | EPI1262306 | A/H1pdm09 | Brazil | 2018-Apr-03 | A/Brazil/2135/2018 | Instituto Adolfo Lutz | Centers for Disease Control and Prevention |  | |
| EPI_ISL_320231 | EPI1262298 | A/H1pdm09 | Brazil | 2018-May-05 | A/Brazil/112/2018 | Instituto Adolfo Lutz | Centers for Disease Control and Prevention |  | |
| EPI_ISL_320232 | EPI1262290 | A/H1pdm09 | Brazil | 2018-May-05 | A/Brazil/6326/2018 | Instituto Adolfo Lutz | Centers for Disease Control and Prevention |  | |
| EPI_ISL_320233 | EPI1261035 | A/H1pdm09 | Brazil | 2018-Jun-04 | A/Brazil/9331/2018 | Instituto Adolfo Lutz | Centers for Disease Control and Prevention |  | |
| EPI_ISL_320694 | EPI1078475 | A/H1pdm09 | Brazil | 2017-Jun-26 | A/Brazil/5026/2017 | Instituto Adolfo Lutz | Centers for Disease Control and Prevention |  | |
| EPI_ISL_321292 | EPI1211175 | A/H1pdm09 | Brazil | 2018-Feb-06 | A/Rio de Janeiro/46/2018 | Laboratório Central de Saúde Pública (LACEN RJ) | Instituto Oswaldo Cruz FIOCRUZ - Laboratory of Respiratory Viruses and Measles (LVRS) | NIC,FIOCRUZ | |
| EPI_ISL_321293 | EPI1211180 | A/H1pdm09 | Brazil | 2018-Feb-21 | A/Santa Catarina/82/2018 | LACEN SC | Instituto Oswaldo Cruz FIOCRUZ - Laboratory of Respiratory Viruses and Measles (LVRS) | NIC,FIOCRUZ | |
| EPI_ISL_321294 | EPI1211168 | A/H1pdm09 | Brazil | 2018-Jan-16 | A/Parana/53/2018 | Laboratório Central do Estado do Paraná - LACEN/PR | Instituto Oswaldo Cruz FIOCRUZ - Laboratory of Respiratory Viruses and Measles (LVRS) | NIC,FIOCRUZ | |
| EPI_ISL_321295 | EPI1211163 | A/H1pdm09 | Brazil | 2018-Jan-26 | A/Espirito Santo/86/2018 | LACEN/ES - Laboratório Central de Saúde Pública do Espírito Santo | Instituto Oswaldo Cruz FIOCRUZ - Laboratory of Respiratory Viruses and Measles (LVRS) | NIC,FIOCRUZ | |
| EPI_ISL_321296 | EPI1304991 | A/H1pdm09 | Brazil | 2018-Apr-10 | A/Ceara/152542-IEC/2018 | Evandro Chagas Institute | Evandro Chagas Institute |  | |
| EPI_ISL_321297 | EPI1304989 | A/H1pdm09 | Brazil | 2018-Apr-27 | A/Paraiba/152232-IEC/2018 | Evandro Chagas Institute | Evandro Chagas Institute |  | |
| EPI_ISL_321298 | EPI1304987 | A/H1pdm09 | Brazil | 2018-Apr-20 | A/Acre/152165-IEC/2018 | Evandro Chagas Institute | Evandro Chagas Institute |  | |
| EPI_ISL_321301 | EPI1274510 | A/H1pdm09 | Brazil | 2018-May-20 | A/Espirito Santo/739/2018 | Instituto Oswaldo Cruz FIOCRUZ - Laboratory of Respiratory Viruses and Measles (LVRS) | Centers for Disease Control and Prevention |  | |
| EPI_ISL_321302 | EPI1274502 | A/H1pdm09 | Brazil | 2018-Jun-01 | A/Parana/763/2018 | Instituto Oswaldo Cruz FIOCRUZ - Laboratory of Respiratory Viruses and Measles (LVRS) | Centers for Disease Control and Prevention |  | |
| EPI_ISL_321303 | EPI1274494 | A/H1pdm09 | Brazil | 2018-Jun-13 | A/Parana/768/2018 | Instituto Oswaldo Cruz FIOCRUZ - Laboratory of Respiratory Viruses and Measles (LVRS) | Centers for Disease Control and Prevention |  | |
| EPI_ISL_321956 | EPI1274486 | A/H1pdm09 | Brazil | 2018-Jun-15 | A/Parana/772/2018 | Instituto Oswaldo Cruz FIOCRUZ - Laboratory of Respiratory Viruses and Measles (LVRS) | Centers for Disease Control and Prevention |  | |
| EPI_ISL_321957 | EPI1274478 | A/H1pdm09 | Brazil | 2018-Jun-30 | A/Rio De Janeiro/821/2018 | Instituto Oswaldo Cruz FIOCRUZ - Laboratory of Respiratory Viruses and Measles (LVRS) | Centers for Disease Control and Prevention |  | |
| EPI_ISL_321963 | EPI1274470 | A/H1pdm09 | Brazil | 2018-Jul-11 | A/Rio De Janeiro/848/2018 | Instituto Oswaldo Cruz FIOCRUZ - Laboratory of Respiratory Viruses and Measles (LVRS) | Centers for Disease Control and Prevention |  | |
| EPI_ISL_322198 | EPI1274464 | A/H1pdm09 | Brazil | 2018-Jul-11 | A/Santa Catarina/865/2018 | Instituto Oswaldo Cruz FIOCRUZ - Laboratory of Respiratory Viruses and Measles (LVRS) | Centers for Disease Control and Prevention |  | |
| EPI_ISL_322199 | EPI1274456 | A/H1pdm09 | Brazil | 2018-May-07 | A/Rio Grande Du Sul/503/2018 | Instituto Oswaldo Cruz FIOCRUZ - Laboratory of Respiratory Viruses and Measles (LVRS) | Centers for Disease Control and Prevention |  | |
| EPI_ISL_322200 | EPI1310969 | A/H1pdm09 | Brazil | 2018-Jun-26 | A/Brazil/9204/2018 | Instituto Adolfo Lutz | Centers for Disease Control and Prevention |  | |
| EPI_ISL_322201 | EPI1310961 | A/H1pdm09 | Brazil | 2018-Jun-20 | A/Brazil/4335/2018 | Instituto Adolfo Lutz | Centers for Disease Control and Prevention |  | |
| EPI_ISL_322202 | EPI1310940 | A/H1pdm09 | Brazil | 2018-Jun-14 | A/Brazil/6002/2018 | Instituto Adolfo Lutz | Centers for Disease Control and Prevention |  | |
| EPI_ISL_322203 | EPI1310916 | A/H1pdm09 | Brazil | 2018-Jul-13 | A/Brazil/2699/2018 | Instituto Adolfo Lutz | Centers for Disease Control and Prevention |  | |
| EPI_ISL_322211 | EPI1310908 | A/H1pdm09 | Brazil | 2018-Jul-15 | A/Brazil/4519/2018 | Instituto Adolfo Lutz | Centers for Disease Control and Prevention |  | |
| EPI_ISL_322212 | EPI1310893 | A/H1pdm09 | Brazil | 2018-Jun-25 | A/Brazil/1031/2018 | Instituto Adolfo Lutz | Centers for Disease Control and Prevention |  | |
| EPI_ISL_322213 | EPI1282631 | A/H1pdm09 | Brazil | 2018-Jun-18 | A/Brazil/1680/2018 | Instituto Adolfo Lutz | Centers for Disease Control and Prevention |  | |
| EPI_ISL_322225 | EPI1282482 | A/H1pdm09 | Brazil | 2018-Jun-22 | A/Brazil/4395/2018 | Instituto Adolfo Lutz | Centers for Disease Control and Prevention |  | |
| EPI_ISL_322226 | EPI1277961 | A/H1pdm09 | Brazil | 2018-Jun-19 | A/Brazil/2299/2018 | Instituto Adolfo Lutz | Centers for Disease Control and Prevention |  | |
| EPI_ISL_322227 | EPI1277917 | A/H1pdm09 | Brazil | 2018-Jun-19 | A/Brazil/2391/2018 | Instituto Adolfo Lutz | Centers for Disease Control and Prevention |  | |
| EPI_ISL_322246 | EPI1277909 | A/H1pdm09 | Brazil | 2018-Jun-20 | A/Brazil/2313/2018 | Instituto Adolfo Lutz | Centers for Disease Control and Prevention |  | |
| EPI_ISL_322247 | EPI1297291 | A/H1pdm09 | Brazil | 2018-Jun-05 | A/Minas Gerais/707/2018 | FUNED MG | Instituto Oswaldo Cruz FIOCRUZ - Laboratory of Respiratory Viruses and Measles (LVRS) | NIC,FIOCRUZ | |
| EPI_ISL_322248 | EPI1279460 | A/H1pdm09 | Brazil | 2018-May-05 | A/Minas Gerais/583/2018 | FUNED MG | Instituto Oswaldo Cruz FIOCRUZ - Laboratory of Respiratory Viruses and Measles (LVRS) | NIC,FIOCRUZ | |
| EPI_ISL_322249 | EPI1279459 | A/H1pdm09 | Brazil | 2018-Apr-20 | A/Minas Gerais/572/2018 | FUNED MG | Instituto Oswaldo Cruz FIOCRUZ - Laboratory of Respiratory Viruses and Measles (LVRS) | NIC,FIOCRUZ | |
| EPI_ISL_322250 | EPI1279458 | A/H1pdm09 | Brazil | 2018-Mar-12 | A/Minas Gerais/136/2018 | FUNED MG | Instituto Oswaldo Cruz FIOCRUZ - Laboratory of Respiratory Viruses and Measles (LVRS) | NIC,FIOCRUZ | |
| EPI_ISL_322251 | EPI1297289 | A/H1pdm09 | Brazil | 2018-Jun-26 | A/Rio de Janeiro/646/2018 | Laboratório Central de Saúde Pública (LACEN RJ) | Instituto Oswaldo Cruz FIOCRUZ - Laboratory of Respiratory Viruses and Measles (LVRS) | NIC,FIOCRUZ | |
| EPI_ISL_322252 | EPI1297293 | A/H1pdm09 | Brazil | 2018-Jun-01 | A/Santa Catarina/593/2018 | LACEN SC | Instituto Oswaldo Cruz FIOCRUZ - Laboratory of Respiratory Viruses and Measles (LVRS) | NIC,FIOCRUZ | |
| EPI_ISL_322253 | EPI1297290 | A/H1pdm09 | Brazil | 2018-May-26 | A/Santa Catarina/489/2018 | LACEN SC | Instituto Oswaldo Cruz FIOCRUZ - Laboratory of Respiratory Viruses and Measles (LVRS) | NIC,FIOCRUZ | |
| EPI_ISL_322254 | EPI1279522 | A/H1pdm09 | Brazil | 2018-May-18 | A/Santa Catarina/485/2018 | LACEN SC | Instituto Oswaldo Cruz FIOCRUZ - Laboratory of Respiratory Viruses and Measles (LVRS) | NIC,FIOCRUZ | |
| EPI_ISL_322255 | EPI1279521 | A/H1pdm09 | Brazil | 2018-Apr-17 | A/Santa Catarina/259/2018 | LACEN SC | Instituto Oswaldo Cruz FIOCRUZ - Laboratory of Respiratory Viruses and Measles (LVRS) | NIC,FIOCRUZ | |
| EPI_ISL_322256 | EPI1279520 | A/H1pdm09 | Brazil | 2018-Mar-29 | A/Santa Catarina/211/2018 | LACEN SC | Instituto Oswaldo Cruz FIOCRUZ - Laboratory of Respiratory Viruses and Measles (LVRS) | NIC,FIOCRUZ | |
| EPI_ISL_322257 | EPI1279519 | A/H1pdm09 | Brazil | 2018-Mar-21 | A/Santa Catarina/160/2018 | LACEN SC | Instituto Oswaldo Cruz FIOCRUZ - Laboratory of Respiratory Viruses and Measles (LVRS) | NIC,FIOCRUZ | |
| EPI_ISL_322279 | EPI1297298 | A/H1pdm09 | Brazil | 2018-Jun-13 | A/Parana/769/2018 | Laboratório Central do Estado do Paraná - LACEN/PR | Instituto Oswaldo Cruz FIOCRUZ - Laboratory of Respiratory Viruses and Measles (LVRS) | NIC,FIOCRUZ | |
| EPI_ISL_322280 | EPI1297297 | A/H1pdm09 | Brazil | 2018-Jun-11 | A/Parana/767/2018 | Laboratório Central do Estado do Paraná - LACEN/PR | Instituto Oswaldo Cruz FIOCRUZ - Laboratory of Respiratory Viruses and Measles (LVRS) | NIC,FIOCRUZ | |
| EPI_ISL_322285 | EPI1297296 | A/H1pdm09 | Brazil | 2018-Jun-06 | A/Parana/766/2018 | Laboratório Central do Estado do Paraná - LACEN/PR | Instituto Oswaldo Cruz FIOCRUZ - Laboratory of Respiratory Viruses and Measles (LVRS) | NIC,FIOCRUZ | |
| EPI_ISL_322286 | EPI1297295 | A/H1pdm09 | Brazil | 2018-Jun-05 | A/Parana/765/2018 | Laboratório Central do Estado do Paraná - LACEN/PR | Instituto Oswaldo Cruz FIOCRUZ - Laboratory of Respiratory Viruses and Measles (LVRS) | NIC,FIOCRUZ | |
| EPI_ISL_322287 | EPI1297294 | A/H1pdm09 | Brazil | 2018-Jun-08 | A/Parana/764/2018 | Laboratório Central do Estado do Paraná - LACEN/PR | Instituto Oswaldo Cruz FIOCRUZ - Laboratory of Respiratory Viruses and Measles (LVRS) | NIC,FIOCRUZ | |
| EPI_ISL_322288 | EPI1279490 | A/H1pdm09 | Brazil | 2018-Apr-28 | A/Parana/470/2018 | Laboratório Central do Estado do Paraná - LACEN/PR | Instituto Oswaldo Cruz FIOCRUZ - Laboratory of Respiratory Viruses and Measles (LVRS) | NIC,FIOCRUZ | |
| EPI_ISL_322302 | EPI1279489 | A/H1pdm09 | Brazil | 2018-May-02 | A/Parana/462/2018 | Laboratório Central do Estado do Paraná - LACEN/PR | Instituto Oswaldo Cruz FIOCRUZ - Laboratory of Respiratory Viruses and Measles (LVRS) | NIC,FIOCRUZ | |
| EPI_ISL_322303 | EPI1279488 | A/H1pdm09 | Brazil | 2018-Apr-25 | A/Parana/319/2018 | Laboratório Central do Estado do Paraná - LACEN/PR | Instituto Oswaldo Cruz FIOCRUZ - Laboratory of Respiratory Viruses and Measles (LVRS) | NIC,FIOCRUZ | |
| EPI_ISL_322304 | EPI1279487 | A/H1pdm09 | Brazil | 2018-Apr-16 | A/Parana/315/2018 | Laboratório Central do Estado do Paraná - LACEN/PR | Instituto Oswaldo Cruz FIOCRUZ - Laboratory of Respiratory Viruses and Measles (LVRS) | NIC,FIOCRUZ | |
| EPI_ISL_322911 | EPI1279486 | A/H1pdm09 | Brazil | 2018-Mar-23 | A/Parana/149/2018 | Laboratório Central do Estado do Paraná - LACEN/PR | Instituto Oswaldo Cruz FIOCRUZ - Laboratory of Respiratory Viruses and Measles (LVRS) | NIC,FIOCRUZ | |
| EPI_ISL_322929 | EPI1279485 | A/H1pdm09 | Brazil | 2018-Mar-21 | A/Parana/148/2018 | Laboratório Central do Estado do Paraná - LACEN/PR | Instituto Oswaldo Cruz FIOCRUZ - Laboratory of Respiratory Viruses and Measles (LVRS) | NIC,FIOCRUZ | |
| EPI_ISL_326788 | EPI1279484 | A/H1pdm09 | Brazil | 2018-Mar-21 | A/Parana/147/2018 | Laboratório Central do Estado do Paraná - LACEN/PR | Instituto Oswaldo Cruz FIOCRUZ - Laboratory of Respiratory Viruses and Measles (LVRS) | NIC,FIOCRUZ | |
| EPI_ISL_326789 | EPI1279483 | A/H1pdm09 | Brazil | 2018-Mar-22 | A/Parana/146/2018 | Laboratório Central do Estado do Paraná - LACEN/PR | Instituto Oswaldo Cruz FIOCRUZ - Laboratory of Respiratory Viruses and Measles (LVRS) | NIC,FIOCRUZ | |
| EPI_ISL_326790 | EPI1279482 | A/H1pdm09 | Brazil | 2018-Mar-21 | A/Parana/145/2018 | Laboratório Central do Estado do Paraná - LACEN/PR | Instituto Oswaldo Cruz FIOCRUZ - Laboratory of Respiratory Viruses and Measles (LVRS) | NIC,FIOCRUZ | |
| EPI_ISL_326791 | EPI1279481 | A/H1pdm09 | Brazil | 2018-Mar-20 | A/Parana/144/2018 | Laboratório Central do Estado do Paraná - LACEN/PR | Instituto Oswaldo Cruz FIOCRUZ - Laboratory of Respiratory Viruses and Measles (LVRS) | NIC,FIOCRUZ | |
| EPI_ISL_326792 | EPI1279480 | A/H1pdm09 | Brazil | 2018-Mar-12 | A/Parana/143/2018 | Laboratório Central do Estado do Paraná - LACEN/PR | Instituto Oswaldo Cruz FIOCRUZ - Laboratory of Respiratory Viruses and Measles (LVRS) | NIC,FIOCRUZ | |
| EPI_ISL_326793 | EPI1279479 | A/H1pdm09 | Brazil | 2018-Feb-21 | A/Parana/142/2018 | Laboratório Central do Estado do Paraná - LACEN/PR | Instituto Oswaldo Cruz FIOCRUZ - Laboratory of Respiratory Viruses and Measles (LVRS) | NIC,FIOCRUZ | |
| EPI_ISL_326794 | EPI1279514 | A/H1pdm09 | Brazil | 2018-May-15 | A/Rio Grande do Sul/493/2018 | LACEN/RS - Laboratório Central de Saúde Pública do Rio Grande do Sul | Instituto Oswaldo Cruz FIOCRUZ - Laboratory of Respiratory Viruses and Measles (LVRS) | NIC,FIOCRUZ | |
| EPI_ISL_326795 | EPI1279513 | A/H1pdm09 | Brazil | 2018-Apr-25 | A/Rio Grande do Sul/425/2018 | LACEN/RS - Laboratório Central de Saúde Pública do Rio Grande do Sul | Instituto Oswaldo Cruz FIOCRUZ - Laboratory of Respiratory Viruses and Measles (LVRS) | NIC,FIOCRUZ | |
| EPI_ISL_326796 | EPI1297292 | A/H1pdm09 | Brazil | 2018-Jun-07 | A/Sergipe/621/2018 | LACEN/SE - Laboratório Central de Saúde Pública de Sergipe - Instituto Parreira Horta | Instituto Oswaldo Cruz FIOCRUZ - Laboratory of Respiratory Viruses and Measles (LVRS) | NIC,FIOCRUZ | |
| EPI_ISL_326797 | EPI1279538 | A/H1pdm09 | Brazil | 2018-Apr-23 | A/Sergipe/296/2018 | LACEN/SE - Laboratório Central de Saúde Pública de Sergipe - Instituto Parreira Horta | Instituto Oswaldo Cruz FIOCRUZ - Laboratory of Respiratory Viruses and Measles (LVRS) | NIC,FIOCRUZ | |
| EPI_ISL_328534 | EPI1279537 | A/H1pdm09 | Brazil | 2018-Apr-21 | A/Sergipe/294/2018 | LACEN/SE - Laboratório Central de Saúde Pública de Sergipe - Instituto Parreira Horta | Instituto Oswaldo Cruz FIOCRUZ - Laboratory of Respiratory Viruses and Measles (LVRS) | NIC,FIOCRUZ | |
| EPI_ISL_328536 | EPI1279536 | A/H1pdm09 | Brazil | 2018-Apr-16 | A/Sergipe/291/2018 | LACEN/SE - Laboratório Central de Saúde Pública de Sergipe - Instituto Parreira Horta | Instituto Oswaldo Cruz FIOCRUZ - Laboratory of Respiratory Viruses and Measles (LVRS) | NIC,FIOCRUZ | |
| EPI_ISL_328537 | EPI1279435 | A/H1pdm09 | Brazil | 2018-Apr-08 | A/Bahia/558/2018 | Laboratório Central de Saúde Pública Professor Gonçalo Moniz, LACEN-BA | Instituto Oswaldo Cruz FIOCRUZ - Laboratory of Respiratory Viruses and Measles (LVRS) | NIC,FIOCRUZ | |
| EPI_ISL_329886 | EPI1279434 | A/H1pdm09 | Brazil | 2018-Mar-27 | A/Bahia/276/2018 | Laboratório Central de Saúde Pública Professor Gonçalo Moniz, LACEN-BA | Instituto Oswaldo Cruz FIOCRUZ - Laboratory of Respiratory Viruses and Measles (LVRS) | NIC,FIOCRUZ | |
| EPI_ISL_329888 | EPI1279433 | A/H1pdm09 | Brazil | 2018-Mar-17 | A/Bahia/275/2018 | Laboratório Central de Saúde Pública Professor Gonçalo Moniz, LACEN-BA | Instituto Oswaldo Cruz FIOCRUZ - Laboratory of Respiratory Viruses and Measles (LVRS) | NIC,FIOCRUZ | |
| EPI_ISL_329889 | EPI1279432 | A/H1pdm09 | Brazil | 2018-Apr-02 | A/Bahia/271/2018 | Laboratório Central de Saúde Pública Professor Gonçalo Moniz, LACEN-BA | Instituto Oswaldo Cruz FIOCRUZ - Laboratory of Respiratory Viruses and Measles (LVRS) | NIC,FIOCRUZ | |
| EPI_ISL_329892 | EPI1279431 | A/H1pdm09 | Brazil | 2018-Mar-27 | A/Bahia/270/2018 | Laboratório Central de Saúde Pública Professor Gonçalo Moniz, LACEN-BA | Instituto Oswaldo Cruz FIOCRUZ - Laboratory of Respiratory Viruses and Measles (LVRS) | NIC,FIOCRUZ | |
| EPI_ISL_329895 | EPI1279430 | A/H1pdm09 | Brazil | 2018-Mar-06 | A/Bahia/121/2018 | Laboratório Central de Saúde Pública Professor Gonçalo Moniz, LACEN-BA | Instituto Oswaldo Cruz FIOCRUZ - Laboratory of Respiratory Viruses and Measles (LVRS) | NIC,FIOCRUZ | |
| EPI_ISL_329896 | EPI1279446 | A/H1pdm09 | Brazil | 2018-May-18 | A/Espirito Santo/543/2018 | LACEN/ES - Laboratório Central de Saúde Pública do Espírito Santo | Instituto Oswaldo Cruz FIOCRUZ - Laboratory of Respiratory Viruses and Measles (LVRS) | NIC,FIOCRUZ | |
| EPI_ISL_329897 | EPI1279445 | A/H1pdm09 | Brazil | 2018-May-09 | A/Espirito Santo/537/2018 | LACEN/ES - Laboratório Central de Saúde Pública do Espírito Santo | Instituto Oswaldo Cruz FIOCRUZ - Laboratory of Respiratory Viruses and Measles (LVRS) | NIC,FIOCRUZ | |
| EPI_ISL_329898 | EPI1279444 | A/H1pdm09 | Brazil | 2018-May-01 | A/Espirito Santo/534/2018 | LACEN/ES - Laboratório Central de Saúde Pública do Espírito Santo | Instituto Oswaldo Cruz FIOCRUZ - Laboratory of Respiratory Viruses and Measles (LVRS) | NIC,FIOCRUZ | |
| EPI_ISL_329903 | EPI1314246 | A/H1pdm09 | Brazil | 2018-Jul-24 | A/Rio De Janeiro/876/2018 | Instituto Oswaldo Cruz FIOCRUZ - Laboratory of Respiratory Viruses and Measles (LVRS) | Centers for Disease Control and Prevention |  | |
| EPI_ISL_329904 | EPI1314156 | A/H1pdm09 | Brazil | 2018-Jul-18 | A/Parana/1080/2018 | Instituto Oswaldo Cruz FIOCRUZ - Laboratory of Respiratory Viruses and Measles (LVRS) | Centers for Disease Control and Prevention |  | |
| EPI_ISL_329905 | EPI1314141 | A/H1pdm09 | Brazil | 2018-Jul-15 | A/Parana/1036/2018 | Instituto Oswaldo Cruz FIOCRUZ - Laboratory of Respiratory Viruses and Measles (LVRS) | Centers for Disease Control and Prevention |  | |
| EPI_ISL_329909 | EPI1314262 | A/H1pdm09 | Brazil | 2018-Jul-25 | A/Brazil/3018/2018 | Instituto Adolfo Lutz | Centers for Disease Control and Prevention |  | |
| EPI_ISL_330201 | EPI1314149 | A/H1pdm09 | Brazil | 2018-Jul-17 | A/Brazil/0805/2018 | Instituto Adolfo Lutz | Centers for Disease Control and Prevention |  | |
| EPI_ISL_330406 | EPI1314015 | A/H1pdm09 | Brazil | 2018-Jun-26 | A/Brazil/4449/2018 | Instituto Adolfo Lutz | Centers for Disease Control and Prevention |  | |
| EPI_ISL_330410 | EPI1313951 | A/H1pdm09 | Brazil | 2018-Jun-16 | A/Brazil/6340/2018 | Instituto Adolfo Lutz | Centers for Disease Control and Prevention |  | |
| EPI_ISL_330411 | EPI1313946 | A/H1pdm09 | Brazil | 2018-Jun-15 | A/Brazil/1456/2018 | Instituto Adolfo Lutz | Centers for Disease Control and Prevention |  | |
| EPI_ISL_330419 | EPI1313915 | A/H1pdm09 | Brazil | 2018-May-07 | A/Brazil/0382/2018 | Instituto Adolfo Lutz | Centers for Disease Control and Prevention |  | |
| EPI_ISL_330435 | EPI1312653 | A/H1pdm09 | Brazil | 2018-Jul-10 | A/Brazil/8539/2018 | Instituto Adolfo Lutz | Centers for Disease Control and Prevention |  | |
| EPI_ISL_330436 | EPI1311072 | A/H1pdm09 | Brazil | 2018-May-03 | A/Brazil/0015/2018 | Instituto Adolfo Lutz | Centers for Disease Control and Prevention |  | |
| EPI_ISL_330437 | EPI1311058 | A/H1pdm09 | Brazil | 2018-May-04 | A/Brazil/0059/2018 | Instituto Adolfo Lutz | Centers for Disease Control and Prevention |  | |
| EPI_ISL_330449 | EPI1310994 | A/H1pdm09 | Brazil | 2018-Apr-26 | A/Brazil/8914/2018 | Instituto Adolfo Lutz | Centers for Disease Control and Prevention |  | |
| EPI_ISL_274173 | EPI1254844 | A/H3 | Brazil | 2017-Jul-31 | A/Roraima/149002-IEC/2017 | Evandro Chagas Institute | Evandro Chagas Institute |  | |
| EPI_ISL_274174 | EPI1254842 | A/H3 | Brazil | 2017-Jul-10 | A/Roraima/148610-IEC/2017 | Evandro Chagas Institute | Evandro Chagas Institute |  | |
| EPI_ISL_274185 | EPI1254836 | A/H3 | Brazil | 2017-Jul-03 | A/Pernambuco/148978-IEC/2017 | Evandro Chagas Institute | Evandro Chagas Institute |  | |
| EPI_ISL_274186 | EPI1254834 | A/H3 | Brazil | 2017-May-08 | A/Pernambuco/148403-IEC/2017 | Evandro Chagas Institute | Evandro Chagas Institute |  | |
| EPI_ISL_274651 | EPI1180602 | A/H3 | Brazil | 2018-Jan-10 | A/Sao Paulo/690385/2018 | Instituto Adolfo Lutz | Instituto Adolfo Lutz | Santos,Katia;Silva,Daniela;Benega,Margarete;Paulino,Renato;Santos,Cecilia;Paiva,Terezinha | |
| EPI_ISL_274652 | EPI1122608 | A/H3 | Brazil | 2017-Aug-21 | A/Sao Paulo/913733/2017 | Instituto Adolfo Lutz | Instituto Adolfo Lutz | Santos,Katia;Silva,Daniela;Benega,Margarete;Paulino,Renato;Santos,Cecilia;Paiva,Terezinha | |
| EPI_ISL_274654 | EPI1036510 | A/H3 | Brazil | 2017-Jun-26 | A/Sao Paulo/548298/2017 | Instituto Adolfo Lutz | Instituto Adolfo Lutz | Santos,Katia;Silva,Daniela;Benega,Margarete;Santos,Cecilia;Paiva,Terezinha | |
| EPI_ISL_275293 | EPI1036442 | A/H3 | Brazil | 2017-Jun-29 | A/Sao Paulo/549896/2017 | Instituto Adolfo Lutz | Instituto Adolfo Lutz | Santos,Katia;Silva,Daniela;Benega,Margarete;Santos,Cecilia;Paiva,Terezinha | |
| EPI_ISL_275296 | EPI1025277 | A/H3 | Brazil | 2017-Jun-08 | A/Sao Paulo/538363/2017 | Instituto Adolfo Lutz | Instituto Adolfo Lutz | Santos,Katia;Silva,Daniela;Benega,Margarete;Santos,Cecilia;Paiva,Terezinha | |
| EPI_ISL_275297 | EPI1010366 | A/H3 | Brazil | 2017-May-08 | A/Sao Paulo/510403/2017 | Instituto Adolfo Lutz | Instituto Adolfo Lutz | Santos,Katia;Silva,Daniela;Benega,Margarete;Santos,Cecilia;Paiva,Terezinha | |
| EPI_ISL_275298 | EPI1183652 | A/H3 | Brazil | 2017-May-09 | A/Minas Gerais/594/2017 | Instituto Oswaldo Cruz FIOCRUZ - Laboratory of Respiratory Viruses and Measles (LVRS) | WHO Collaborating Centre for Reference and Research on Influenza | Deng,Y-M.; Iannello,P.; Lau,H.; Kaye,M.; Todd,A.: Komadina,N. | |
| EPI_ISL_275299 | EPI1048956 | A/H3 | Brazil | 2017-Jun-05 | A/Brazil/482/2017 | Instituto Oswaldo Cruz FIOCRUZ - Laboratory of Respiratory Viruses and Measles (LVRS) | Centers for Disease Control and Prevention |  | |
| EPI_ISL_275300 | EPI1048895 | A/H3 | Brazil | 2017-May-20 | A/Brazil/474/2017 | Instituto Oswaldo Cruz FIOCRUZ - Laboratory of Respiratory Viruses and Measles (LVRS) | Centers for Disease Control and Prevention |  | |
| EPI_ISL_275301 | EPI1048862 | A/H3 | Brazil | 2017-May-21 | A/Brazil/399/2017 | Instituto Oswaldo Cruz FIOCRUZ - Laboratory of Respiratory Viruses and Measles (LVRS) | Centers for Disease Control and Prevention |  | |
| EPI_ISL_275302 | EPI1048641 | A/H3 | Brazil | 2017-Jun-12 | A/Roraima/148126-IEC/2017 | National Influenza Center | Centers for Disease Control and Prevention |  | |
| EPI_ISL_275303 | EPI1048623 | A/H3 | Brazil | 2017-May-30 | A/Roraima/148122-IEC/2017 | National Influenza Center | Centers for Disease Control and Prevention |  | |
| EPI_ISL_275304 | EPI1048606 | A/H3 | Brazil | 2017-May-09 | A/Paraiba/147746-IEC/2017 | National Influenza Center | Centers for Disease Control and Prevention |  | |
| EPI_ISL_275305 | EPI1197181 | A/H3 | Brazil | 2017-Sep-13 | A/Brazil/4729/2017 | Instituto Adolfo Lutz | Centers for Disease Control and Prevention |  | |
| EPI_ISL_275306 | EPI1077958 | A/H3 | Brazil | 2017-Jun-29 | A/Brazil/9896/2017 | Instituto Adolfo Lutz | Centers for Disease Control and Prevention |  | |
| EPI_ISL_275307 | EPI1077951 | A/H3 | Brazil | 2017-Jun-28 | A/Brazil/9098/2017 | Instituto Adolfo Lutz | Centers for Disease Control and Prevention |  | |
| EPI_ISL_275312 | EPI1047854 | A/H3 | Brazil | 2017-May-02 | A/Brazil/5950/2017 | Instituto Adolfo Lutz | Centers for Disease Control and Prevention |  | |
| EPI_ISL_275316 | EPI1211165 | A/H3 | Brazil | 2017-Oct-10 | A/Minas Gerais/1067/2017 | FUNED MG | Instituto Oswaldo Cruz FIOCRUZ - Laboratory of Respiratory Viruses and Measles (LVRS) | NIC,FIOCRUZ | |
| EPI_ISL_299755 | EPI1188367 | A/H3 | Brazil | 2017-Dec-07 | A/Minas Gerais/1039/2017 | FUNED MG | Instituto Oswaldo Cruz FIOCRUZ - Laboratory of Respiratory Viruses and Measles (LVRS) | NIC, FIOCRUZ | |
| EPI_ISL_267071 | EPI1179720 | A/H3 | Brazil | 2017-Jun-02 | A/Minas Gerais/623/2017 | FUNED MG | Instituto Oswaldo Cruz FIOCRUZ - Laboratory of Respiratory Viruses and Measles (LVRS) | NIC, FIOCRUZ | |
| EPI_ISL_288268 | EPI1045695 | A/H3 | Brazil | 2017-Jul-14 | A/Minas Gerais/699/2017 | FUNED MG | Instituto Oswaldo Cruz FIOCRUZ - Laboratory of Respiratory Viruses and Measles (LVRS) | NIC,FIOCRUZ-RJ | |
| EPI_ISL_314559 | EPI1045694 | A/H3 | Brazil | 2017-Jun-29 | A/Minas Gerais/626/2017 | FUNED MG | Instituto Oswaldo Cruz FIOCRUZ - Laboratory of Respiratory Viruses and Measles (LVRS) | NIC,FIOCRUZ-RJ | |
| EPI_ISL_314560 | EPI1045693 | A/H3 | Brazil | 2017-Jun-27 | A/Minas Gerais/625/2017 | FUNED MG | Instituto Oswaldo Cruz FIOCRUZ - Laboratory of Respiratory Viruses and Measles (LVRS) | NIC,FIOCRUZ-RJ | |
| EPI_ISL_314563 | EPI1279531 | A/H3 | Brazil | 2018-May-21 | A/Santa Catarina/487/2018 | LACEN SC | Instituto Oswaldo Cruz FIOCRUZ - Laboratory of Respiratory Viruses and Measles (LVRS) | NIC,FIOCRUZ | |
| EPI_ISL_314564 | EPI1045709 | A/H3 | Brazil | 2017-Jun-21 | A/Santa Catarina/453/2017 | LACEN SC | Instituto Oswaldo Cruz FIOCRUZ - Laboratory of Respiratory Viruses and Measles (LVRS) | NIC,FIOCRUZ-RJ | |
| EPI_ISL_300387 | EPI1045683 | A/H3 | Brazil | 2017-Jun-01 | A/Santa Catarina/479/2017 | LACEN SC | Instituto Oswaldo Cruz FIOCRUZ - Laboratory of Respiratory Viruses and Measles (LVRS) | NIC,FIOCRUZ-RJ | |
| EPI_ISL_275740 | EPI1045682 | A/H3 | Brazil | 2017-Jun-08 | A/Santa Catarina/455/2017 | LACEN SC | Instituto Oswaldo Cruz FIOCRUZ - Laboratory of Respiratory Viruses and Measles (LVRS) | NIC,FIOCRUZ-RJ | |
| EPI_ISL_275839 | EPI1043554 | A/H3 | Brazil | 2017-Jun-11 | A/Santa Catarina/448/2017 | LACEN SC | Instituto Oswaldo Cruz FIOCRUZ - Laboratory of Respiratory Viruses and Measles (LVRS) | National Influenza Center, FIOCRUZ, Rio de Janeiro | |
| EPI_ISL_275873 | EPI1211173 | A/H3 | Brazil | 2018-Feb-25 | A/Parana/96/2018 | Laboratório Central do Estado do Paraná - LACEN/PR | Instituto Oswaldo Cruz FIOCRUZ - Laboratory of Respiratory Viruses and Measles (LVRS) | NIC,FIOCRUZ | |
| EPI_ISL_281542 | EPI1045701 | A/H3 | Brazil | 2017-Jul-22 | A/Parana/725/2017 | Laboratório Central do Estado do Paraná - LACEN/PR | Instituto Oswaldo Cruz FIOCRUZ - Laboratory of Respiratory Viruses and Measles (LVRS) | NIC,FIOCRUZ-RJ | |
| EPI_ISL_275291 | EPI1045700 | A/H3 | Brazil | 2017-Jul-13 | A/Parana/723/2017 | Laboratório Central do Estado do Paraná - LACEN/PR | Instituto Oswaldo Cruz FIOCRUZ - Laboratory of Respiratory Viruses and Measles (LVRS) | NIC,FIOCRUZ-RJ | |
| EPI_ISL_275292 | EPI1045697 | A/H3 | Brazil | 2017-Jul-11 | A/Parana/718/2017 | Laboratório Central do Estado do Paraná - LACEN/PR | Instituto Oswaldo Cruz FIOCRUZ - Laboratory of Respiratory Viruses and Measles (LVRS) | NIC,FIOCRUZ-RJ | |
| EPI_ISL_275297 | EPI1045696 | A/H3 | Brazil | 2017-Jul-10 | A/Parana/717/2017 | Laboratório Central do Estado do Paraná - LACEN/PR | Instituto Oswaldo Cruz FIOCRUZ - Laboratory of Respiratory Viruses and Measles (LVRS) | NIC,FIOCRUZ-RJ | |
| EPI_ISL_275298 | EPI1045688 | A/H3 | Brazil | 2017-Jun-12 | A/Parana/492/2017 | Laboratório Central do Estado do Paraná - LACEN/PR | Instituto Oswaldo Cruz FIOCRUZ - Laboratory of Respiratory Viruses and Measles (LVRS) | NIC,FIOCRUZ-RJ | |
| EPI_ISL_275299 | EPI1045687 | A/H3 | Brazil | 2017-Jun-10 | A/Parana/491/2017 | Laboratório Central do Estado do Paraná - LACEN/PR | Instituto Oswaldo Cruz FIOCRUZ - Laboratory of Respiratory Viruses and Measles (LVRS) | NIC,FIOCRUZ-RJ | |
| EPI_ISL_275303 | EPI1045686 | A/H3 | Brazil | 2017-Jun-19 | A/Parana/487/2017 | Laboratório Central do Estado do Paraná - LACEN/PR | Instituto Oswaldo Cruz FIOCRUZ - Laboratory of Respiratory Viruses and Measles (LVRS) | NIC,FIOCRUZ-RJ | |
| EPI_ISL_275306 | EPI1045685 | A/H3 | Brazil | 2017-Jun-06 | A/Parana/484/2017 | Laboratório Central do Estado do Paraná - LACEN/PR | Instituto Oswaldo Cruz FIOCRUZ - Laboratory of Respiratory Viruses and Measles (LVRS) | NIC,FIOCRUZ-RJ | |
| EPI_ISL_275308 | EPI1043552 | A/H3 | Brazil | 2017-May-12 | A/Parana/340/2017 | Laboratório Central do Estado do Paraná - LACEN/PR | Instituto Oswaldo Cruz FIOCRUZ - Laboratory of Respiratory Viruses and Measles (LVRS) | National Influenza Center, FIOCRUZ, Rio de Janeiro | |
| EPI_ISL_299744 | EPI1040849 | A/H3 | Brazil | 2017-May-19 | A/Parana/349/2017 | Laboratório Central do Estado do Paraná - LACEN/PR | Instituto Oswaldo Cruz FIOCRUZ - Laboratory of Respiratory Viruses and Measles (LVRS) | National Influenza Center, FIOCRUZ, Rio de Janeiro | |
| EPI_ISL_299746 | EPI1040848 | A/H3 | Brazil | 2017-May-17 | A/Parana/348/2017 | Laboratório Central do Estado do Paraná - LACEN/PR | Instituto Oswaldo Cruz FIOCRUZ - Laboratory of Respiratory Viruses and Measles (LVRS) | National Influenza Center, FIOCRUZ, Rio de Janeiro | |
| EPI_ISL_306025 | EPI1179712 | A/H3 | Brazil | 2017-Sep-01 | A/Santa Catarina/859/2017 | LACEN/SC | Instituto Oswaldo Cruz FIOCRUZ - Laboratory of Respiratory Viruses and Measles (LVRS) | NIC, FIOCRUZ | |
| EPI_ISL_299888 | EPI1043549 | A/H3 | Brazil | 2017-May-23 | A/Santa Catarina/475/2017 | LACEN/SC | Instituto Oswaldo Cruz FIOCRUZ - Laboratory of Respiratory Viruses and Measles (LVRS) | Resende,PC | |
| EPI_ISL_275841 | EPI1179708 | A/H3 | Brazil | 2017-Jun-22 | A/Rio Grande do Sul/660/2017 | LACEN/RS - Laboratório Central de Saúde Pública do Rio Grande do Sul | Instituto Oswaldo Cruz FIOCRUZ - Laboratory of Respiratory Viruses and Measles (LVRS) | NIC, FIOCRUZ | |
| EPI_ISL_303087 | EPI1179706 | A/H3 | Brazil | 2017-Jun-07 | A/Rio Grande do Sul/653/2017 | LACEN/RS - Laboratório Central de Saúde Pública do Rio Grande do Sul | Instituto Oswaldo Cruz FIOCRUZ - Laboratory of Respiratory Viruses and Measles (LVRS) | NIC, FIOCRUZ | |
| EPI_ISL_274653 | EPI1045681 | A/H3 | Brazil | 2017-Jul-06 | A/Rio Grande do Sul/671/2017 | LACEN/RS - Laboratório Central de Saúde Pública do Rio Grande do Sul | Instituto Oswaldo Cruz FIOCRUZ - Laboratory of Respiratory Viruses and Measles (LVRS) | NIC,FIOCRUZ-RJ | |
| EPI_ISL_274655 | EPI1045680 | A/H3 | Brazil | 2017-Jul-19 | A/Rio Grande do Sul/667/2017 | LACEN/RS - Laboratório Central de Saúde Pública do Rio Grande do Sul | Instituto Oswaldo Cruz FIOCRUZ - Laboratory of Respiratory Viruses and Measles (LVRS) | NIC,FIOCRUZ-RJ | |
| EPI_ISL_274660 | EPI1043551 | A/H3 | Brazil | 2017-May-11 | A/Rio Grande do Sul/287/2017 | LACEN/RS - Laboratório Central de Saúde Pública do Rio Grande do Sul | Instituto Oswaldo Cruz FIOCRUZ - Laboratory of Respiratory Viruses and Measles (LVRS) | National Influenza Center, FIOCRUZ, Rio de Janeiro | |
| EPI_ISL_275294 | EPI1043550 | A/H3 | Brazil | 2017-May-07 | A/Rio Grande do Sul/286/2017 | LACEN/RS - Laboratório Central de Saúde Pública do Rio Grande do Sul | Instituto Oswaldo Cruz FIOCRUZ - Laboratory of Respiratory Viruses and Measles (LVRS) | National Influenza Center, FIOCRUZ, Rio de Janeiro | |
| EPI_ISL_275311 | EPI1043548 | A/H3 | Brazil | 2017-May-09 | A/Sergipe/251/2017 | LACEN/SE - Laboratório Central de Saúde Pública de Sergipe - Instituto Parreira Horta | Instituto Oswaldo Cruz FIOCRUZ - Laboratory of Respiratory Viruses and Measles (LVRS) | National Influenza Center, FIOCRUZ, Rio de Janeiro | |
| EPI_ISL_299735 | EPI1040862 | A/H3 | Brazil | 2017-May-22 | A/Sergipe/319/2017 | LACEN/SE - Laboratório Central de Saúde Pública de Sergipe - Instituto Parreira Horta | Instituto Oswaldo Cruz FIOCRUZ - Laboratory of Respiratory Viruses and Measles (LVRS) | National Influenza Center, FIOCRUZ, Rio de Janeiro | |
| EPI_ISL_299736 | EPI1040861 | A/H3 | Brazil | 2017-May-03 | A/Sergipe/243/2017 | LACEN/SE - Laboratório Central de Saúde Pública de Sergipe - Instituto Parreira Horta | Instituto Oswaldo Cruz FIOCRUZ - Laboratory of Respiratory Viruses and Measles (LVRS) | National Influenza Center, FIOCRUZ, Rio de Janeiro | |
| EPI_ISL_299750 | EPI1179695 | A/H3 | Brazil | 2017-May-21 | A/Bahia/674/2017 | Laboratório Central de Saúde Pública Professor Gonçalo Moniz, LACEN-BA | Instituto Oswaldo Cruz FIOCRUZ - Laboratory of Respiratory Viruses and Measles (LVRS) | NIC, FIOCRUZ | |
| EPI_ISL_301363 | EPI1179697 | A/H3 | Brazil | 2017-May-28 | A/Espirito Santo/619/2017 | LACEN/ES - Laboratório Central de Saúde Pública do Espírito Santo | Instituto Oswaldo Cruz FIOCRUZ - Laboratory of Respiratory Viruses and Measles (LVRS) | NIC, FIOCRUZ | |
| EPI_ISL_306033 | EPI1045692 | A/H3 | Brazil | 2017-Jun-07 | A/Espirito Santo/506/2017 | LACEN/ES - Laboratório Central de Saúde Pública do Espírito Santo | Instituto Oswaldo Cruz FIOCRUZ - Laboratory of Respiratory Viruses and Measles (LVRS) | NIC,FIOCRUZ-RJ | |
| EPI_ISL_322297 | EPI1045691 | A/H3 | Brazil | 2017-Jun-03 | A/Espirito Santo/503/2017 | LACEN/ES - Laboratório Central de Saúde Pública do Espírito Santo | Instituto Oswaldo Cruz FIOCRUZ - Laboratory of Respiratory Viruses and Measles (LVRS) | NIC,FIOCRUZ-RJ | |
| EPI_ISL_269968 | EPI1045690 | A/H3 | Brazil | 2017-Jun-03 | A/Espirito Santo/502/2017 | LACEN/ES - Laboratório Central de Saúde Pública do Espírito Santo | Instituto Oswaldo Cruz FIOCRUZ - Laboratory of Respiratory Viruses and Measles (LVRS) | NIC,FIOCRUZ-RJ | |
| EPI_ISL_273300 | EPI1045689 | A/H3 | Brazil | 2017-Jun-03 | A/Espirito Santo/500/2017 | LACEN/ES - Laboratório Central de Saúde Pública do Espírito Santo | Instituto Oswaldo Cruz FIOCRUZ - Laboratory of Respiratory Viruses and Measles (LVRS) | NIC,FIOCRUZ-RJ | |
| EPI_ISL_273328 | EPI1241335 | A/H3 | Brazil | 2018-Mar-07 | A/Sao Paulo/734612/2018 | Instituto Adolfo Lutz | Instituto Adolfo Lutz | Santos,Katia;Silva,Daniela;Benega,Margarete;Santos,Cecilia;Paiva,Terezinha | |
| EPI_ISL_275837 | EPI1036510 | A/H3 | Brazil | 2017-Jun-26 | A/Sao Paulo/548298/2017 | Instituto Adolfo Lutz | Instituto Adolfo Lutz | Santos,Katia;Silva,Daniela;Benega,Margarete;Santos,Cecilia;Paiva,Terezinha | |
| EPI_ISL_275869 | EPI1025262 | A/H3 | Brazil | 2017-Jun-09 | A/Mato Grosso do Sul/554594/2017 | Instituto Adolfo Lutz | Instituto Adolfo Lutz | Santos,Katia;Silva,Daniela;Benega,Margarete;Santos,Cecilia;Paiva,Terezinha | |
| EPI_ISL_275881 | EPI1141907 | A/H3 | Brazil | 2017-Jul-10 | A/Rio Grande Do Sul/663/2017 | Instituto Oswaldo Cruz FIOCRUZ - Laboratory of Respiratory Viruses and Measles (LVRS) | Centers for Disease Control and Prevention |  | |
| EPI_ISL_281543 | EPI1048956 | A/H3 | Brazil | 2017-Jun-05 | A/Brazil/482/2017 | Instituto Oswaldo Cruz FIOCRUZ - Laboratory of Respiratory Viruses and Measles (LVRS) | Centers for Disease Control and Prevention |  | |
| EPI_ISL_281545 | EPI1048862 | A/H3 | Brazil | 2017-May-21 | A/Brazil/399/2017 | Instituto Oswaldo Cruz FIOCRUZ - Laboratory of Respiratory Viruses and Measles (LVRS) | Centers for Disease Control and Prevention |  | |
| EPI_ISL_322967 | EPI1048606 | A/H3 | Brazil | 2017-May-09 | A/Paraiba/147746-IEC/2017 | National Influenza Center | Centers for Disease Control and Prevention |  | |
| EPI_ISL_275317 | EPI1282926 | A/H3 | Brazil | 2018-Jun-12 | A/Brazil/5557/2018 | Instituto Adolfo Lutz | Centers for Disease Control and Prevention |  | |
| EPI_ISL_269965 | EPI1077974 | A/H3 | Brazil | 2017-Jul-10 | A/Brazil/7516/2017 | Instituto Adolfo Lutz | Centers for Disease Control and Prevention |  | |
| EPI_ISL_311846 | EPI1077958 | A/H3 | Brazil | 2017-Jun-29 | A/Brazil/9896/2017 | Instituto Adolfo Lutz | Centers for Disease Control and Prevention |  | |
| EPI_ISL_291644 | EPI1279468 | A/H3 | Brazil | 2018-May-11 | A/Minas Gerais/452/2018 | FUNED MG | Instituto Oswaldo Cruz FIOCRUZ - Laboratory of Respiratory Viruses and Measles (LVRS) | NIC,FIOCRUZ | |
| EPI_ISL_274158 | EPI1279467 | A/H3 | Brazil | 2018-Apr-20 | A/Minas Gerais/449/2018 | FUNED MG | Instituto Oswaldo Cruz FIOCRUZ - Laboratory of Respiratory Viruses and Measles (LVRS) | NIC,FIOCRUZ | |
| EPI_ISL_274171 | EPI1279466 | A/H3 | Brazil | 2018-Apr-25 | A/Minas Gerais/446/2018 | FUNED MG | Instituto Oswaldo Cruz FIOCRUZ - Laboratory of Respiratory Viruses and Measles (LVRS) | NIC,FIOCRUZ | |
| EPI_ISL_275289 | EPI1279465 | A/H3 | Brazil | 2018-Apr-24 | A/Minas Gerais/443/2018 | FUNED MG | Instituto Oswaldo Cruz FIOCRUZ - Laboratory of Respiratory Viruses and Measles (LVRS) | NIC,FIOCRUZ | |
| EPI_ISL_275295 | EPI1279464 | A/H3 | Brazil | 2018-Apr-19 | A/Minas Gerais/442/2018 | FUNED MG | Instituto Oswaldo Cruz FIOCRUZ - Laboratory of Respiratory Viruses and Measles (LVRS) | NIC,FIOCRUZ | |
| EPI_ISL_275625 | EPI1279462 | A/H3 | Brazil | 2018-Apr-09 | A/Minas Gerais/438/2018 | FUNED MG | Instituto Oswaldo Cruz FIOCRUZ - Laboratory of Respiratory Viruses and Measles (LVRS) | NIC,FIOCRUZ | |
| EPI_ISL_299734 | EPI1279461 | A/H3 | Brazil | 2018-Mar-05 | A/Minas Gerais/135/2018 | FUNED MG | Instituto Oswaldo Cruz FIOCRUZ - Laboratory of Respiratory Viruses and Measles (LVRS) | NIC,FIOCRUZ | |
| EPI_ISL_299739 | EPI1188379 | A/H3 | Brazil | 2018-Jan-13 | A/Minas Gerais/39/2018 | FUNED MG | Instituto Oswaldo Cruz FIOCRUZ - Laboratory of Respiratory Viruses and Measles (LVRS) | NIC, FIOCRUZ | |
| EPI_ISL_299741 | EPI1188371 | A/H3 | Brazil | 2017-Oct-04 | A/Minas Gerais/1058/2017 | FUNED MG | Instituto Oswaldo Cruz FIOCRUZ - Laboratory of Respiratory Viruses and Measles (LVRS) | NIC, FIOCRUZ | |
| EPI_ISL_299745 | EPI1188369 | A/H3 | Brazil | 2017-Dec-01 | A/Minas Gerais/1049/2017 | FUNED MG | Instituto Oswaldo Cruz FIOCRUZ - Laboratory of Respiratory Viruses and Measles (LVRS) | NIC, FIOCRUZ | |
| EPI_ISL_299751 | EPI1211177 | A/H3 | Brazil | 2018-Mar-07 | A/Rio de Janeiro/98/2018 | Laboratório Central de Saúde Pública (LACEN RJ) | Instituto Oswaldo Cruz FIOCRUZ - Laboratory of Respiratory Viruses and Measles (LVRS) | NIC,FIOCRUZ | |
| EPI_ISL_299756 | EPI1211178 | A/H3 | Brazil | 2018-Feb-14 | A/Santa Catarina/62/2018 | LACEN SC | Instituto Oswaldo Cruz FIOCRUZ - Laboratory of Respiratory Viruses and Measles (LVRS) | NIC,FIOCRUZ | |
| EPI_ISL_299757 | EPI1188377 | A/H3 | Brazil | 2018-Jan-17 | A/Santa Catarina/25/2018 | LACEN SC | Instituto Oswaldo Cruz FIOCRUZ - Laboratory of Respiratory Viruses and Measles (LVRS) | NIC, FIOCRUZ | |
| EPI_ISL_301365 | EPI1188376 | A/H3 | Brazil | 2018-Jan-05 | A/Santa Catarina/24/2018 | LACEN SC | Instituto Oswaldo Cruz FIOCRUZ - Laboratory of Respiratory Viruses and Measles (LVRS) | NIC, FIOCRUZ | |
| EPI_ISL_301367 | EPI1045711 | A/H3 | Brazil | 2017-Jun-14 | A/Santa Catarina/454/2017 | LACEN SC | Instituto Oswaldo Cruz FIOCRUZ - Laboratory of Respiratory Viruses and Measles (LVRS) | NIC,FIOCRUZ-RJ | |
| EPI_ISL_301368 | EPI1279497 | A/H3 | Brazil | 2018-Apr-23 | A/Parana/456/2018 | Laboratório Central do Estado do Paraná - LACEN/PR | Instituto Oswaldo Cruz FIOCRUZ - Laboratory of Respiratory Viruses and Measles (LVRS) | NIC,FIOCRUZ | |
| EPI_ISL_301370 | EPI1279496 | A/H3 | Brazil | 2018-Apr-05 | A/Parana/329/2018 | Laboratório Central do Estado do Paraná - LACEN/PR | Instituto Oswaldo Cruz FIOCRUZ - Laboratory of Respiratory Viruses and Measles (LVRS) | NIC,FIOCRUZ | |
| EPI_ISL_301371 | EPI1279494 | A/H3 | Brazil | 2018-May-01 | A/Parana/321/2018 | Laboratório Central do Estado do Paraná - LACEN/PR | Instituto Oswaldo Cruz FIOCRUZ - Laboratory of Respiratory Viruses and Measles (LVRS) | NIC,FIOCRUZ | |
| EPI_ISL_301372 | EPI1279493 | A/H3 | Brazil | 2018-Apr-24 | A/Parana/318/2018 | Laboratório Central do Estado do Paraná - LACEN/PR | Instituto Oswaldo Cruz FIOCRUZ - Laboratory of Respiratory Viruses and Measles (LVRS) | NIC,FIOCRUZ | |
| EPI_ISL_301373 | EPI1279492 | A/H3 | Brazil | 2018-Apr-12 | A/Parana/313/2018 | Laboratório Central do Estado do Paraná - LACEN/PR | Instituto Oswaldo Cruz FIOCRUZ - Laboratory of Respiratory Viruses and Measles (LVRS) | NIC,FIOCRUZ | |
| EPI_ISL_301374 | EPI1279491 | A/H3 | Brazil | 2018-Mar-22 | A/Parana/158/2018 | Laboratório Central do Estado do Paraná - LACEN/PR | Instituto Oswaldo Cruz FIOCRUZ - Laboratory of Respiratory Viruses and Measles (LVRS) | NIC,FIOCRUZ | |
| EPI_ISL_301375 | EPI1211170 | A/H3 | Brazil | 2018-Jan-26 | A/Parana/59/2018 | Laboratório Central do Estado do Paraná - LACEN/PR | Instituto Oswaldo Cruz FIOCRUZ - Laboratory of Respiratory Viruses and Measles (LVRS) | NIC,FIOCRUZ | |
| EPI_ISL_306027 | EPI1211169 | A/H3 | Brazil | 2018-Jan-20 | A/Parana/57/2018 | Laboratório Central do Estado do Paraná - LACEN/PR | Instituto Oswaldo Cruz FIOCRUZ - Laboratory of Respiratory Viruses and Measles (LVRS) | NIC,FIOCRUZ | |
| EPI_ISL_306029 | EPI1211167 | A/H3 | Brazil | 2018-Jan-09 | A/Parana/52/2018 | Laboratório Central do Estado do Paraná - LACEN/PR | Instituto Oswaldo Cruz FIOCRUZ - Laboratory of Respiratory Viruses and Measles (LVRS) | NIC,FIOCRUZ | |
| EPI_ISL_306030 | EPI1188381 | A/H3 | Brazil | 2018-Jan-17 | A/Parana/56/2018 | Laboratório Central do Estado do Paraná - LACEN/PR | Instituto Oswaldo Cruz FIOCRUZ - Laboratory of Respiratory Viruses and Measles (LVRS) | NIC, FIOCRUZ | |
| EPI_ISL_306036 | EPI1188380 | A/H3 | Brazil | 2018-Jan-19 | A/Parana/54/2018 | Laboratório Central do Estado do Paraná - LACEN/PR | Instituto Oswaldo Cruz FIOCRUZ - Laboratory of Respiratory Viruses and Measles (LVRS) | NIC, FIOCRUZ | |
| EPI_ISL_306037 | EPI1179722 | A/H3 | Brazil | 2017-Dec-02 | A/Parana/1027/2017 | Laboratório Central do Estado do Paraná - LACEN/PR | Instituto Oswaldo Cruz FIOCRUZ - Laboratory of Respiratory Viruses and Measles (LVRS) | NIC, FIOCRUZ | |
| EPI_ISL_313433 | EPI1179721 | A/H3 | Brazil | 2017-Nov-23 | A/Parana/1025/2017 | Laboratório Central do Estado do Paraná - LACEN/PR | Instituto Oswaldo Cruz FIOCRUZ - Laboratory of Respiratory Viruses and Measles (LVRS) | NIC, FIOCRUZ | |
| EPI_ISL_322194 | EPI1179702 | A/H3 | Brazil | 2017-Sep-25 | A/Parana/892/2017 | Laboratório Central do Estado do Paraná - LACEN/PR | Instituto Oswaldo Cruz FIOCRUZ - Laboratory of Respiratory Viruses and Measles (LVRS) | NIC, FIOCRUZ | |
| EPI_ISL_322215 | EPI1179700 | A/H3 | Brazil | 2017-Dec-11 | A/Parana/1029/2017 | Laboratório Central do Estado do Paraná - LACEN/PR | Instituto Oswaldo Cruz FIOCRUZ - Laboratory of Respiratory Viruses and Measles (LVRS) | NIC, FIOCRUZ | |
| EPI_ISL_322216 | EPI1054521 | A/H3 | Brazil | 2017-Jun-20 | A/Parana/489/2017 | Laboratório Central do Estado do Paraná - LACEN/PR | Instituto Oswaldo Cruz FIOCRUZ - Laboratory of Respiratory Viruses and Measles (LVRS) | NIC,FIOCRUZ-RJ | |
| EPI_ISL_322217 | EPI1045684 | A/H3 | Brazil | 2017-May-31 | A/Parana/481/2017 | Laboratório Central do Estado do Paraná - LACEN/PR | Instituto Oswaldo Cruz FIOCRUZ - Laboratory of Respiratory Viruses and Measles (LVRS) | NIC,FIOCRUZ-RJ | |
| EPI_ISL_322218 | EPI1040846 | A/H3 | Brazil | 2017-May-02 | A/Parana/339/2017 | Laboratório Central do Estado do Paraná - LACEN/PR | Instituto Oswaldo Cruz FIOCRUZ - Laboratory of Respiratory Viruses and Measles (LVRS) | National Influenza Center, FIOCRUZ, Rio de Janeiro | |
| EPI_ISL_322219 | EPI1179713 | A/H3 | Brazil | 2017-Nov-10 | A/Santa Catarina/961/2017 | LACEN/SC | Instituto Oswaldo Cruz FIOCRUZ - Laboratory of Respiratory Viruses and Measles (LVRS) | NIC, FIOCRUZ | |
| EPI_ISL_322220 | EPI1179707 | A/H3 | Brazil | 2017-Jun-09 | A/Rio Grande do Sul/655/2017 | LACEN/RS - Laboratório Central de Saúde Pública do Rio Grande do Sul | Instituto Oswaldo Cruz FIOCRUZ - Laboratory of Respiratory Viruses and Measles (LVRS) | NIC, FIOCRUZ | |
| EPI_ISL_322221 | EPI1045678 | A/H3 | Brazil | 2017-Jul-04 | A/Rio Grande do Sul/661/2017 | LACEN/RS - Laboratório Central de Saúde Pública do Rio Grande do Sul | Instituto Oswaldo Cruz FIOCRUZ - Laboratory of Respiratory Viruses and Measles (LVRS) | NIC,FIOCRUZ-RJ | |
| EPI_ISL_322222 | EPI1040832 | A/H3 | Brazil | 2017-May-18 | A/Rio Grande do Sul/290/2017 | LACEN/RS - Laboratório Central de Saúde Pública do Rio Grande do Sul | Instituto Oswaldo Cruz FIOCRUZ - Laboratory of Respiratory Viruses and Measles (LVRS) | National Influenza Center, FIOCRUZ, Rio de Janeiro | |
| EPI_ISL_322223 | EPI1279436 | A/H3 | Brazil | 2018-Feb-16 | A/Bahia/105/2018 | Laboratório Central de Saúde Pública Professor Gonçalo Moniz, LACEN-BA | Instituto Oswaldo Cruz FIOCRUZ - Laboratory of Respiratory Viruses and Measles (LVRS) | NIC,FIOCRUZ | |
| EPI_ISL_322228 | EPI1179693 | A/H3 | Brazil | 2018-Jan-17 | A/Bahia/21/2018 | Laboratório Central de Saúde Pública Professor Gonçalo Moniz, LACEN-BA | Instituto Oswaldo Cruz FIOCRUZ - Laboratory of Respiratory Viruses and Measles (LVRS) | NIC, FIOCRUZ | |
| EPI_ISL_322229 | EPI1279456 | A/H3 | Brazil | 2018-May-24 | A/Espirito Santo/733/2018 | LACEN/ES - Laboratório Central de Saúde Pública do Espírito Santo | Instituto Oswaldo Cruz FIOCRUZ - Laboratory of Respiratory Viruses and Measles (LVRS) | NIC,FIOCRUZ | |
| EPI_ISL_322231 | EPI1279455 | A/H3 | Brazil | 2018-Apr-26 | A/Espirito Santo/382/2018 | LACEN/ES - Laboratório Central de Saúde Pública do Espírito Santo | Instituto Oswaldo Cruz FIOCRUZ - Laboratory of Respiratory Viruses and Measles (LVRS) | NIC,FIOCRUZ | |
| EPI_ISL_322232 | EPI1279454 | A/H3 | Brazil | 2018-Apr-26 | A/Espirito Santo/380/2018 | LACEN/ES - Laboratório Central de Saúde Pública do Espírito Santo | Instituto Oswaldo Cruz FIOCRUZ - Laboratory of Respiratory Viruses and Measles (LVRS) | NIC,FIOCRUZ | |
| EPI_ISL_322233 | EPI1279453 | A/H3 | Brazil | 2018-Apr-22 | A/Espirito Santo/378/2018 | LACEN/ES - Laboratório Central de Saúde Pública do Espírito Santo | Instituto Oswaldo Cruz FIOCRUZ - Laboratory of Respiratory Viruses and Measles (LVRS) | NIC,FIOCRUZ | |
| EPI_ISL_322234 | EPI1279452 | A/H3 | Brazil | 2018-Apr-23 | A/Espirito Santo/376/2018 | LACEN/ES - Laboratório Central de Saúde Pública do Espírito Santo | Instituto Oswaldo Cruz FIOCRUZ - Laboratory of Respiratory Viruses and Measles (LVRS) | NIC,FIOCRUZ | |
| EPI_ISL_322235 | EPI1279451 | A/H3 | Brazil | 2018-Apr-14 | A/Espirito Santo/374/2018 | LACEN/ES - Laboratório Central de Saúde Pública do Espírito Santo | Instituto Oswaldo Cruz FIOCRUZ - Laboratory of Respiratory Viruses and Measles (LVRS) | NIC,FIOCRUZ | |
| EPI_ISL_322258 | EPI1279450 | A/H3 | Brazil | 2018-Apr-06 | A/Espirito Santo/373/2018 | LACEN/ES - Laboratório Central de Saúde Pública do Espírito Santo | Instituto Oswaldo Cruz FIOCRUZ - Laboratory of Respiratory Viruses and Measles (LVRS) | NIC,FIOCRUZ | |
| EPI_ISL_322259 | EPI1279449 | A/H3 | Brazil | 2018-Apr-09 | A/Espirito Santo/372/2018 | LACEN/ES - Laboratório Central de Saúde Pública do Espírito Santo | Instituto Oswaldo Cruz FIOCRUZ - Laboratory of Respiratory Viruses and Measles (LVRS) | NIC,FIOCRUZ | |
| EPI_ISL_322260 | EPI1279448 | A/H3 | Brazil | 2018-Apr-10 | A/Espirito Santo/371/2018 | LACEN/ES - Laboratório Central de Saúde Pública do Espírito Santo | Instituto Oswaldo Cruz FIOCRUZ - Laboratory of Respiratory Viruses and Measles (LVRS) | NIC,FIOCRUZ | |
| EPI_ISL_322261 | EPI1188373 | A/H3 | Brazil | 2017-Dec-27 | A/Espirito Santo/1059/2017 | LACEN/ES - Laboratório Central de Saúde Pública do Espírito Santo | Instituto Oswaldo Cruz FIOCRUZ - Laboratory of Respiratory Viruses and Measles (LVRS) | NIC, FIOCRUZ | |
| EPI_ISL_322263 | EPI1279426 | A/H3 | Brazil | 2018-May-14 | A/Alagoas/519/2018 | Laboratório Central de Saúde Pública de Alagoas, LACEN-AL | Instituto Oswaldo Cruz FIOCRUZ - Laboratory of Respiratory Viruses and Measles (LVRS) | NIC,FIOCRUZ | |
| EPI_ISL_322264 | EPI1188378 | A/H3 | Brazil | 2018-Jan-16 | A/Alagoas/35/2018 | Laboratório Central de Saúde Pública de Alagoas, LACEN-AL | Instituto Oswaldo Cruz FIOCRUZ - Laboratory of Respiratory Viruses and Measles (LVRS) | NIC, FIOCRUZ | |
| EPI_ISL_322265 | EPI1305014 | A/H3 | Brazil | 2018-Mar-21 | A/Amazonas/152018-IEC/2018 | Evandro Chagas Institute | Evandro Chagas Institute |  | |
| EPI_ISL_322266 | EPI1305010 | A/H3 | Brazil | 2018-Apr-24 | A/Ceara/152553-IEC/2018 | Evandro Chagas Institute | Evandro Chagas Institute |  | |
| EPI_ISL_322267 | EPI1305006 | A/H3 | Brazil | 2018-Apr-23 | A/Paraiba/152256-IEC/2018 | Evandro Chagas Institute | Evandro Chagas Institute |  | |
| EPI_ISL_322269 | EPI1305004 | A/H3 | Brazil | 2018-Mar-28 | A/Paraiba/151837-IEC/2018 | Evandro Chagas Institute | Evandro Chagas Institute |  | |
| EPI_ISL_322272 | EPI1254828 | A/H3 | Brazil | 2018-Mar-13 | A/Para/151723-IEC/2018 | Evandro Chagas Institute | Evandro Chagas Institute |  | |
| EPI_ISL_322273 | EPI1267587 | A/H3 | Brazil | 2018-Mar-26 | A/Goias/898260/2018 | Instituto Adolfo Lutz | Instituto Adolfo Lutz | Santos,Katia;Silva,Daniela;Benega,Margarete;Paulino,Renato;Santos,Cecilia;Paiva,Terezinha | |
| EPI_ISL_322274 | EPI1267586 | A/H3 | Brazil | 2018-May-08 | A/Mato Grosso do Sul/521748/2018 | Instituto Adolfo Lutz | Instituto Adolfo Lutz | Santos,Katia;Silva,Daniela;Benega,Margarete;Paulino,Renato;Santos,Cecilia;Paiva,Terezinha | |
| EPI_ISL_322275 | EPI1267585 | A/H3 | Brazil | 2018-Apr-18 | A/Sao Paulo/770596/2018 | Instituto Adolfo Lutz | Instituto Adolfo Lutz | Santos,Katia;Silva,Daniela;Benega,Margarete;Paulino,Renato;Santos,Cecilia;Paiva,Terezinha | |
| EPI_ISL_322281 | EPI1267584 | A/H3 | Brazil | 2018-Mar-09 | A/Sao Paulo/736829/2018 | Instituto Adolfo Lutz | Instituto Adolfo Lutz | Santos,Katia;Silva,Daniela;Benega,Margarete;Paulino,Renato;Santos,Cecilia;Paiva,Terezinha | |
| EPI_ISL_322289 | EPI1241334 | A/H3 | Brazil | 2018-Feb-01 | A/Sao Paulo/706496/2018 | Instituto Adolfo Lutz | Instituto Adolfo Lutz | Santos,Katia;Silva,Daniela;Benega,Margarete;Santos,Cecilia;Paiva,Terezinh | |
| EPI_ISL_322290 | EPI1241333 | A/H3 | Brazil | 2018-Mar-06 | A/Sao Paulo/733190/2018 | Instituto Adolfo Lutz | Instituto Adolfo Lutz | Santos,Katia;Silva,Daniela;Benega,Margarete;Santos,Cecilia;Paiva,Terezinh | |
| EPI_ISL_322296 | EPI1238765 | A/H3 | Brazil | 2018-Feb-15 | A/Distrito Federal/756373/2018 | Instituto Adolfo Lutz | Instituto Adolfo Lutz | Santos,Katia;Silva,Daniela;Benega,Margarete;Santos,Cecilia;Paiva,Terezinha | |
| EPI_ISL_322298 | EPI1184051 | A/H3 | Brazil | 2018-Jan-08 | A/Goias/852896/2018 | Instituto Adolfo Lutz | Instituto Adolfo Lutz | Santos,Katia;Silva,Daniela;Benega,Margarete;Paulino,Renato;Santos,Cecilia;Paiva,Terezinha | |
| EPI_ISL_283620 | EPI1184050 | A/H3 | Brazil | 2017-Dec-06 | A/Sao Paulo/1054970/2017 | Instituto Adolfo Lutz | Instituto Adolfo Lutz | Santos,Katia;Silva,Daniela;Benega,Margarete;Paulino,Renato;Santos,Cecilia;Paiva,Terezinha | |
| EPI_ISL_283674 | EPI1180603 | A/H3 | Brazil | 2018-Jan-12 | A/Sao Paulo/691063/2018 | Instituto Adolfo Lutz | Instituto Adolfo Lutz | Santos,Katia;Silva,Daniela;Benega,Margarete;Paulino,Renato;Santos,Cecilia;Paiva,Terezinha | |
| EPI_ISL_283675 | EPI1180601 | A/H3 | Brazil | 2017-Dec-06 | A/Sao Paulo/669992/2017 | Instituto Adolfo Lutz | Instituto Adolfo Lutz | Santos,Katia;Silva,Daniela;Benega,Margarete;Paulino,Renato;Santos,Cecilia;Paiva,Terezinha | |
| EPI_ISL_283676 | EPI1180600 | A/H3 | Brazil | 2017-Nov-25 | A/Sao Paulo/662138/2017 | Instituto Adolfo Lutz | Instituto Adolfo Lutz | Santos,Katia;Silva,Daniela;Benega,Margarete;Paulino,Renato;Santos,Cecilia;Paiva,Terezinha | |
| EPI_ISL_288265 | EPI1179313 | A/H3 | Brazil | 2017-Nov-13 | A/Sao Paulo/656009/2017 | Instituto Adolfo Lutz | Instituto Adolfo Lutz | Santos,Katia;Silva,Daniela;Benega,Margarete;Paulino,Renato;Santos,Cecilia;Paiva,Terezinha | |
| EPI_ISL_288266 | EPI1122717 | A/H3 | Brazil | 2017-Sep-15 | A/SAO PAULO/603559/2017 | Instituto Adolfo Lutz | Instituto Adolfo Lutz | Santos,Katia;Silva,Daniela;Benega,Margarete;Paulino,Renato;Santos,Cecilia;Paiva,Terezinha | |
| EPI_ISL_288267 | EPI1122610 | A/H3 | Brazil | 2017-Sep-22 | A/Goias/388164/2017 | Instituto Adolfo Lutz | Instituto Adolfo Lutz | Santos,Katia;Silva,Daniela;Benega,Margarete;Paulino,Renato;Santos,Cecilia;Paiva,Terezinha | |
| EPI_ISL_288270 | EPI1122607 | A/H3 | Brazil | 2017-Aug-17 | A/Sao Paulo/911967/2017 | Instituto Adolfo Lutz | Instituto Adolfo Lutz | Santos,Katia;Silva,Daniela;Benega,Margarete;Paulino,Renato;Santos,Cecilia;Paiva,Terezinha | |
| EPI_ISL_288286 | EPI1122606 | A/H3 | Brazil | 2017-Sep-26 | A/Sao Paulo/618711/2017 | Instituto Adolfo Lutz | Instituto Adolfo Lutz | Santos,Katia;Silva,Daniela;Benega,Margarete;Paulino,Renato;Santos,Cecilia;Paiva,Terezinha | |
| EPI_ISL_299596 | EPI1122605 | A/H3 | Brazil | 2017-Sep-27 | A/Sao Paulo/614558/2017 | Instituto Adolfo Lutz | Instituto Adolfo Lutz | Santos,Katia;Silva,Daniela;Benega,Margarete;Paulino,Renato;Santos,Cecilia;Paiva,Terezinha | |
| EPI_ISL_299886 | EPI1091628 | A/H3 | Brazil | 2017-Oct-05 | A/Sao Paulo/625769/2017 | Instituto Adolfo Lutz | Instituto Adolfo Lutz | Santos,Katia;Silva,Daniela;Benega,Margarete;Paulino,Renato;Santos,Cecilia;Paiva,Terezinha | |
| EPI_ISL_299887 | EPI1091627 | A/H3 | Brazil | 2017-Sep-04 | A/Sao Paulo/596258/2017 | Instituto Adolfo Lutz | Instituto Adolfo Lutz | Santos,Katia;Silva,Daniela;Benega,Margarete;Paulino,Renato;Santos,Cecilia;Paiva,Terezinha | |
| EPI_ISL_299889 | EPI1091626 | A/H3 | Brazil | 2017-Aug-29 | A/Sao Paulo/591445/2017 | Instituto Adolfo Lutz | Instituto Adolfo Lutz | Santos,Katia;Silva,Daniela;Benega,Margarete;Paulino,Renato;Santos,Cecilia;Paiva,Terezinha | |
| EPI_ISL_300563 | EPI1091212 | A/H3 | Brazil | 2017-Aug-23 | A/Goias/383452/2017 | Instituto Adolfo Lutz | Instituto Adolfo Lutz | Santos,Katia;Silva,Daniela;Benega,Margarete;Paulino,Renato;Santos,Cecilia;Paiva,Terezinha | |
| EPI_ISL_300564 | EPI1252014 | A/H3 | Brazil | 2018-Jan-13 | A/Minas Gerais/39/2018 | Instituto Oswaldo Cruz FIOCRUZ - Laboratory of Respiratory Viruses and Measles (LVRS) | Centers for Disease Control and Prevention |  | |
| EPI_ISL_311505 | EPI1249150 | A/H3 | Brazil | 2018-Feb-26 | A/Rio Grande Do Sul/103/2018 | Instituto Oswaldo Cruz FIOCRUZ - Laboratory of Respiratory Viruses and Measles (LVRS) | Centers for Disease Control and Prevention |  | |
| EPI_ISL_311844 | EPI1205274 | A/H3 | Brazil | 2018-Jan-03 | A/Rio De Janeiro/01/2018 | Instituto Oswaldo Cruz FIOCRUZ - Laboratory of Respiratory Viruses and Measles (LVRS) | Centers for Disease Control and Prevention |  | |
| EPI_ISL_311845 | EPI1205266 | A/H3 | Brazil | 2018-Jan-04 | A/Rio Grande Do Sul/27/2018 | Instituto Oswaldo Cruz FIOCRUZ - Laboratory of Respiratory Viruses and Measles (LVRS) | Centers for Disease Control and Prevention |  | |
| EPI_ISL_320147 | EPI1145927 | A/H3 | Brazil | 2017-Jul-23 | A/Parana/727/2017 | Instituto Oswaldo Cruz FIOCRUZ - Laboratory of Respiratory Viruses and Measles (LVRS) | Centers for Disease Control and Prevention |  | |
| EPI_ISL_320148 | EPI1141899 | A/H3 | Brazil | 2017-Oct-10 | A/Rio De Janeiro/927/2017 | Instituto Oswaldo Cruz FIOCRUZ - Laboratory of Respiratory Viruses and Measles (LVRS) | Centers for Disease Control and Prevention |  | |
| EPI_ISL_320149 | EPI1268806 | A/H3 | Brazil | 2018-May-21 | A/Acre/152961-IEC/2018 | National Influenza Center | Centers for Disease Control and Prevention |  | |
| EPI_ISL_320150 | EPI1266261 | A/H3 | Brazil | 2018-Apr-04 | A/Brazil/0596/2018 | Instituto Adolfo Lutz | Centers for Disease Control and Prevention |  | |
| EPI_ISL_314556 | EPI1266229 | A/H3 | Brazil | 2018-Jun-11 | A/Brazil/6356/2018 | Instituto Adolfo Lutz | Centers for Disease Control and Prevention |  | |
| EPI_ISL_328546 | EPI1266181 | A/H3 | Brazil | 2018-Mar-26 | A/Brazil/8260/2018 | Instituto Adolfo Lutz | Centers for Disease Control and Prevention |  | |
| EPI_ISL_328547 | EPI1262567 | A/H3 | Brazil | 2018-May-25 | A/Brazil/3053/2018 | Instituto Adolfo Lutz | Centers for Disease Control and Prevention |  | |
| EPI_ISL_328550 | EPI1262551 | A/H3 | Brazil | 2018-May-08 | A/Brazil/1748/2018 | Instituto Adolfo Lutz | Centers for Disease Control and Prevention |  | |
| EPI_ISL_328552 | EPI1262543 | A/H3 | Brazil | 2018-May-15 | A/Brazil/128/2018 | Instituto Adolfo Lutz | Centers for Disease Control and Prevention |  | |
| EPI_ISL_291643 | EPI1262535 | A/H3 | Brazil | 2018-Mar-06 | A/Brazil/6029/2018 | Instituto Adolfo Lutz | Centers for Disease Control and Prevention |  | |
| EPI_ISL_292522 | EPI1260893 | A/H3 | Brazil | 2018-Apr-04 | A/Brazil/106/2018 | Instituto Adolfo Lutz | Centers for Disease Control and Prevention |  | |
| EPI_ISL_300755 | EPI1198714 | A/H3 | Brazil | 2017-Aug-17 | A/Brazil/1967/2017 | Instituto Adolfo Lutz | Centers for Disease Control and Prevention |  | |
| EPI_ISL_300758 | EPI1198371 | A/H3 | Brazil | 2017-Sep-15 | A/Brazil/3559/2017 | Instituto Adolfo Lutz | Centers for Disease Control and Prevention |  | |
| EPI_ISL_303094 | EPI1198363 | A/H3 | Brazil | 2017-Sep-26 | A/Brazil/8711/2017 | Instituto Adolfo Lutz | Centers for Disease Control and Prevention |  | |
| EPI_ISL_303215 | EPI1197237 | A/H3 | Brazil | 2017-Aug-29 | A/Brazil/1445/2017 | Instituto Adolfo Lutz | Centers for Disease Control and Prevention |  | |
| EPI_ISL_303216 | EPI1185120 | A/H3 | Brazil | 2017-Oct-05 | A/Brazil/5769/2017 | Instituto Adolfo Lutz | Centers for Disease Control and Prevention |  | |
| EPI_ISL_303259 | EPI1185096 | A/H3 | Brazil | 2017-Sep-27 | A/Brazil/4558/2017 | Instituto Adolfo Lutz | Centers for Disease Control and Prevention |  | |
| EPI_ISL_304983 | EPI1279508 | A/H3 | Brazil | 2018-May-18 | A/Rio de Janeiro/640/2018 | Laboratório Central de Saúde Pública (LACEN RJ) | Instituto Oswaldo Cruz FIOCRUZ - Laboratory of Respiratory Viruses and Measles (LVRS) | NIC,FIOCRUZ | |
| EPI_ISL_304984 | EPI1279507 | A/H3 | Brazil | 2018-May-18 | A/Rio de Janeiro/639/2018 | Laboratório Central de Saúde Pública (LACEN RJ) | Instituto Oswaldo Cruz FIOCRUZ - Laboratory of Respiratory Viruses and Measles (LVRS) | NIC,FIOCRUZ | |
| EPI_ISL_313555 | EPI1279506 | A/H3 | Brazil | 2018-Apr-20 | A/Rio de Janeiro/357/2018 | Laboratório Central de Saúde Pública (LACEN RJ) | Instituto Oswaldo Cruz FIOCRUZ - Laboratory of Respiratory Viruses and Measles (LVRS) | NIC,FIOCRUZ | |
| EPI_ISL_314036 | EPI1279505 | A/H3 | Brazil | 2018-Mar-31 | A/Rio de Janeiro/176/2018 | Laboratório Central de Saúde Pública (LACEN RJ) | Instituto Oswaldo Cruz FIOCRUZ - Laboratory of Respiratory Viruses and Measles (LVRS) | NIC,FIOCRUZ | |
| EPI_ISL_316433 | EPI1279532 | A/H3 | Brazil | 2018-Jun-03 | A/Santa Catarina/595/2018 | LACEN SC | Instituto Oswaldo Cruz FIOCRUZ - Laboratory of Respiratory Viruses and Measles (LVRS) | NIC,FIOCRUZ | |
| EPI_ISL_316766 | EPI1279530 | A/H3 | Brazil | 2018-May-06 | A/Santa Catarina/395/2018 | LACEN SC | Instituto Oswaldo Cruz FIOCRUZ - Laboratory of Respiratory Viruses and Measles (LVRS) | NIC,FIOCRUZ | |
| EPI_ISL_316767 | EPI1279524 | A/H3 | Brazil | 2018-Apr-05 | A/Santa Catarina/203/2018 | LACEN SC | Instituto Oswaldo Cruz FIOCRUZ - Laboratory of Respiratory Viruses and Measles (LVRS) | NIC,FIOCRUZ | |
| EPI_ISL_316768 | EPI1279523 | A/H3 | Brazil | 2018-Mar-19 | A/Santa Catarina/162/2018 | LACEN SC | Instituto Oswaldo Cruz FIOCRUZ - Laboratory of Respiratory Viruses and Measles (LVRS) | NIC,FIOCRUZ | |
| EPI_ISL_316770 | EPI1279502 | A/H3 | Brazil | 2018-Jun-18 | A/Parana/777/2018 | Laboratório Central do Estado do Paraná - LACEN/PR | Instituto Oswaldo Cruz FIOCRUZ - Laboratory of Respiratory Viruses and Measles (LVRS) | NIC,FIOCRUZ | |
| EPI_ISL_319707 | EPI1279500 | A/H3 | Brazil | 2018-Jun-05 | A/Parana/761/2018 | Laboratório Central do Estado do Paraná - LACEN/PR | Instituto Oswaldo Cruz FIOCRUZ - Laboratory of Respiratory Viruses and Measles (LVRS) | NIC,FIOCRUZ | |
| EPI_ISL_319713 | EPI1279499 | A/H3 | Brazil | 2018-May-26 | A/Parana/757/2018 | Laboratório Central do Estado do Paraná - LACEN/PR | Instituto Oswaldo Cruz FIOCRUZ - Laboratory of Respiratory Viruses and Measles (LVRS) | NIC,FIOCRUZ | |
| EPI_ISL_319717 | EPI1279498 | A/H3 | Brazil | 2018-May-11 | A/Parana/466/2018 | Laboratório Central do Estado do Paraná - LACEN/PR | Instituto Oswaldo Cruz FIOCRUZ - Laboratory of Respiratory Viruses and Measles (LVRS) | NIC,FIOCRUZ | |
| EPI_ISL_320305 | EPI1279515 | A/H3 | Brazil | 2018-Apr-24 | A/Rio Grande do Sul/421/2018 | LACEN/RS - Laboratório Central de Saúde Pública do Rio Grande do Sul | Instituto Oswaldo Cruz FIOCRUZ - Laboratory of Respiratory Viruses and Measles (LVRS) | NIC,FIOCRUZ | |
| EPI_ISL_320306 | EPI1304998 | A/H3 | Brazil | 2018-Apr-11 | A/Amazonas/152017-IEC/2018 | Evandro Chagas Institute | Evandro Chagas Institute |  | |
| EPI_ISL_320307 | EPI1254826 | A/H3 | Brazil | 2018-Mar-19 | A/Para/151642-IEC/2018 | Evandro Chagas Institute | Evandro Chagas Institute |  | |
| EPI_ISL_320309 | EPI1091624 | A/H3 | Brazil | 2017-Sep-15 | A/Goias/387153/2017 | Instituto Adolfo Lutz | Instituto Adolfo Lutz | Santos,Katia;Silva,Daniela;Benega,Margarete;Paulino,Renato;Santos,Cecilia;Paiva,Terezinha | |
| EPI_ISL_320312 | EPI1091211 | A/H3 | Brazil | 2017-Sep-04 | A/Sao Paulo/596791/2017 | Instituto Adolfo Lutz | Instituto Adolfo Lutz | Santos,Katia;Silva,Daniela;Benega,Margarete;Paulino,Renato;Santos,Cecilia;Paiva,Terezinha | |
| EPI_ISL_320313 | EPI1091210 | A/H3 | Brazil | 2017-Aug-29 | A/Sao Paulo/593870/2017 | Instituto Adolfo Lutz | Instituto Adolfo Lutz | Santos,Katia;Silva,Daniela;Benega,Margarete;Paulino,Renato;Santos,Cecilia;Paiva,Terezinha | |
| EPI_ISL_320314 | EPI1179312 | A/H3 | Brazil | 2017-Nov-29 | A/Sao Paulo/1044167/2017 | Instituto Adolfo Lutz | Instituto Adolfo Lutz | Santos,Katia;Silva,Daniela;Benega,Margarete;Paulino,Renato;Santos,Cecilia;Paiva,Terezinha | |
| EPI_ISL_320317 | EPI1122609 | A/H3 | Brazil | 2017-Sep-25 | A/Goias/388285/2017 | Instituto Adolfo Lutz | Instituto Adolfo Lutz | Santos,Katia;Silva,Daniela;Benega,Margarete;Paulino,Renato;Santos,Cecilia;Paiva,Terezinha | |
| EPI_ISL_320319 | EPI1091625 | A/H3 | Brazil | 2017-Aug-30 | A/Sao Paulo/594145/2017 | Instituto Adolfo Lutz | Instituto Adolfo Lutz |  | |
| EPI_ISL_320320 | EPI1025276 | A/H3 | Brazil | 2017-Jun-12 | A/Sao Paulo/539114/2017 | Instituto Adolfo Lutz | Instituto Adolfo Lutz | Santos,Katia;Silva,Daniela;Benega,Margarete;Santos,Cecilia;Paiva,Terezinha | |
| EPI_ISL_321237 | EPI1023902 | A/H3 | Brazil | 2017-Jun-09 | A/Sao Paulo/537178/2017 | Instituto Adolfo Lutz | Instituto Adolfo Lutz | Santos,Katia;Silva,Daniela;Benega,Margarete;Santos,Cecilia;Paiva,Terezinha | |
| EPI_ISL_321248 | EPI1313009 | A/H3 | Brazil | 2018-Jul-16 | A/Parana/1046/2018 | Instituto Oswaldo Cruz FIOCRUZ - Laboratory of Respiratory Viruses and Measles (LVRS) | Centers for Disease Control and Prevention |  | |
| EPI_ISL_321862 | EPI1310377 | A/H3 | Brazil | 2018-Apr-25 | A/Minas Gerais/444/2018 | Instituto Oswaldo Cruz FIOCRUZ - Laboratory of Respiratory Viruses and Measles (LVRS) | Centers for Disease Control and Prevention |  | |
| EPI_ISL_321929 | EPI1274076 | A/H3 | Brazil | 2018-Jun-15 | A/Parana/817/2018 | Instituto Oswaldo Cruz FIOCRUZ - Laboratory of Respiratory Viruses and Measles (LVRS) | Centers for Disease Control and Prevention |  | |
| EPI_ISL_322966 | EPI1273988 | A/H3 | Brazil | 2018-May-18 | A/Alagoas/615/2018 | Instituto Oswaldo Cruz FIOCRUZ - Laboratory of Respiratory Viruses and Measles (LVRS) | Centers for Disease Control and Prevention |  | |
| EPI_ISL_322968 | EPI1048770 | A/H3 | Brazil | 2017-May-01 | A/Brazil/283/2017 | Instituto Oswaldo Cruz FIOCRUZ - Laboratory of Respiratory Viruses and Measles (LVRS) | Centers for Disease Control and Prevention |  | |
| EPI_ISL_329821 | EPI1268926 | A/H3 | Brazil | 2018-Apr-17 | A/Ceara/152548-IEC/2018 | National Influenza Center | Centers for Disease Control and Prevention |  | |
| EPI_ISL_329846 | EPI1268918 | A/H3 | Brazil | 2018-Apr-04 | A/Ceara/152536-IEC/2018 | National Influenza Center | Centers for Disease Control and Prevention |  | |
| EPI_ISL_329855 | EPI1268902 | A/H3 | Brazil | 2018-Apr-21 | A/Acre/152345-IEC/2018 | National Influenza Center | Centers for Disease Control and Prevention |  | |
| EPI_ISL_329857 | EPI1268878 | A/H3 | Brazil | 2018-Feb-14 | A/Amazonas/151285-IEC/2018 | National Influenza Center | Centers for Disease Control and Prevention |  | |
| EPI_ISL_329858 | EPI1268870 | A/H3 | Brazil | 2018-Apr-12 | A/Paraiba/152196-IEC/2018 | National Influenza Center | Centers for Disease Control and Prevention |  | |
| EPI_ISL_329860 | EPI1268862 | A/H3 | Brazil | 2018-Apr-03 | A/Amazonas/152016-IEC/2018 | National Influenza Center | Centers for Disease Control and Prevention |  | |
| EPI_ISL_329862 | EPI1268854 | A/H3 | Brazil | 2018-Apr-10 | A/Amapa/151957-IEC/2018 | National Influenza Center | Centers for Disease Control and Prevention |  | |
| EPI_ISL_330246 | EPI1268838 | A/H3 | Brazil | 2018-May-18 | A/Para/153301-IEC/2018 | National Influenza Center | Centers for Disease Control and Prevention |  | |
| EPI_ISL_330269 | EPI1268830 | A/H3 | Brazil | 2018-Apr-06 | A/Para/153278-IEC/2018 | National Influenza Center | Centers for Disease Control and Prevention |  | |
| EPI_ISL_330275 | EPI1268822 | A/H3 | Brazil | 2018-May-24 | A/Amazonas/153161-IEC/2018 | National Influenza Center | Centers for Disease Control and Prevention |  | |
| EPI_ISL_330279 | EPI1268814 | A/H3 | Brazil | 2018-May-05 | A/Amazonas/153154-IEC/2018 | National Influenza Center | Centers for Disease Control and Prevention |  | |
| EPI_ISL_322262 | EPI1313271 | A/H3 | Brazil | 2018-May-02 | A/Brazil/9712/2018 | Instituto Adolfo Lutz | Centers for Disease Control and Prevention |  | |
| EPI_ISL_322291 | EPI1313240 | A/H3 | Brazil | 2018-Aug-01 | A/Brazil/8425/2018 | Instituto Adolfo Lutz | Centers for Disease Control and Prevention |  | |
| EPI_ISL_322293 | EPI1313192 | A/H3 | Brazil | 2018-Jul-07 | A/Brazil/8178/2018 | Instituto Adolfo Lutz | Centers for Disease Control and Prevention |  | |
| EPI_ISL_322294 | EPI1310705 | A/H3 | Brazil | 2018-May-21 | A/Brazil/8380/2018 | Instituto Adolfo Lutz | Centers for Disease Control and Prevention |  | |
| EPI_ISL_322295 | EPI1310689 | A/H3 | Brazil | 2018-Jun-27 | A/Brazil/8415/2018 | Instituto Adolfo Lutz | Centers for Disease Control and Prevention |  | |
| EPI_ISL_299595 | EPI1310673 | A/H3 | Brazil | 2018-Jun-23 | A/Brazil/5866/2018 | Instituto Adolfo Lutz | Centers for Disease Control and Prevention |  | |
| EPI_ISL_314555 | EPI1310665 | A/H3 | Brazil | 2018-Jun-26 | A/Brazil/8341/2018 | Instituto Adolfo Lutz | Centers for Disease Control and Prevention |  | |
| EPI_ISL_328543 | EPI1310649 | A/H3 | Brazil | 2018-Jun-28 | A/Brazil/6494/2018 | Instituto Adolfo Lutz | Centers for Disease Control and Prevention |  | |
| EPI_ISL_320308 | EPI1310577 | A/H3 | Brazil | 2018-Jul-24 | A/Brazil/9555/2018 | Instituto Adolfo Lutz | Centers for Disease Control and Prevention |  | |
| EPI_ISL_320311 | EPI1282934 | A/H3 | Brazil | 2018-Jun-19 | A/Brazil/1767/2018 | Instituto Adolfo Lutz | Centers for Disease Control and Prevention |  | |
| EPI_ISL_274157 | EPI1282918 | A/H3 | Brazil | 2018-Jun-13 | A/Brazil/7947/2018 | Instituto Adolfo Lutz | Centers for Disease Control and Prevention |  | |
| EPI_ISL_274181 | EPI1277697 | A/H3 | Brazil | 2018-Jun-20 | A/Brazil/3223/2018 | Instituto Adolfo Lutz | Centers for Disease Control and Prevention |  | |
| EPI_ISL_274656 | EPI1277162 | A/H3 | Brazil | 2018-May-29 | A/Brazil/0282/2018 | Instituto Adolfo Lutz | Centers for Disease Control and Prevention |  | |
| EPI_ISL_275300 | EPI1047862 | A/H3 | Brazil | 2017-Jun-12 | A/Brazil/9114/2017 | Instituto Adolfo Lutz | Centers for Disease Control and Prevention |  | |
| EPI_ISL_275301 | EPI1045694 | A/H3 | Brazil | 2017-Jun-29 | A/Minas Gerais/626/2017 | FUNED MG | Instituto Oswaldo Cruz FIOCRUZ - Laboratory of Respiratory Viruses and Measles (LVRS) | NIC,FIOCRUZ-RJ | |
| EPI_ISL_275302 | EPI1279529 | A/H3 | Brazil | 2018-May-01 | A/Santa Catarina/337/2018 | LACEN SC | Instituto Oswaldo Cruz FIOCRUZ - Laboratory of Respiratory Viruses and Measles (LVRS) | NIC,FIOCRUZ | |
| EPI_ISL_275305 | EPI1279528 | A/H3 | Brazil | 2018-Apr-19 | A/Santa Catarina/334/2018 | LACEN SC | Instituto Oswaldo Cruz FIOCRUZ - Laboratory of Respiratory Viruses and Measles (LVRS) | NIC,FIOCRUZ | |
| EPI_ISL_275310 | EPI1279527 | A/H3 | Brazil | 2018-Apr-23 | A/Santa Catarina/333/2018 | LACEN SC | Instituto Oswaldo Cruz FIOCRUZ - Laboratory of Respiratory Viruses and Measles (LVRS) | NIC,FIOCRUZ | |
| EPI_ISL_275313 | EPI1279525 | A/H3 | Brazil | 2018-Apr-05 | A/Santa Catarina/209/2018 | LACEN SC | Instituto Oswaldo Cruz FIOCRUZ - Laboratory of Respiratory Viruses and Measles (LVRS) | NIC,FIOCRUZ | |
| EPI_ISL_275314 | EPI1045707 | A/H3 | Brazil | 2017-Jun-21 | A/Santa Catarina/452/2017 | LACEN SC | Instituto Oswaldo Cruz FIOCRUZ - Laboratory of Respiratory Viruses and Measles (LVRS) | NIC,FIOCRUZ-RJ | |
| EPI_ISL_275315 | EPI1045705 | A/H3 | Brazil | 2017-Jun-21 | A/Santa Catarina/451/2017 | LACEN SC | Instituto Oswaldo Cruz FIOCRUZ - Laboratory of Respiratory Viruses and Measles (LVRS) | NIC,FIOCRUZ-RJ | |
| EPI_ISL_299733 | EPI1045703 | A/H3 | Brazil | 2017-Jun-15 | A/Santa Catarina/449/2017 | LACEN SC | Instituto Oswaldo Cruz FIOCRUZ - Laboratory of Respiratory Viruses and Measles (LVRS) | NIC,FIOCRUZ-RJ | |
| EPI_ISL_299748 | EPI1279495 | A/H3 | Brazil | 2018-Mar-25 | A/Parana/327/2018 | Laboratório Central do Estado do Paraná - LACEN/PR | Instituto Oswaldo Cruz FIOCRUZ - Laboratory of Respiratory Viruses and Measles (LVRS) | NIC,FIOCRUZ | |
| EPI_ISL_299752 | EPI1045699 | A/H3 | Brazil | 2017-Jul-20 | A/Parana/722/2017 | Laboratório Central do Estado do Paraná - LACEN/PR | Instituto Oswaldo Cruz FIOCRUZ - Laboratory of Respiratory Viruses and Measles (LVRS) | NIC,FIOCRUZ-RJ | |
| EPI_ISL_299753 | EPI1179711 | A/H3 | Brazil | 2017-Jun-28 | A/Santa Catarina/729/2017 | LACEN/SC | Instituto Oswaldo Cruz FIOCRUZ - Laboratory of Respiratory Viruses and Measles (LVRS) | NIC, FIOCRUZ | |
| EPI_ISL_322214 | EPI1040856 | A/H3 | Brazil | 2017-May-18 | A/Rio Grande do Sul/291/2017 | LACEN/RS - Laboratório Central de Saúde Pública do Rio Grande do Sul | Instituto Oswaldo Cruz FIOCRUZ - Laboratory of Respiratory Viruses and Measles (LVRS) | National Influenza Center, FIOCRUZ, Rio de Janeiro | |
| EPI_ISL_269787 | EPI1040831 | A/H3 | Brazil | 2017-May-03 | A/Rio Grande do Sul/284/2017 | LACEN/RS - Laboratório Central de Saúde Pública do Rio Grande do Sul | Instituto Oswaldo Cruz FIOCRUZ - Laboratory of Respiratory Viruses and Measles (LVRS) | NIC,FIOCRUZ-RJ | |
| EPI_ISL_269967 | EPI1179717 | A/H3 | Brazil | 2017-Jun-01 | A/Bahia/672/2017 | Laboratório Central de Saúde Pública Professor Gonçalo Moniz, LACEN-BA | Instituto Oswaldo Cruz FIOCRUZ - Laboratory of Respiratory Viruses and Measles (LVRS) | NIC, FIOCRUZ | |
| EPI_ISL_283618 | EPI1179715 | A/H3 | Brazil | 2017-May-02 | A/Bahia/307/2017 | Laboratório Central de Saúde Pública Professor Gonçalo Moniz, LACEN-BA | Instituto Oswaldo Cruz FIOCRUZ - Laboratory of Respiratory Viruses and Measles (LVRS) | NIC, FIOCRUZ | |
| EPI_ISL_283619 | EPI1179692 | A/H3 | Brazil | 2018-Jan-11 | A/Bahia/20/2018 | Laboratório Central de Saúde Pública Professor Gonçalo Moniz, LACEN-BA | Instituto Oswaldo Cruz FIOCRUZ - Laboratory of Respiratory Viruses and Measles (LVRS) | NIC, FIOCRUZ | |
| EPI_ISL_283672 | EPI1279447 | A/H3 | Brazil | 2018-Apr-04 | A/Espirito Santo/370/2018 | LACEN/ES - Laboratório Central de Saúde Pública do Espírito Santo | Instituto Oswaldo Cruz FIOCRUZ - Laboratory of Respiratory Viruses and Measles (LVRS) | NIC,FIOCRUZ | |
| EPI_ISL_283673 | EPI1045691 | A/H3 | Brazil | 2017-Jun-03 | A/Espirito Santo/503/2017 | LACEN/ES - Laboratório Central de Saúde Pública do Espírito Santo | Instituto Oswaldo Cruz FIOCRUZ - Laboratory of Respiratory Viruses and Measles (LVRS) | NIC,FIOCRUZ-RJ | |
| EPI_ISL_288269 | EPI1045690 | A/H3 | Brazil | 2017-Jun-03 | A/Espirito Santo/502/2017 | LACEN/ES - Laboratório Central de Saúde Pública do Espírito Santo | Instituto Oswaldo Cruz FIOCRUZ - Laboratory of Respiratory Viruses and Measles (LVRS) | NIC,FIOCRUZ-RJ | |
| EPI_ISL_275741 | EPI1045689 | A/H3 | Brazil | 2017-Jun-03 | A/Espirito Santo/500/2017 | LACEN/ES - Laboratório Central de Saúde Pública do Espírito Santo | Instituto Oswaldo Cruz FIOCRUZ - Laboratory of Respiratory Viruses and Measles (LVRS) | NIC,FIOCRUZ-RJ | |
| EPI_ISL_275857 | EPI1043553 | A/H3 | Brazil | 2017-May-07 | A/Espirito Santo/358/2017 | LACEN/ES - Laboratório Central de Saúde Pública do Espírito Santo | Instituto Oswaldo Cruz FIOCRUZ - Laboratory of Respiratory Viruses and Measles (LVRS) | National Influenza Center, FIOCRUZ, Rio de Janeiro | |
| EPI_ISL_275860 | EPI1254838 | A/H3 | Brazil | 2018-Feb-16 | A/Pernambuco/151608-IEC/2018 | Evandro Chagas Institute | Evandro Chagas Institute |  | |
| EPI_ISL_275862 | EPI1122604 | A/H3 | Brazil | 2017-Sep-25 | A/Sao Paulo/610485/2017 | Instituto Adolfo Lutz | Instituto Adolfo Lutz | Santos,Katia;Silva,Daniela;Benega,Margarete;Paulino,Renato;Santos,Cecilia;Paiva,Terezinha | |
| EPI_ISL_275871 | EPI1091213 | A/H3 | Brazil | 2017-Sep-12 | A/Goias/386531/2017 | Instituto Adolfo Lutz | Instituto Adolfo Lutz | Santos,Katia;Silva,Daniela;Benega,Margarete;Paulino,Renato;Santos,Cecilia;Paiva,Terezinha | |
| EPI_ISL_275877 | EPI1274084 | A/H3 | Brazil | 2018-Jun-04 | A/Parana/762/2018 | Instituto Oswaldo Cruz FIOCRUZ - Laboratory of Respiratory Viruses and Measles (LVRS) | Centers for Disease Control and Prevention |  | |
| EPI_ISL_291642 | EPI1274004 | A/H3 | Brazil | 2018-Apr-22 | A/Bahia/563/2018 | Instituto Oswaldo Cruz FIOCRUZ - Laboratory of Respiratory Viruses and Measles (LVRS) | Centers for Disease Control and Prevention |  | |
| EPI_ISL_292523 | EPI1145943 | A/H3 | Brazil | 2017-Oct-16 | A/Santa Catarina/918/2017 | Instituto Oswaldo Cruz FIOCRUZ - Laboratory of Respiratory Viruses and Measles (LVRS) | Centers for Disease Control and Prevention |  | |
| EPI_ISL_303213 | EPI1145935 | A/H3 | Brazil | 2017-Jul-10 | A/Santa Catarina/737/2017 | Instituto Oswaldo Cruz FIOCRUZ - Laboratory of Respiratory Viruses and Measles (LVRS) | Centers for Disease Control and Prevention |  | |
| EPI_ISL_303214 | EPI1141891 | A/H3 | Brazil | 2017-Oct-17 | A/Parana/929/2017 | Instituto Oswaldo Cruz FIOCRUZ - Laboratory of Respiratory Viruses and Measles (LVRS) | Centers for Disease Control and Prevention |  | |
| EPI_ISL_303258 | EPI1048972 | A/H3 | Brazil | 2017-Jun-12 | A/Brazil/486/2017 | Instituto Oswaldo Cruz FIOCRUZ - Laboratory of Respiratory Viruses and Measles (LVRS) | Centers for Disease Control and Prevention |  | |
| EPI_ISL_314340 | EPI1048924 | A/H3 | Brazil | 2017-May-29 | A/Brazil/478/2017 | Instituto Oswaldo Cruz FIOCRUZ - Laboratory of Respiratory Viruses and Measles (LVRS) | Centers for Disease Control and Prevention |  | |
| EPI_ISL_321239 | EPI1048879 | A/H3 | Brazil | 2017-Jun-15 | A/Brazil/449/2017 | Instituto Oswaldo Cruz FIOCRUZ - Laboratory of Respiratory Viruses and Measles (LVRS) | Centers for Disease Control and Prevention |  | |
| EPI_ISL_274172 | EPI1048806 | A/H3 | Brazil | 2017-May-18 | A/Brazil/291/2017 | Instituto Oswaldo Cruz FIOCRUZ - Laboratory of Respiratory Viruses and Measles (LVRS) | Centers for Disease Control and Prevention |  | |
| EPI_ISL_275309 | EPI1048788 | A/H3 | Brazil | 2017-May-03 | A/Brazil/284/2017 | Instituto Oswaldo Cruz FIOCRUZ - Laboratory of Respiratory Viruses and Measles (LVRS) | Centers for Disease Control and Prevention |  | |
| EPI_ISL_275883 | EPI1313343 | A/H3 | Brazil | 2018-Jul-09 | A/Brazil/7331/2018 | Instituto Adolfo Lutz | Centers for Disease Control and Prevention |  | |
| EPI_ISL_275304 | EPI1313303 | A/H3 | Brazil | 2018-Jul-26 | A/Brazil/4827/2018 | Instituto Adolfo Lutz | Centers for Disease Control and Prevention |  | |
| EPI_ISL_306024 | EPI1313295 | A/H3 | Brazil | 2018-Aug-05 | A/Brazil/4139/2018 | Instituto Adolfo Lutz | Centers for Disease Control and Prevention |  | |
| EPI_ISL_306026 | EPI1313248 | A/H3 | Brazil | 2018-Jun-25 | A/Brazil/7180/2018 | Instituto Adolfo Lutz | Centers for Disease Control and Prevention |  | |
| EPI_ISL_306035 | EPI1310697 | A/H3 | Brazil | 2018-May-24 | A/Brazil/8306/2018 | Instituto Adolfo Lutz | Centers for Disease Control and Prevention |  | |
| EPI_ISL_322230 | EPI1310681 | A/H3 | Brazil | 2018-Jun-21 | A/Brazil/4623/2018 | Instituto Adolfo Lutz | Centers for Disease Control and Prevention |  | |
| EPI_ISL_322268 | EPI1277705 | A/H3 | Brazil | 2018-Jun-19 | A/Brazil/0593/2018 | Instituto Adolfo Lutz | Centers for Disease Control and Prevention |  | |
| EPI_ISL_322276 | EPI1262559 | A/H3 | Brazil | 2018-Apr-24 | A/Brazil/0750/2018 | Instituto Adolfo Lutz | Centers for Disease Control and Prevention |  | |
| EPI_ISL_322292 | EPI1253195 | A/H3 | Brazil | 2017-Aug-30 | A/Brazil/4145/2017 | Instituto Adolfo Lutz | Centers for Disease Control and Prevention |  | |
| EPI_ISL_322299 | EPI1198708 | A/H3 | Brazil | 2017-Sep-25 | A/Brazil/8285/2017 | Instituto Adolfo Lutz | Centers for Disease Control and Prevention |  | |
| EPI_ISL_322301 | EPI1198395 | A/H3 | Brazil | 2017-Sep-25 | A/Brazil/0485/2017 | Instituto Adolfo Lutz | Centers for Disease Control and Prevention |  | |
| EPI_ISL_283621 | EPI1198355 | A/H3 | Brazil | 2017-Aug-29 | A/Brazil/3870/2017 | Instituto Adolfo Lutz | Centers for Disease Control and Prevention |  | |
| EPI_ISL_288264 | EPI1198347 | A/H3 | Brazil | 2017-Sep-04 | A/Brazil/6791/2017 | Instituto Adolfo Lutz | Centers for Disease Control and Prevention |  | |
| EPI_ISL_314561 | EPI1180704 | A/H3 | Brazil | 2017-Dec-09 | A/Brazil/6531/2017 | Instituto Adolfo Lutz | Centers for Disease Control and Prevention |  | |
| EPI_ISL_292524 | EPI1279463 | A/H3 | Brazil | 2018-Apr-20 | A/Minas Gerais/441/2018 | FUNED MG | Instituto Oswaldo Cruz FIOCRUZ - Laboratory of Respiratory Viruses and Measles (LVRS) | NIC,FIOCRUZ | |
| EPI_ISL_299903 | EPI1211166 | A/H3 | Brazil | 2017-Oct-10 | A/Minas Gerais/1068/2017 | FUNED MG | Instituto Oswaldo Cruz FIOCRUZ - Laboratory of Respiratory Viruses and Measles (LVRS) | NIC,FIOCRUZ | |
| EPI_ISL_303219 | EPI1211164 | A/H3 | Brazil | 2017-Oct-09 | A/Minas Gerais/1066/2017 | FUNED MG | Instituto Oswaldo Cruz FIOCRUZ - Laboratory of Respiratory Viruses and Measles (LVRS) | NIC,FIOCRUZ | |
| EPI_ISL_316769 | EPI1045693 | A/H3 | Brazil | 2017-Jun-27 | A/Minas Gerais/625/2017 | FUNED MG | Instituto Oswaldo Cruz FIOCRUZ - Laboratory of Respiratory Viruses and Measles (LVRS) | NIC,FIOCRUZ-RJ | |
| EPI_ISL_321249 | EPI1279509 | A/H3 | Brazil | 2018-Jun-26 | A/Rio de Janeiro/828/2018 | Laboratório Central de Saúde Pública (LACEN RJ) | Instituto Oswaldo Cruz FIOCRUZ - Laboratory of Respiratory Viruses and Measles (LVRS) | NIC,FIOCRUZ | |
| EPI_ISL_321930 | EPI1211176 | A/H3 | Brazil | 2018-Jan-29 | A/Rio de Janeiro/78/2018 | Laboratório Central de Saúde Pública (LACEN RJ) | Instituto Oswaldo Cruz FIOCRUZ - Laboratory of Respiratory Viruses and Measles (LVRS) | NIC,FIOCRUZ | |
| EPI_ISL_329859 | EPI1279535 | A/H3 | Brazil | 2018-Jul-09 | A/Santa Catarina/871/2018 | LACEN SC | Instituto Oswaldo Cruz FIOCRUZ - Laboratory of Respiratory Viruses and Measles (LVRS) | NIC,FIOCRUZ | |
| EPI_ISL_329861 | EPI1279533 | A/H3 | Brazil | 2018-Jun-14 | A/Santa Catarina/668/2018 | LACEN SC | Instituto Oswaldo Cruz FIOCRUZ - Laboratory of Respiratory Viruses and Measles (LVRS) | NIC,FIOCRUZ | |
| EPI_ISL_330276 | EPI1279526 | A/H3 | Brazil | 2018-Apr-15 | A/Santa Catarina/256/2018 | LACEN SC | Instituto Oswaldo Cruz FIOCRUZ - Laboratory of Respiratory Viruses and Measles (LVRS) | NIC,FIOCRUZ | |
| EPI_ISL_330282 | EPI1279501 | A/H3 | Brazil | 2018-Jun-18 | A/Parana/776/2018 | Laboratório Central do Estado do Paraná - LACEN/PR | Instituto Oswaldo Cruz FIOCRUZ - Laboratory of Respiratory Viruses and Measles (LVRS) | NIC,FIOCRUZ | |
| EPI_ISL_330283 | EPI1045698 | A/H3 | Brazil | 2017-Jul-11 | A/Parana/719/2017 | Laboratório Central do Estado do Paraná - LACEN/PR | Instituto Oswaldo Cruz FIOCRUZ - Laboratory of Respiratory Viruses and Measles (LVRS) | NIC,FIOCRUZ-RJ | |
| EPI_ISL_330288 | EPI1040847 | A/H3 | Brazil | 2017-May-24 | A/Parana/347/2017 | Laboratório Central do Estado do Paraná - LACEN/PR | Instituto Oswaldo Cruz FIOCRUZ - Laboratory of Respiratory Viruses and Measles (LVRS) | National Influenza Center, FIOCRUZ, Rio de Janeiro | |
| EPI_ISL_273333 | EPI1267818 | B/Vic | Brazil | 2017-Dec-02 | B/Sao Paulo/667084/2017 | Instituto Adolfo Lutz | Instituto Adolfo Lutz | Santos,Katia;Silva,Daniela;Benega,Margarete;Paulino,Renato;Santos,Cecilia;Paiva,Terezinha | |
| EPI_ISL_276440 | EPI1267588 | B/Vic | Brazil | 2018-Feb-02 | B/Sao Paulo/722199/2018 | Instituto Adolfo Lutz | Instituto Adolfo Lutz | Santos,Katia;Silva,Daniela;Benega,Margarete;Paulino,Renato;Santos,Cecilia;Paiva,Terezinha | |
| EPI_ISL_281646 | EPI1238751 | B/Vic | Brazil | 2018-Feb-04 | B/Sao Paulo/712227/2018 | Instituto Adolfo Lutz | Instituto Adolfo Lutz | Santos,Katia;Silva,Daniela;Benega,Margarete;Santos,Cecilia;Paiva,Terezinha | |
| EPI_ISL_292744 | EPI1036517 | B/Vic | Brazil | 2017-May-26 | B/Sao Paulo/526128/2017 | Instituto Adolfo Lutz | Instituto Adolfo Lutz | Santos,Katia;Silva,Daniela;Benega,Margarete;Santos,Cecilia;Paiva,Terezinha | |
| EPI_ISL_296609 | EPI1247831 | B/Vic | Brazil | 2018-Feb-25 | B/Parana/151/2018 | Instituto Oswaldo Cruz FIOCRUZ - Laboratory of Respiratory Viruses and Measles (LVRS) | Centers for Disease Control and Prevention |  | |
| EPI_ISL_311498 | EPI1166968 | B/Vic | Brazil | 2017-Jul-20 | B/Santa Catarina/744/2017 | Instituto Oswaldo Cruz FIOCRUZ - Laboratory of Respiratory Viruses and Measles (LVRS) | Centers for Disease Control and Prevention |  | |
| EPI_ISL_313223 | EPI1147674 | B/Vic | Brazil | 2017-Nov-05 | B/Parana/967/2017 | Instituto Oswaldo Cruz FIOCRUZ - Laboratory of Respiratory Viruses and Measles (LVRS) | Centers for Disease Control and Prevention |  | |
| EPI_ISL_320151 | EPI1272368 | B/Vic | Brazil | 2018-May-15 | B/Amazonas/153158-IEC/2018 | National Influenza Center | Centers for Disease Control and Prevention |  | |
| EPI_ISL_320178 | EPI1268495 | B/Vic | Brazil | 2018-Apr-24 | B/Amazonas/152512-IEC/2018 | National Influenza Center | Centers for Disease Control and Prevention |  | |
| EPI_ISL_320265 | EPI1312993 | B/Vic | Brazil | 2018-Aug-10 | B/Brazil/4835/2018 | Instituto Adolfo Lutz | Centers for Disease Control and Prevention |  | |
| EPI_ISL_320923 | EPI1311274 | B/Vic | Brazil | 2018-Jun-20 | B/Brazil/2486/2018 | Instituto Adolfo Lutz | Centers for Disease Control and Prevention |  | |
| EPI_ISL_322206 | EPI1311088 | B/Vic | Brazil | 2018-Jul-25 | B/Brazil/2470/2018 | Instituto Adolfo Lutz | Centers for Disease Control and Prevention |  | |
| EPI_ISL_322224 | EPI1078748 | B/Vic | Brazil | 2017-May-26 | B/Brazil/6128/2017 | Instituto Adolfo Lutz | Centers for Disease Control and Prevention |  | |
| EPI_ISL_322239 | EPI1051878 | B/Vic | Brazil | 2017-May-25 | B/Brazil/4558/2017 | Instituto Adolfo Lutz | Centers for Disease Control and Prevention |  | |
| EPI_ISL_322241 | EPI1279457 | B/Vic | Brazil | 2018-Jan-10 | B/Minas Gerais/38/2018 | FUNED MG | Instituto Oswaldo Cruz FIOCRUZ - Laboratory of Respiratory Viruses and Measles (LVRS) | NIC,FIOCRUZ | |
| EPI_ISL_322243 | EPI1279504 | B/Vic | Brazil | 2018-Apr-03 | B/Rio de Janeiro/353/2018 | Laboratório Central de Saúde Pública (LACEN RJ) | Instituto Oswaldo Cruz FIOCRUZ - Laboratory of Respiratory Viruses and Measles (LVRS) | NIC,FIOCRUZ | |
| EPI_ISL_322244 | EPI1279503 | B/Vic | Brazil | 2018-May-01 | B/Rio de Janeiro/352/2018 | Laboratório Central de Saúde Pública (LACEN RJ) | Instituto Oswaldo Cruz FIOCRUZ - Laboratory of Respiratory Viruses and Measles (LVRS) | NIC,FIOCRUZ | |
| EPI_ISL_322245 | EPI1279516 | B/Vic | Brazil | 2018-Feb-08 | B/Santa Catarina/61/2018 | LACEN SC | Instituto Oswaldo Cruz FIOCRUZ - Laboratory of Respiratory Viruses and Measles (LVRS) | NIC,FIOCRUZ | |
| EPI_ISL_322270 | EPI1279478 | B/Vic | Brazil | 2018-May-03 | B/Parana/464/2018 | Laboratório Central do Estado do Paraná - LACEN/PR | Instituto Oswaldo Cruz FIOCRUZ - Laboratory of Respiratory Viruses and Measles (LVRS) | NIC,FIOCRUZ | |
| EPI_ISL_322271 | EPI1279477 | B/Vic | Brazil | 2018-Apr-09 | B/Parana/324/2018 | Laboratório Central do Estado do Paraná - LACEN/PR | Instituto Oswaldo Cruz FIOCRUZ - Laboratory of Respiratory Viruses and Measles (LVRS) | NIC,FIOCRUZ | |
| EPI_ISL_322282 | EPI1279476 | B/Vic | Brazil | 2018-Apr-24 | B/Parana/316/2018 | Laboratório Central do Estado do Paraná - LACEN/PR | Instituto Oswaldo Cruz FIOCRUZ - Laboratory of Respiratory Viruses and Measles (LVRS) | NIC,FIOCRUZ | |
| EPI_ISL_329911 | EPI1279474 | B/Vic | Brazil | 2018-Mar-06 | B/Parana/152/2018 | Laboratório Central do Estado do Paraná - LACEN/PR | Instituto Oswaldo Cruz FIOCRUZ - Laboratory of Respiratory Viruses and Measles (LVRS) | NIC,FIOCRUZ | |
| EPI_ISL_329935 | EPI1279472 | B/Vic | Brazil | 2018-Feb-20 | B/Parana/95/2018 | Laboratório Central do Estado do Paraná - LACEN/PR | Instituto Oswaldo Cruz FIOCRUZ - Laboratory of Respiratory Viruses and Measles (LVRS) | NIC,FIOCRUZ | |
| EPI_ISL_330244 | EPI1279439 | B/Vic | Brazil | 2018-Jan-03 | B/Espirito Santo/50/2018 | LACEN/ES - Laboratório Central de Saúde Pública do Espírito Santo | Instituto Oswaldo Cruz FIOCRUZ - Laboratory of Respiratory Viruses and Measles (LVRS) | NIC,FIOCRUZ | |
| EPI_ISL_267072 | EPI1123263 | B/Yam | Brazil | 2017-Sep-28 | B/Sao Paulo/614261/2017 | Instituto Adolfo Lutz | Instituto Adolfo Lutz | Santos,Katia;Silva,Daniela;Benega,Margarete;Paulino,Renato;Santos,Cecilia;Paiva,Terezinha | |
| EPI_ISL_269970 | EPI1123262 | B/Yam | Brazil | 2017-Sep-21 | B/Sao Paulo/608134/2017 | Instituto Adolfo Lutz | Instituto Adolfo Lutz | Santos,Katia;Silva,Daniela;Benega,Margarete;Paulino,Renato;Santos,Cecilia;Paiva,Terezinha | |
| EPI_ISL_270353 | EPI1122720 | B/Yam | Brazil | 2017-Oct-03 | B/Mato Grosso do Sul/493889/2017 | Instituto Adolfo Lutz | Instituto Adolfo Lutz | Santos,Katia;Silva,Daniela;Benega,Margarete;Paulino,Renato;Santos,Cecilia;Paiva,Terezinha | |
| EPI_ISL_270354 | EPI1122719 | B/Yam | Brazil | 2017-Oct-05 | B/Sao Paulo/973018/2017 | Instituto Adolfo Lutz | Instituto Adolfo Lutz | Santos,Katia;Silva,Daniela;Benega,Margarete;Paulino,Renato;Santos,Cecilia;Paiva,Terezinha | |
| EPI_ISL_270355 | EPI1122718 | B/Yam | Brazil | 2017-Oct-03 | B/Sao Paulo/617506/2017 | Instituto Adolfo Lutz | Instituto Adolfo Lutz | Santos,Katia;Silva,Daniela;Benega,Margarete;Paulino,Renato;Santos,Cecilia;Paiva,Terezinha | |
| EPI_ISL_273331 | EPI1091634 | B/Yam | Brazil | 2017-Sep-11 | B/Sao Paulo/599791/2017 | Instituto Adolfo Lutz | Instituto Adolfo Lutz | Santos,Katia;Silva,Daniela;Benega,Margarete;Paulino,Renato;Santos,Cecilia;Paiva,Terezinha | |
| EPI_ISL_273332 | EPI1091633 | B/Yam | Brazil | 2017-Sep-11 | B/Sao Paulo/599763/2017 | Instituto Adolfo Lutz | Instituto Adolfo Lutz | Santos,Katia;Silva,Daniela;Benega,Margarete;Paulino,Renato;Santos,Cecilia;Paiva,Terezinha | |
| EPI_ISL_273335 | EPI1091632 | B/Yam | Brazil | 2017-Aug-14 | B/Goias/381978/2017 | Instituto Adolfo Lutz | Instituto Adolfo Lutz | Santos,Katia;Silva,Daniela;Benega,Margarete;Paulino,Renato;Santos,Cecilia;Paiva,Terezinha | |
| EPI_ISL_276439 | EPI1091631 | B/Yam | Brazil | 2017-Aug-09 | B/Mato Grosso do Sul/487340/2017 | Instituto Adolfo Lutz | Instituto Adolfo Lutz | Santos,Katia;Silva,Daniela;Benega,Margarete;Paulino,Renato;Santos,Cecilia;Paiva,Terezinha | |
| EPI_ISL_276441 | EPI1091630 | B/Yam | Brazil | 2017-Aug-22 | B/Goias/383250/2017 | Instituto Adolfo Lutz | Instituto Adolfo Lutz | Santos,Katia;Silva,Daniela;Benega,Margarete;Paulino,Renato;Santos,Cecilia;Paiva,Terezinha | |
| EPI_ISL_276442 | EPI1091629 | B/Yam | Brazil | 2017-Sep-14 | B/Goias/386852/2017 | Instituto Adolfo Lutz | Instituto Adolfo Lutz | Santos,Katia;Silva,Daniela;Benega,Margarete;Paulino,Renato;Santos,Cecilia;Paiva,Terezinha | |
| EPI_ISL_276443 | EPI1036519 | B/Yam | Brazil | 2017-Jul-07 | B/Mato Grosso/557440/2017 | Instituto Adolfo Lutz | Instituto Adolfo Lutz | Santos,Katia;Silva,Daniela;Benega,Margarete;Santos,Cecilia;Paiva,Terezinha | |
| EPI_ISL_276512 | EPI1036516 | B/Yam | Brazil | 2017-Jun-30 | B/Sao Paulo/559889/2017 | Instituto Adolfo Lutz | Instituto Adolfo Lutz | Santos,Katia;Silva,Daniela;Benega,Margarete;Santos,Cecilia;Paiva,Terezinha | |
| EPI_ISL_276513 | EPI1036515 | B/Yam | Brazil | 2017-Jun-23 | B/Sao Paulo/547054/2017 | Instituto Adolfo Lutz | Instituto Adolfo Lutz | Santos,Katia;Silva,Daniela;Benega,Margarete;Santos,Cecilia;Paiva,Terezinha | |
| EPI_ISL_276514 | EPI1027976 | B/Yam | Brazil | 2017-Jun-18 | B/Sao Paulo/540436/2017 | Instituto Adolfo Lutz | Instituto Adolfo Lutz | Santos,Katia;Silva,Daniela;Benega,Margarete;Santos,Cecilia;Paiva,Terezinha | |
| EPI_ISL_276516 | EPI1027968 | B/Yam | Brazil | 2017-Jun-13 | B/Sao Paulo/539111/2017 | Instituto Adolfo Lutz | Instituto Adolfo Lutz | Santos,Katia;Silva,Daniela;Benega,Margarete;Santos,Cecilia;Paiva,Terezinha | |
| EPI_ISL_276517 | EPI1027956 | B/Yam | Brazil | 2017-Jun-13 | B/Sao Paulo/539090/2017 | Instituto Adolfo Lutz | Instituto Adolfo Lutz | Santos,Katia;Silva,Daniela;Benega,Margarete;Santos,Cecilia;Paiva,Terezinha | |
| EPI_ISL_276518 | EPI1025302 | B/Yam | Brazil | 2017-Jun-09 | B/Sao Paulo/537807/2017 | Instituto Adolfo Lutz | Instituto Adolfo Lutz | Santos,Katia;Silva,Daniela;Benega,Margarete;Santos,Cecilia;Paiva,Terezinha | |
| EPI_ISL_276519 | EPI1010367 | B/Yam | Brazil | 2017-May-04 | B/Sao Paulo/508835/2017 | Instituto Adolfo Lutz | Instituto Adolfo Lutz | Santos,Katia;Silva,Daniela;Benega,Margarete;Santos,Cecilia;Paiva,Terezinha | |
| EPI_ISL_276520 | EPI1164665 | B/Yam | Brazil | 2017-Oct-30 | B/Parana/969/2017 | Instituto Oswaldo Cruz FIOCRUZ - Laboratory of Respiratory Viruses and Measles (LVRS) | Centers for Disease Control and Prevention |  | |
| EPI_ISL_276546 | EPI1164656 | B/Yam | Brazil | 2017-Nov-14 | B/Bahia/975/2017 | Instituto Oswaldo Cruz FIOCRUZ - Laboratory of Respiratory Viruses and Measles (LVRS) | Centers for Disease Control and Prevention |  | |
| EPI_ISL_276547 | EPI1164648 | B/Yam | Brazil | 2017-Nov-09 | B/Bahia/979/2017 | Instituto Oswaldo Cruz FIOCRUZ - Laboratory of Respiratory Viruses and Measles (LVRS) | Centers for Disease Control and Prevention |  | |
| EPI_ISL_276548 | EPI1147764 | B/Yam | Brazil | 2017-Jul-10 | B/Parana/715/2017 | Instituto Oswaldo Cruz FIOCRUZ - Laboratory of Respiratory Viruses and Measles (LVRS) | Centers for Disease Control and Prevention |  | |
| EPI_ISL_276549 | EPI1147759 | B/Yam | Brazil | 2017-Aug-17 | B/Bahia/863/2017 | Instituto Oswaldo Cruz FIOCRUZ - Laboratory of Respiratory Viruses and Measles (LVRS) | Centers for Disease Control and Prevention |  | |
| EPI_ISL_282027 | EPI1147751 | B/Yam | Brazil | 2017-Jul-28 | B/Bahia/868/2017 | Instituto Oswaldo Cruz FIOCRUZ - Laboratory of Respiratory Viruses and Measles (LVRS) | Centers for Disease Control and Prevention |  | |
| EPI_ISL_282028 | EPI1147743 | B/Yam | Brazil | 2017-Oct-06 | B/Santa Catarina/914/2017 | Instituto Oswaldo Cruz FIOCRUZ - Laboratory of Respiratory Viruses and Measles (LVRS) | Centers for Disease Control and Prevention |  | |
| EPI_ISL_282029 | EPI1147735 | B/Yam | Brazil | 2017-Oct-19 | B/Santa Catarina/920/2017 | Instituto Oswaldo Cruz FIOCRUZ - Laboratory of Respiratory Viruses and Measles (LVRS) | Centers for Disease Control and Prevention |  | |
| EPI_ISL_282030 | EPI1147727 | B/Yam | Brazil | 2017-Oct-25 | B/Santa Catarina/923/2017 | Instituto Oswaldo Cruz FIOCRUZ - Laboratory of Respiratory Viruses and Measles (LVRS) | Centers for Disease Control and Prevention |  | |
| EPI_ISL_283677 | EPI1147698 | B/Yam | Brazil | 2017-Sep-24 | B/Rio Grande Do Sul/950/2017 | Instituto Oswaldo Cruz FIOCRUZ - Laboratory of Respiratory Viruses and Measles (LVRS) | Centers for Disease Control and Prevention |  | |
| EPI_ISL_283678 | EPI1147690 | B/Yam | Brazil | 2017-Nov-02 | B/Santa Catarina/959/2017 | Instituto Oswaldo Cruz FIOCRUZ - Laboratory of Respiratory Viruses and Measles (LVRS) | Centers for Disease Control and Prevention |  | |
| EPI_ISL_283679 | EPI1147682 | B/Yam | Brazil | 2017-Oct-25 | B/Parana/966/2017 | Instituto Oswaldo Cruz FIOCRUZ - Laboratory of Respiratory Viruses and Measles (LVRS) | Centers for Disease Control and Prevention |  | |
| EPI_ISL_283680 | EPI1147666 | B/Yam | Brazil | 2017-Nov-21 | B/Parana/968/2017 | Instituto Oswaldo Cruz FIOCRUZ - Laboratory of Respiratory Viruses and Measles (LVRS) | Centers for Disease Control and Prevention |  | |
| EPI_ISL_283681 | EPI1141408 | B/Yam | Brazil | 2017-Oct-24 | B/Parana/930/2017 | Instituto Oswaldo Cruz FIOCRUZ - Laboratory of Respiratory Viruses and Measles (LVRS) | Centers for Disease Control and Prevention |  | |
| EPI_ISL_283682 | EPI1141400 | B/Yam | Brazil | 2017-Oct-18 | B/Espirito Santo/935/2017 | Instituto Oswaldo Cruz FIOCRUZ - Laboratory of Respiratory Viruses and Measles (LVRS) | Centers for Disease Control and Prevention |  | |
| EPI_ISL_288287 | EPI1141392 | B/Yam | Brazil | 2017-Oct-16 | B/Parana/933/2017 | Instituto Oswaldo Cruz FIOCRUZ - Laboratory of Respiratory Viruses and Measles (LVRS) | Centers for Disease Control and Prevention |  | |
| EPI_ISL_288288 | EPI1141384 | B/Yam | Brazil | 2017-Oct-08 | B/Espirito Santo/934/2017 | Instituto Oswaldo Cruz FIOCRUZ - Laboratory of Respiratory Viruses and Measles (LVRS) | Centers for Disease Control and Prevention |  | |
| EPI_ISL_288289 | EPI1141376 | B/Yam | Brazil | 2017-Oct-02 | B/Rio Grande Do Sul/949/2017 | Instituto Oswaldo Cruz FIOCRUZ - Laboratory of Respiratory Viruses and Measles (LVRS) | Centers for Disease Control and Prevention |  | |
| EPI_ISL_288413 | EPI1052728 | B/Yam | Brazil | 2017-Jun-27 | B/Brazil/520/2017 | Instituto Oswaldo Cruz FIOCRUZ - Laboratory of Respiratory Viruses and Measles (LVRS) | Centers for Disease Control and Prevention |  | |
| EPI_ISL_288414 | EPI1052720 | B/Yam | Brazil | 2017-Jun-12 | B/Brazil/485/2017 | Instituto Oswaldo Cruz FIOCRUZ - Laboratory of Respiratory Viruses and Measles (LVRS) | Centers for Disease Control and Prevention |  | |
| EPI_ISL_291576 | EPI1052712 | B/Yam | Brazil | 2017-May-12 | B/Brazil/359/2017 | Instituto Oswaldo Cruz FIOCRUZ - Laboratory of Respiratory Viruses and Measles (LVRS) | Centers for Disease Control and Prevention |  | |
| EPI_ISL_291577 | EPI1052704 | B/Yam | Brazil | 2017-May-23 | B/Brazil/346/2017 | Instituto Oswaldo Cruz FIOCRUZ - Laboratory of Respiratory Viruses and Measles (LVRS) | Centers for Disease Control and Prevention |  | |
| EPI_ISL_291578 | EPI1052500 | B/Yam | Brazil | 2017-Jun-26 | B/Pernambuco/148585-IEC/2017 | National Influenza Center | Centers for Disease Control and Prevention |  | |
| EPI_ISL_291579 | EPI1052492 | B/Yam | Brazil | 2017-Jun-14 | B/Paraiba/148175-IEC/2017 | National Influenza Center | Centers for Disease Control and Prevention |  | |
| EPI_ISL_291580 | EPI1052484 | B/Yam | Brazil | 2017-May-31 | B/Amapa/148142-IEC/2017 | National Influenza Center | Centers for Disease Control and Prevention |  | |
| EPI_ISL_292743 | EPI1052476 | B/Yam | Brazil | 2017-Jun-19 | B/Roraima/148140-IEC/2017 | National Influenza Center | Centers for Disease Control and Prevention |  | |
| EPI_ISL_292745 | EPI1052468 | B/Yam | Brazil | 2017-May-23 | B/Roraima/148111-IEC/2017 | National Influenza Center | Centers for Disease Control and Prevention |  | |
| EPI_ISL_292746 | EPI1052452 | B/Yam | Brazil | 2017-May-12 | B/Ceara/147634-IEC/2017 | National Influenza Center | Centers for Disease Control and Prevention |  | |
| EPI_ISL_292747 | EPI1052444 | B/Yam | Brazil | 2017-May-04 | B/Amapa/147531-IEC/2017 | National Influenza Center | Centers for Disease Control and Prevention |  | |
| EPI_ISL_292751 | EPI1052436 | B/Yam | Brazil | 2017-May-09 | B/Amazonas/147471-IEC/2017 | National Influenza Center | Centers for Disease Control and Prevention |  | |
| EPI_ISL_292752 | EPI1186787 | B/Yam | Brazil | 2017-Nov-09 | B/Brazil/9763/2017 | Instituto Adolfo Lutz | Centers for Disease Control and Prevention |  | |
| EPI_ISL_292753 | EPI1186772 | B/Yam | Brazil | 2017-Sep-28 | B/Brazil/4261/2017 | Instituto Adolfo Lutz | Centers for Disease Control and Prevention |  | |
| EPI_ISL_292754 | EPI1186748 | B/Yam | Brazil | 2017-Oct-05 | B/Brazil/3018/2017 | Instituto Adolfo Lutz | Centers for Disease Control and Prevention |  | |
| EPI_ISL_292755 | EPI1186415 | B/Yam | Brazil | 2017-Oct-03 | B/Brazil/3889/2017 | Instituto Adolfo Lutz | Centers for Disease Control and Prevention |  | |
| EPI_ISL_292756 | EPI1081470 | B/Yam | Brazil | 2017-Jun-30 | B/Brazil/9889/2017 | Instituto Adolfo Lutz | Centers for Disease Control and Prevention |  | |
| EPI_ISL_296253 | EPI1081462 | B/Yam | Brazil | 2017-Jul-07 | B/Brazil/7440/2017 | Instituto Adolfo Lutz | Centers for Disease Control and Prevention |  | |
| EPI_ISL_296254 | EPI1081454 | B/Yam | Brazil | 2017-Jun-27 | B/Brazil/7054/2017 | Instituto Adolfo Lutz | Centers for Disease Control and Prevention |  | |
| EPI_ISL_296256 | EPI1081446 | B/Yam | Brazil | 2017-Jun-24 | B/Brazil/3676/2017 | Instituto Adolfo Lutz | Centers for Disease Control and Prevention |  | |
| EPI_ISL_300926 | EPI1051901 | B/Yam | Brazil | 2017-Jun-18 | B/Brazil/0436/2017 | Instituto Adolfo Lutz | Centers for Disease Control and Prevention |  | |
| EPI_ISL_300969 | EPI1051894 | B/Yam | Brazil | 2017-Jun-13 | B/Brazil/9111/2017 | Instituto Adolfo Lutz | Centers for Disease Control and Prevention |  | |
| EPI_ISL_300972 | EPI1051886 | B/Yam | Brazil | 2017-Jun-13 | B/Brazil/9090/2017 | Instituto Adolfo Lutz | Centers for Disease Control and Prevention |  | |
| EPI_ISL_300974 | EPI1051870 | B/Yam | Brazil | 2017-May-04 | B/Brazil/8835/2017 | Instituto Adolfo Lutz | Centers for Disease Control and Prevention |  | |
| EPI_ISL_301118 | EPI1305052 | B/Yam | Brazil | 2017-Aug-17 | A/Roraima/149028-IEC/2017 | Evandro Chagas Institute | Evandro Chagas Institute |  | |
| EPI_ISL_301119 | EPI1305050 | B/Yam | Brazil | 2017-Jul-25 | A/Pernambuco/148980-IEC/2017 | Evandro Chagas Institute | Evandro Chagas Institute |  | |
| EPI_ISL_301120 | EPI1305048 | B/Yam | Brazil | 2017-Jul-03 | A/Roraima/148605-IEC/2017 | Evandro Chagas Institute | Evandro Chagas Institute |  | |
| EPI_ISL_301513 | EPI1305046 | B/Yam | Brazil | 2017-Jun-27 | A/Roraima/148604-IEC/2017 | Evandro Chagas Institute | Evandro Chagas Institute |  | |
| EPI_ISL_301514 | EPI1305044 | B/Yam | Brazil | 2017-Jun-27 | A/Pernambuco/148586-IEC/2017 | Evandro Chagas Institute | Evandro Chagas Institute |  | |
| EPI_ISL_301515 | EPI1305040 | B/Yam | Brazil | 2017-May-24 | A/Paraiba/147781-IEC/2017 | Evandro Chagas Institute | Evandro Chagas Institute |  | |
| EPI_ISL_303035 | EPI1305032 | B/Yam | Brazil | 2018-Feb-08 | B/Acre/151488-IEC/2018 | Evandro Chagas Institute | Evandro Chagas Institute |  | |
| EPI_ISL_305103 | EPI1254908 | B/Yam | Brazil | 2018-Jan-18 | B/Acre/151007-IEC/2018 | Evandro Chagas Institute | Evandro Chagas Institute |  | |
| EPI_ISL_305104 | EPI1254882 | B/Yam | Brazil | 2017-Aug-17 | B/Roraima/149028-IEC/2017 | Evandro Chagas Institute | Evandro Chagas Institute |  | |
| EPI_ISL_305188 | EPI1254880 | B/Yam | Brazil | 2017-Jul-03 | B/Roraima/148605-IEC/2017 | Evandro Chagas Institute | Evandro Chagas Institute |  | |
| EPI_ISL_305889 | EPI1254878 | B/Yam | Brazil | 2017-Jun-27 | B/Roraima/148604-IEC/2017 | Evandro Chagas Institute | Evandro Chagas Institute |  | |
| EPI_ISL_312004 | EPI1254876 | B/Yam | Brazil | 2017-Jul-25 | B/Pernambuco/148980-IEC/2017 | Evandro Chagas Institute | Evandro Chagas Institute |  | |
| EPI_ISL_313193 | EPI1254874 | B/Yam | Brazil | 2017-Jun-27 | B/Pernambuco/148586-IEC/2017 | Evandro Chagas Institute | Evandro Chagas Institute |  | |
| EPI_ISL_314566 | EPI1254872 | B/Yam | Brazil | 2017-May-16 | B/Pernambuco/148409-IEC/2017 | Evandro Chagas Institute | Evandro Chagas Institute |  | |
| EPI_ISL_314568 | EPI1254870 | B/Yam | Brazil | 2018-Apr-21 | B/Paraiba/152265-IEC/2018 | Evandro Chagas Institute | Evandro Chagas Institute |  | |
| EPI_ISL_314569 | EPI1254866 | B/Yam | Brazil | 2017-May-24 | B/Paraiba/147781-IEC/2017 | Evandro Chagas Institute | Evandro Chagas Institute |  | |
| EPI_ISL_314570 | EPI1254864 | B/Yam | Brazil | 2018-Apr-16 | B/Acre/152148-IEC/2018 | Evandro Chagas Institute | Evandro Chagas Institute |  | |
| EPI_ISL_314572 | EPI1254860 | B/Yam | Brazil | 2018-Apr-12 | B/Acre/152029-IEC/2018 | Evandro Chagas Institute | Evandro Chagas Institute |  | |
| EPI_ISL_314574 | EPI1254856 | B/Yam | Brazil | 2018-Mar-13 | B/Acre/151914-IEC/2018 | Evandro Chagas Institute | Evandro Chagas Institute |  | |
| EPI_ISL_314575 | EPI1254854 | B/Yam | Brazil | 2018-Mar-05 | B/Acre/151802-IEC/2018 | Evandro Chagas Institute | Evandro Chagas Institute |  | |
| EPI_ISL_314577 | EPI1254852 | B/Yam | Brazil | 2018-Feb-20 | B/Acre/151794-IEC/2018 | Evandro Chagas Institute | Evandro Chagas Institute |  | |
| EPI_ISL_314578 | EPI1254848 | B/Yam | Brazil | 2018-Feb-08 | B/Acre/151488-IEC/2018 | Evandro Chagas Institute | Evandro Chagas Institute |  | |
| EPI_ISL_314579 | EPI1269604 | B/Yam | Brazil | 2018-Apr-05 | B/Sao Paulo/1208952/2018 | Instituto Adolfo Lutz | Instituto Adolfo Lutz | Santos,Katia;Silva,Daniela;Benega,Margarete;Paulino,Renato;Santos,Cecilia;Paiva,Terezinha | |
| EPI_ISL_314580 | EPI1267820 | B/Yam | Brazil | 2018-Jan-16 | B/Sao Paulo/694179/2018 | Instituto Adolfo Lutz | Instituto Adolfo Lutz | Santos,Katia;Silva,Daniela;Benega,Margarete;Paulino,Renato;Santos,Cecilia;Paiva,Terezinha | |
| EPI_ISL_314581 | EPI1241860 | B/Yam | Brazil | 2018-Feb-13 | B/Distrito Federal/756370/2018 | Instituto Adolfo Lutz | Instituto Adolfo Lutz | Santos,Katia;Silva,Daniela;Benega,Margarete;Santos,Cecilia;Paiva,Terezinha | |
| EPI_ISL_314582 | EPI1189260 | B/Yam | Brazil | 2017-Nov-01 | B/Distrito Federal/707422/2017 | Instituto Adolfo Lutz | Instituto Adolfo Lutz | Santos,Katia;Silva,Daniela;Benega,Margarete;Paulino,Renato;Santos,Cecilia;Paiva,Terezinha | |
| EPI_ISL_314583 | EPI1189259 | B/Yam | Brazil | 2018-Jan-17 | B/Sao Paulo/695809/2018 | Instituto Adolfo Lutz | Instituto Adolfo Lutz | Santos,Katia;Silva,Daniela;Benega,Margarete;Paulino,Renato;Santos,Cecilia;Paiva,Terezinha | |
| EPI_ISL_314587 | EPI1189258 | B/Yam | Brazil | 2018-Jan-02 | B/Mato Grosso do Sul/504722/2018 | Instituto Adolfo Lutz | Instituto Adolfo Lutz | Santos,Katia;Silva,Daniela;Benega,Margarete;Paulino,Renato;Santos,Cecilia;Paiva,Terezinha | |
| EPI_ISL_320179 | EPI1187784 | B/Yam | Brazil | 2018-Jan-01 | B/Sao Paulo/684669/2018 | Instituto Adolfo Lutz | Instituto Adolfo Lutz | Santos,Katia;Silva,Daniela;Benega,Margarete;Paulino,Renato;Santos,Cecilia;Paiva,Terezinha | |
| EPI_ISL_320473 | EPI1187783 | B/Yam | Brazil | 2017-Dec-01 | B/Goias/841915/2017 | Instituto Adolfo Lutz | Instituto Adolfo Lutz | Santos,Katia;Silva,Daniela;Benega,Margarete;Paulino,Renato;Santos,Cecilia;Paiva,Terezinha | |
| EPI_ISL_320889 | EPI1187782 | B/Yam | Brazil | 2017-Nov-01 | B/Sao Paulo/642781/2017 | Instituto Adolfo Lutz | Instituto Adolfo Lutz | Santos,Katia;Silva,Daniela;Benega,Margarete;Paulino,Renato;Santos,Cecilia;Paiva,Terezinha | |
| EPI_ISL_320895 | EPI1273570 | B/Yam | Brazil | 2018-Apr-11 | B/Parana/323/2018 | Instituto Oswaldo Cruz FIOCRUZ - Laboratory of Respiratory Viruses and Measles (LVRS) | Centers for Disease Control and Prevention |  | |
| EPI_ISL_320896 | EPI1247589 | B/Yam | Brazil | 2018-Mar-12 | B/Espirto Santo/139/2018 | Instituto Oswaldo Cruz FIOCRUZ - Laboratory of Respiratory Viruses and Measles (LVRS) | Centers for Disease Control and Prevention |  | |
| EPI_ISL_320921 | EPI1206225 | B/Yam | Brazil | 2018-Jan-04 | B/Bahia/17/2018 | Instituto Oswaldo Cruz FIOCRUZ - Laboratory of Respiratory Viruses and Measles (LVRS) | Centers for Disease Control and Prevention |  | |
| EPI_ISL_320932 | EPI1206217 | B/Yam | Brazil | 2018-Jan-23 | B/Minas Gerais/40/2018 | Instituto Oswaldo Cruz FIOCRUZ - Laboratory of Respiratory Viruses and Measles (LVRS) | Centers for Disease Control and Prevention |  | |
| EPI_ISL_321184 | EPI1272434 | B/Yam | Brazil | 2018-May-18 | B/Paraiba/152890-IEC/2018 | National Influenza Center | Centers for Disease Control and Prevention |  | |
| EPI_ISL_322195 | EPI1272352 | B/Yam | Brazil | 2018-May-02 | B/Para/153564-IEC/2018 | National Influenza Center | Centers for Disease Control and Prevention |  | |
| EPI_ISL_322196 | EPI1272159 | B/Yam | Brazil | 2018-May-18 | B/Maranhao/152704-IEC/2018 | National Influenza Center | Centers for Disease Control and Prevention |  | |
| EPI_ISL_322197 | EPI1272152 | B/Yam | Brazil | 2018-Apr-20 | B/Ceara/152552-IEC/2018 | National Influenza Center | Centers for Disease Control and Prevention |  | |
| EPI_ISL_322204 | EPI1272107 | B/Yam | Brazil | 2018-Apr-26 | B/Acre/152344-IEC/2018 | National Influenza Center | Centers for Disease Control and Prevention |  | |
| EPI_ISL_322205 | EPI1210595 | B/Yam | Brazil | 2017-Oct-03 | B/Brazil/7506/2017 | Instituto Adolfo Lutz | Centers for Disease Control and Prevention |  | |
| EPI_ISL_322207 | EPI1206891 | B/Yam | Brazil | 2017-Sep-21 | B/Brazil/8134/2017 | Instituto Adolfo Lutz | Centers for Disease Control and Prevention |  | |
| EPI_ISL_322208 | EPI1196786 | B/Yam | Brazil | 2017-Sep-23 | B/Brazil/0459/2017 | Instituto Adolfo Lutz | Centers for Disease Control and Prevention |  | |
| EPI_ISL_322209 | EPI1279518 | B/Yam | Brazil | 2018-May-21 | B/Santa Catarina/486/2018 | LACEN SC | Instituto Oswaldo Cruz FIOCRUZ - Laboratory of Respiratory Viruses and Measles (LVRS) | NIC,FIOCRUZ | |
| EPI_ISL_322210 | EPI1279517 | B/Yam | Brazil | 2018-Apr-21 | B/Santa Catarina/335/2018 | LACEN SC | Instituto Oswaldo Cruz FIOCRUZ - Laboratory of Respiratory Viruses and Measles (LVRS) | NIC,FIOCRUZ | |
| EPI_ISL_322236 | EPI1279475 | B/Yam | Brazil | 2018-Mar-24 | B/Parana/159/2018 | Laboratório Central do Estado do Paraná - LACEN/PR | Instituto Oswaldo Cruz FIOCRUZ - Laboratory of Respiratory Viruses and Measles (LVRS) | NIC,FIOCRUZ | |
| EPI_ISL_322237 | EPI1279473 | B/Yam | Brazil | 2018-Feb-28 | B/Parana/150/2018 | Laboratório Central do Estado do Paraná - LACEN/PR | Instituto Oswaldo Cruz FIOCRUZ - Laboratory of Respiratory Viruses and Measles (LVRS) | NIC,FIOCRUZ | |
| EPI_ISL_322238 | EPI1279471 | B/Yam | Brazil | 2018-Jan-19 | B/Parana/60/2018 | Laboratório Central do Estado do Paraná - LACEN/PR | Instituto Oswaldo Cruz FIOCRUZ - Laboratory of Respiratory Viruses and Measles (LVRS) | NIC,FIOCRUZ | |
| EPI_ISL_322240 | EPI1279470 | B/Yam | Brazil | 2018-Jan-24 | B/Parana/58/2018 | Laboratório Central do Estado do Paraná - LACEN/PR | Instituto Oswaldo Cruz FIOCRUZ - Laboratory of Respiratory Viruses and Measles (LVRS) | NIC,FIOCRUZ | |
| EPI_ISL_322242 | EPI1279469 | B/Yam | Brazil | 2018-Jan-23 | B/Parana/55/2018 | Laboratório Central do Estado do Paraná - LACEN/PR | Instituto Oswaldo Cruz FIOCRUZ - Laboratory of Respiratory Viruses and Measles (LVRS) | NIC,FIOCRUZ | |
| EPI_ISL_322277 | EPI1279512 | B/Yam | Brazil | 2018-May-08 | B/Rio Grande do Sul/505/2018 | LACEN/RS - Laboratório Central de Saúde Pública do Rio Grande do Sul | Instituto Oswaldo Cruz FIOCRUZ - Laboratory of Respiratory Viruses and Measles (LVRS) | NIC,FIOCRUZ | |
| EPI_ISL_322278 | EPI1279511 | B/Yam | Brazil | 2018-Apr-30 | B/Rio Grande do Sul/418/2018 | LACEN/RS - Laboratório Central de Saúde Pública do Rio Grande do Sul | Instituto Oswaldo Cruz FIOCRUZ - Laboratory of Respiratory Viruses and Measles (LVRS) | NIC,FIOCRUZ | |
| EPI_ISL_322283 | EPI1279429 | B/Yam | Brazil | 2018-Jun-05 | B/Bahia/559/2018 | Laboratório Central de Saúde Pública Professor Gonçalo Moniz, LACEN-BA | Instituto Oswaldo Cruz FIOCRUZ - Laboratory of Respiratory Viruses and Measles (LVRS) | NIC,FIOCRUZ | |
| EPI_ISL_322284 | EPI1279428 | B/Yam | Brazil | 2018-Mar-02 | B/Bahia/115/2018 | Laboratório Central de Saúde Pública Professor Gonçalo Moniz, LACEN-BA | Instituto Oswaldo Cruz FIOCRUZ - Laboratory of Respiratory Viruses and Measles (LVRS) | NIC,FIOCRUZ | |
| EPI_ISL_328561 | EPI1279427 | B/Yam | Brazil | 2018-Jan-11 | B/Bahia/19/2018 | Laboratório Central de Saúde Pública Professor Gonçalo Moniz, LACEN-BA | Instituto Oswaldo Cruz FIOCRUZ - Laboratory of Respiratory Viruses and Measles (LVRS) | NIC,FIOCRUZ | |
| EPI_ISL_328567 | EPI1279443 | B/Yam | Brazil | 2018-May-01 | B/Espirito Santo/383/2018 | LACEN/ES - Laboratório Central de Saúde Pública do Espírito Santo | Instituto Oswaldo Cruz FIOCRUZ - Laboratory of Respiratory Viruses and Measles (LVRS) | NIC,FIOCRUZ | |
| EPI_ISL_328569 | EPI1279442 | B/Yam | Brazil | 2018-Mar-12 | B/Espirito Santo/140/2018 | LACEN/ES - Laboratório Central de Saúde Pública do Espírito Santo | Instituto Oswaldo Cruz FIOCRUZ - Laboratory of Respiratory Viruses and Measles (LVRS) | NIC,FIOCRUZ | |
| EPI_ISL_328570 | EPI1279441 | B/Yam | Brazil | 2018-Feb-07 | B/Espirito Santo/87/2018 | LACEN/ES - Laboratório Central de Saúde Pública do Espírito Santo | Instituto Oswaldo Cruz FIOCRUZ - Laboratory of Respiratory Viruses and Measles (LVRS) | NIC,FIOCRUZ | |
| EPI_ISL_328571 | EPI1279440 | B/Yam | Brazil | 2018-Feb-06 | B/Espirito Santo/84/2018 | LACEN/ES - Laboratório Central de Saúde Pública do Espírito Santo | Instituto Oswaldo Cruz FIOCRUZ - Laboratory of Respiratory Viruses and Measles (LVRS) | NIC,FIOCRUZ | |
| EPI_ISL_328572 | EPI1279438 | B/Yam | Brazil | 2018-Jan-10 | B/Espirito Santo/48/2018 | LACEN/ES - Laboratório Central de Saúde Pública do Espírito Santo | Instituto Oswaldo Cruz FIOCRUZ - Laboratory of Respiratory Viruses and Measles (LVRS) | NIC,FIOCRUZ | |
| EPI_ISL_328573 | EPI1279437 | B/Yam | Brazil | 2018-Jan-03 | B/Espirito Santo/47/2018 | LACEN/ES - Laboratório Central de Saúde Pública do Espírito Santo | Instituto Oswaldo Cruz FIOCRUZ - Laboratory of Respiratory Viruses and Measles (LVRS) | NIC,FIOCRUZ | |
| EPI_ISL_328574 | EPI1305054 | B/Yam | Brazil | 2018-Jan-18 | B/Acre/151007-IEC/2018 | Evandro Chagas Institute | Evandro Chagas Institute |  | |
| EPI_ISL_329922 | EPI1311172 | B/Yam | Brazil | 2018-Jul-16 | B/Brazil/0379/2018 | Instituto Adolfo Lutz | Centers for Disease Control and Prevention |  | |
| EPI_ISL_330235 | EPI1312924 | B/Yam | Brazil | 2018-Jul-17 | B/Brazil/7103/2018 | Instituto Adolfo Lutz | Centers for Disease Control and Prevention |  | |
| EPI_ISL_330243 | EPI1312985 | B/Yam | Brazil | 2018-Aug-07 | B/Brazil/3033/2018 | Instituto Adolfo Lutz | Centers for Disease Control and Prevention |  | |
| EPI_ISL_330524 | EPI1314835 | B/Yam | Brazil | 2018-Aug-02 | B/Santa Catarina/1008/2018 | Instituto Oswaldo Cruz FIOCRUZ - Laboratory of Respiratory Viruses and Measles (LVRS) | Centers for Disease Control and Prevention |  | |
| EPI_ISL_330542 | EPI1314967 | B/Yam | Brazil | 2018-Jul-15 | B/Bahia/953/2018 | Instituto Oswaldo Cruz FIOCRUZ - Laboratory of Respiratory Viruses and Measles (LVRS) | Centers for Disease Control and Prevention |  | |
| EPI_ISL_304369 | EPI1279308 | A/H1pdm09 | Chile | 2018-Apr-16 | A/Vina_del_Mar/35661/2018 | Instituto de Salud Publica de Chile | Instituto de Salud Publica de Chile | Tognarelli J, Lagos J, Arata L, Fasce R, Fernandez J | |
| EPI_ISL_304370 | EPI1279045 | A/H1pdm09 | Chile | 2018-May-26 | A/Puerto_Montt/43843/2018 | Instituto de Salud Publica de Chile | Instituto de Salud Publica de Chile | Tognarelli J, Lagos J, Arata L, Fasce R, Fernandez J | |
| EPI_ISL_314523 | EPI1255847 | A/H1pdm09 | Chile | 2018-Apr-01 | A/Santiago/27760/2018 | Instituto de Salud Publica de Chile | Instituto de Salud Publica de Chile |  | |
| EPI_ISL_314541 | EPI1255845 | A/H1pdm09 | Chile | 2018-Feb-21 | A/Santiago/15780/201 | Instituto de Salud Publica de Chile | Instituto de Salud Publica de Chile |  | |
| EPI_ISL_314542 | EPI1255843 | A/H1pdm09 | Chile | 2018-Feb-19 | A/Santiago/15715/201 | Instituto de Salud Publica de Chile | Instituto de Salud Publica de Chile |  | |
| EPI_ISL_314546 | EPI1202681 | A/H1pdm09 | Chile | 2018-Jan-20 | A/PuertoMontt/6375/2018 | Instituto de Salud Publica de Chile | Instituto de Salud Publica de Chile |  | |
| EPI_ISL_314825 | EPI1202679 | A/H1pdm09 | Chile | 2018-Jan-09 | A/Santiago/2776/2018 | Instituto de Salud Publica de Chile | Instituto de Salud Publica de Chile |  | |
| EPI_ISL_314826 | EPI1282380 | A/H1pdm09 | Chile | 2018-Jul-17 | A/Santiago/59073/2018 | Instituto de Salud Publica de Chile | Centers for Disease Control and Prevention |  | |
| EPI_ISL_314827 | EPI1274419 | A/H1pdm09 | Chile | 2018-Jun-26 | A/Santiago/52554/2018 | Instituto de Salud Publica de Chile | Centers for Disease Control and Prevention |  | |
| EPI_ISL_315091 | EPI1274403 | A/H1pdm09 | Chile | 2018-Jul-11 | A/Santiago/56887/2018 | Instituto de Salud Publica de Chile | Centers for Disease Control and Prevention |  | |
| EPI_ISL_315094 | EPI1274395 | A/H1pdm09 | Chile | 2018-Jun-28 | A/Santiago/53037/2018 | Instituto de Salud Publica de Chile | Centers for Disease Control and Prevention |  | |
| EPI_ISL_315819 | EPI1274380 | A/H1pdm09 | Chile | 2018-Jul-09 | A/Santiago/55750/2018 | Instituto de Salud Publica de Chile | Centers for Disease Control and Prevention |  | |
| EPI_ISL_320709 | EPI1274356 | A/H1pdm09 | Chile | 2018-Jul-16 | A/Santiago/58061/2018 | Instituto de Salud Publica de Chile | Centers for Disease Control and Prevention |  | |
| EPI_ISL_321283 | EPI1271211 | A/H1pdm09 | Chile | 2018-Apr-19 | A/Antofagasta/37835/2018 | Instituto de Salud Publica de Chile | Centers for Disease Control and Prevention |  | |
| EPI_ISL_321286 | EPI1259464 | A/H1pdm09 | Chile | 2018-Apr-16 | A/Antofagasta/37826/2018 | Instituto de Salud Publica de Chile | Centers for Disease Control and Prevention |  | |
| EPI_ISL_321288 | EPI1257071 | A/H1pdm09 | Chile | 2018-Apr-28 | A/Santiago/35026/2018 | Instituto de Salud Publica de Chile | Centers for Disease Control and Prevention |  | |
| EPI_ISL_321289 | EPI1257052 | A/H1pdm09 | Chile | 2018-Apr-27 | A/Antofagasta/37838/2018 | Instituto de Salud Publica de Chile | Centers for Disease Control and Prevention |  | |
| EPI_ISL_321291 | EPI1254760 | A/H1pdm09 | Chile | 2018-Apr-23 | A/Santiago/33499/2018 | Instituto de Salud Publica de Chile | Centers for Disease Control and Prevention |  | |
| EPI_ISL_322134 | EPI1254730 | A/H1pdm09 | Chile | 2018-Apr-16 | A/Vinadelmar/35661/2018 | Instituto de Salud Publica de Chile | Centers for Disease Control and Prevention |  | |
| EPI_ISL_322167 | EPI1254722 | A/H1pdm09 | Chile | 2018-Apr-23 | A/Antofagasta/37828/2018 | Instituto de Salud Publica de Chile | Centers for Disease Control and Prevention |  | |
| EPI_ISL_322898 | EPI1254583 | A/H1pdm09 | Chile | 2018-Apr-04 | A/Santiago/27906/2018 | Instituto de Salud Publica de Chile | Centers for Disease Control and Prevention |  | |
| EPI_ISL_269745 | EPI1279323 | A/H3 | Chile | 2018-May-30 | A/Vina_del_Mar/48527/2018 | Instituto de Salud Publica de Chile | Instituto de Salud Publica de Chile | Tognarelli J, Lagos J, Arata L, Fasce R, Fernandez J | |
| EPI_ISL_269746 | EPI1279315 | A/H3 | Chile | 2018-May-30 | A/Vina_del_Mar/48525/2018 | Instituto de Salud Publica de Chile | Instituto de Salud Publica de Chile | Tognarelli J, Lagos J, Arata L, Fasce R, Fernandez J | |
| EPI_ISL_269747 | EPI1279165 | A/H3 | Chile | 2018-May-29 | A/Santiago/44033/2018 | Instituto de Salud Publica de Chile | Instituto de Salud Publica de Chile | Tognarelli J, Lagos J, Arata L, Fasce R, Fernandez J | |
| EPI_ISL_275770 | EPI1279109 | A/H3 | Chile | 2018-May-25 | A/Santiago/42956/2018 | Instituto de Salud Publica de Chile | Instituto de Salud Publica de Chile | Tognarelli J, Lagos J, Arata L, Fasce R, Fernandez J | |
| EPI_ISL_275791 | EPI1255879 | A/H3 | Chile | 2018-Apr-24 | A/Santiago/34512/2018 | Instituto de Salud Publica de Chile | Instituto de Salud Publica de Chile |  | |
| EPI_ISL_276045 | EPI1202712 | A/H3 | Chile | 2018-Jan-10 | A/Osorno/6007/2018 | Instituto de Salud Publica de Chile | Instituto de Salud Publica de Chile |  | |
| EPI_ISL_275893 | EPI1202711 | A/H3 | Chile | 2018-Jan-10 | A/PuertoMontt/2939/2018 | Instituto de Salud Publica de Chile | Instituto de Salud Publica de Chile |  | |
| EPI_ISL_275903 | EPI1202707 | A/H3 | Chile | 2017-Dec-21 | A/Iquique/1308/2017 | Instituto de Salud Publica de Chile | Instituto de Salud Publica de Chile |  | |
| EPI_ISL_275905 | EPI1202683 | A/H3 | Chile | 2017-Dec-07 | A/Concepcion/96590/2017 | Instituto de Salud Publica de Chile | Instituto de Salud Publica de Chile |  | |
| EPI_ISL_275917 | EPI1128359 | A/H3 | Chile | 2017-Nov-15 | A/PuertoMontt/91225/2017 | Instituto de Salud Publica de Chile | Instituto de Salud Publica de Chile |  | |
| EPI_ISL_275921 | EPI1128308 | A/H3 | Chile | 2017-Oct-30 | A/Iquique/90638/2017 | Instituto de Salud Publica de Chile | Instituto de Salud Publica de Chile |  | |
| EPI_ISL_275925 | EPI1127576 | A/H3 | Chile | 2017-Nov-23 | A/Coyhaique/90055/2017 | Instituto de Salud Publica de Chile | Instituto de Salud Publica de Chile |  | |
| EPI_ISL_275933 | EPI1127574 | A/H3 | Chile | 2017-Nov-22 | A/Santiago/89613/2017 | Instituto de Salud Publica de Chile | Instituto de Salud Publica de Chile |  | |
| EPI_ISL_282010 | EPI1127572 | A/H3 | Chile | 2017-Nov-16 | A/Santiago/87122/2017 | Instituto de Salud Publica de Chile | Instituto de Salud Publica de Chile |  | |
| EPI_ISL_286240 | EPI1127570 | A/H3 | Chile | 2017-Oct-06 | A/LaSerena/76599/2017 | Instituto de Salud Publica de Chile | Instituto de Salud Publica de Chile |  | |
| EPI_ISL_286245 | EPI1108100 | A/H3 | Chile | 2017-Sep-04 | A/Osorno/73747/2017 | Instituto de Salud Publica de Chile | Instituto de Salud Publica de Chile |  | |
| EPI_ISL_275897 | EPI1108098 | A/H3 | Chile | 2017-Sep-16 | A/Santiago/72273/2017 | Instituto de Salud Publica de Chile | Instituto de Salud Publica de Chile |  | |
| EPI_ISL_275909 | EPI1108096 | A/H3 | Chile | 2017-Sep-11 | A/VinadelMar/70327/2017 | Instituto de Salud Publica de Chile | Instituto de Salud Publica de Chile |  | |
| EPI_ISL_275915 | EPI1108094 | A/H3 | Chile | 2017-Aug-14 | A/Valparaiso/62593/2017 | Instituto de Salud Publica de Chile | Instituto de Salud Publica de Chile |  | |
| EPI_ISL_275919 | EPI1108092 | A/H3 | Chile | 2017-Aug-11 | A/Santiago/62354/2017 | Instituto de Salud Publica de Chile | Instituto de Salud Publica de Chile |  | |
| EPI_ISL_275935 | EPI1108090 | A/H3 | Chile | 2017-Aug-09 | A/LaSerena/61435/2017 | Instituto de Salud Publica de Chile | Instituto de Salud Publica de Chile |  | |
| EPI_ISL_269743 | EPI1108088 | A/H3 | Chile | 2017-Jul-22 | A/PuertoMontt/61063/2017 | Instituto de Salud Publica de Chile | Instituto de Salud Publica de Chile |  | |
| EPI_ISL_269744 | EPI1081418 | A/H3 | Chile | 2017-Jul-22 | A/LosAngeles/56834/2017 | Instituto de Salud Publica de Chile | Instituto de Salud Publica de Chile |  | |
| EPI_ISL_275699 | EPI1081406 | A/H3 | Chile | 2017-Jul-19 | A/PuntaArena/55840/2017 | Instituto de Salud Publica de Chile | Instituto de Salud Publica de Chile |  | |
| EPI_ISL_275752 | EPI1081404 | A/H3 | Chile | 2017-Jul-12 | A/Chillan/55269/2017 | Instituto de Salud Publica de Chile | Instituto de Salud Publica de Chile |  | |
| EPI_ISL_275769 | EPI1049925 | A/H3 | Chile | 2017-Jun-19 | A/LaSerena/47365/2017 | Instituto de Salud Publica de Chile | Instituto de Salud Publica de Chile |  | |
| EPI_ISL_276044 | EPI1049923 | A/H3 | Chile | 2017-Jul-14 | A/Chillan/47248/2017 | Instituto de Salud Publica de Chile | Instituto de Salud Publica de Chile |  | |
| EPI_ISL_282017 | EPI1048247 | A/H3 | Chile | 2017-Jun-12 | A/Coronel/47245/2017 | Instituto de Salud Publica de Chile | Instituto de Salud Publica de Chile |  | |
| EPI_ISL_275895 | EPI1048090 | A/H3 | Chile | 2017-Jun-12 | A/Concepcion/47230/2017 | Instituto de Salud Publica de Chile | Instituto de Salud Publica de Chile |  | |
| EPI_ISL_275931 | EPI1048077 | A/H3 | Chile | 2017-Jun-16 | A/Talca/47223/2017 | Instituto de Salud Publica de Chile | Instituto de Salud Publica de Chile |  | |
| EPI_ISL_275939 | EPI1047969 | A/H3 | Chile | 2017-Jun-16 | A/Ovalle/47167/2017 | Instituto de Salud Publica de Chile | Instituto de Salud Publica de Chile |  | |
| EPI_ISL_314837 | EPI1047773 | A/H3 | Chile | 2017-Jun-13 | A/PuntaArenas/47087/2017 | Instituto de Salud Publica de Chile | Instituto de Salud Publica de Chile |  | |
| EPI_ISL_322142 | EPI1047525 | A/H3 | Chile | 2017-Jun-04 | A/Santiago/42544/2017 | Instituto de Salud Publica de Chile | Instituto de Salud Publica de Chile |  | |
| EPI_ISL_322149 | EPI1047469 | A/H3 | Chile | 2017-May-22 | A/Santiago/40005/2017 | Instituto de Salud Publica de Chile | Instituto de Salud Publica de Chile |  | |
| EPI_ISL_322168 | EPI1047459 | A/H3 | Chile | 2017-May-23 | A/Curico/39966/2017 | Instituto de Salud Publica de Chile | Instituto de Salud Publica de Chile |  | |
| EPI_ISL_322169 | EPI1047432 | A/H3 | Chile | 2017-May-17 | A/Osorno/39912/2017 | Instituto de Salud Publica de Chile | Instituto de Salud Publica de Chile |  | |
| EPI_ISL_321219 | EPI1023693 | A/H3 | Chile | 2017-May-11 | A/Antofagasta/39803/2017 | Instituto de Salud Publica de Chile | Instituto de Salud Publica de Chile |  | |
| EPI_ISL_321221 | EPI1023690 | A/H3 | Chile | 2017-May-23 | A/Talca/39544/2017 | Instituto de Salud Publica de Chile | Instituto de Salud Publica de Chile |  | |
| EPI_ISL_321223 | EPI1023688 | A/H3 | Chile | 2017-May-21 | A/Rancagua/39432/2017 | Instituto de Salud Publica de Chile | Instituto de Salud Publica de Chile |  | |
| EPI_ISL_321229 | EPI1023686 | A/H3 | Chile | 2017-May-22 | A/PuntaArenas/39178/2017 | Instituto de Salud Publica de Chile | Instituto de Salud Publica de Chile |  | |
| EPI_ISL_321235 | EPI1023684 | A/H3 | Chile | 2017-May-04 | A/Concepcion/38299/2017 | Instituto de Salud Publica de Chile | Instituto de Salud Publica de Chile |  | |
| EPI_ISL_275696 | EPI1273972 | A/H3 | Chile | 2018-Jun-21 | A/Vina Del Mar/52623/2018 | Instituto de Salud Publica de Chile | Centers for Disease Control and Prevention |  | |
| EPI_ISL_275698 | EPI1273924 | A/H3 | Chile | 2018-Jul-10 | A/Antofagasta/58996/2018 | Instituto de Salud Publica de Chile | Centers for Disease Control and Prevention |  | |
| EPI_ISL_275700 | EPI1273876 | A/H3 | Chile | 2018-May-14 | A/Santiago/38879/2018 | Instituto de Salud Publica de Chile | Centers for Disease Control and Prevention |  | |
| EPI_ISL_275723 | EPI1273860 | A/H3 | Chile | 2018-Jul-23 | A/Santiago/59907/2018 | Instituto de Salud Publica de Chile | Centers for Disease Control and Prevention |  | |
| EPI_ISL_282011 | EPI1273844 | A/H3 | Chile | 2018-Jul-18 | A/La Serena/60001/2018 | Instituto de Salud Publica de Chile | Centers for Disease Control and Prevention |  | |
| EPI_ISL_286241 | EPI1049416 | A/H3 | Chile | 2017-Jul-22 | A/Los Angeles/56834/2017 | Instituto de Salud Publica de Chile | Centers for Disease Control and Prevention |  | |
| EPI_ISL_286242 | EPI1049386 | A/H3 | Chile | 2017-Jul-17 | A/Santiago/56159/2017 | Instituto de Salud Publica de Chile | Centers for Disease Control and Prevention |  | |
| EPI_ISL_286243 | EPI1049369 | A/H3 | Chile | 2017-Jul-23 | A/Santiago/56133/2017 | Instituto de Salud Publica de Chile | Centers for Disease Control and Prevention |  | |
| EPI_ISL_286244 | EPI1049357 | A/H3 | Chile | 2017-Jul-04 | A/Iquique/56115/2017 | Instituto de Salud Publica de Chile | Centers for Disease Control and Prevention |  | |
| EPI_ISL_286246 | EPI1049310 | A/H3 | Chile | 2017-Jul-02 | A/Copiapo/56066/2017 | Instituto de Salud Publica de Chile | Centers for Disease Control and Prevention |  | |
| EPI_ISL_289185 | EPI1049279 | A/H3 | Chile | 2017-Jul-15 | A/Rengo/55681/2017 | Instituto de Salud Publica de Chile | Centers for Disease Control and Prevention |  | |
| EPI_ISL_289186 | EPI1049264 | A/H3 | Chile | 2017-Jul-11 | A/Paillaco/55174/2017 | Instituto de Salud Publica de Chile | Centers for Disease Control and Prevention |  | |
| EPI_ISL_289187 | EPI1049247 | A/H3 | Chile | 2017-Jul-03 | A/Valdivia/55159/2017 | Instituto de Salud Publica de Chile | Centers for Disease Control and Prevention |  | |
| EPI_ISL_289188 | EPI1049230 | A/H3 | Chile | 2017-Jun-21 | A/Linares/55156/2017 | Instituto de Salud Publica de Chile | Centers for Disease Control and Prevention |  | |
| EPI_ISL_289276 | EPI1049183 | A/H3 | Chile | 2017-Jul-10 | A/Talca/55005/2017 | Instituto de Salud Publica de Chile | Centers for Disease Control and Prevention |  | |
| EPI_ISL_289288 | EPI1049152 | A/H3 | Chile | 2017-Jul-12 | A/Concepcion/54975/2017 | Instituto de Salud Publica de Chile | Centers for Disease Control and Prevention |  | |
| EPI_ISL_304371 | EPI1049137 | A/H3 | Chile | 2017-Jul-15 | A/Valparaiso/54802/2017 | Instituto de Salud Publica de Chile | Centers for Disease Control and Prevention |  | |
| EPI_ISL_304394 | EPI1049088 | A/H3 | Chile | 2017-Jul-06 | A/Rengo/53924/2017 | Instituto de Salud Publica de Chile | Centers for Disease Control and Prevention |  | |
| EPI_ISL_304396 | EPI1049071 | A/H3 | Chile | 2017-Jul-11 | A/Santiago/53918/2017 | Instituto de Salud Publica de Chile | Centers for Disease Control and Prevention |  | |
| EPI_ISL_304397 | EPI1049054 | A/H3 | Chile | 2017-Jun-22 | A/Rancagua/53519/2017 | Instituto de Salud Publica de Chile | Centers for Disease Control and Prevention |  | |
| EPI_ISL_314831 | EPI1281248 | A/H3 | Chile | 2018-Apr-02 | A/Alto_Hospicio/32463/2018 | Instituto de Salud Publica de Chile | Instituto de Salud Publica de Chile | Tognarelli J, Lagos J, Arata L, Fasce R, Fernandez J | |
| EPI_ISL_314833 | EPI1279331 | A/H3 | Chile | 2018-May-30 | A/Vina_del_Mar/48529/2018 | Instituto de Salud Publica de Chile | Instituto de Salud Publica de Chile | Tognarelli J, Lagos J, Arata L, Fasce R, Fernandez J | |
| EPI_ISL_314834 | EPI1279293 | A/H3 | Chile | 2018-Jun-08 | A/Concepcion/48815/2018 | Instituto de Salud Publica de Chile | Instituto de Salud Publica de Chile | Tognarelli J, Lagos J, Arata L, Fasce R, Fernandez J | |
| EPI_ISL_314836 | EPI1279285 | A/H3 | Chile | 2018-Jun-06 | A/Concepcion/48747/2018 | Instituto de Salud Publica de Chile | Instituto de Salud Publica de Chile | Tognarelli J, Lagos J, Arata L, Fasce R, Fernandez J | |
| EPI_ISL_315086 | EPI1279277 | A/H3 | Chile | 2018-May-29 | A/Iquique/48545/2018 | Instituto de Salud Publica de Chile | Instituto de Salud Publica de Chile | Tognarelli J, Lagos J, Arata L, Fasce R, Fernandez J | |
| EPI_ISL_315087 | EPI1279253 | A/H3 | Chile | 2018-May-24 | A/Temuco/47994/2018 | Instituto de Salud Publica de Chile | Instituto de Salud Publica de Chile | Tognarelli J, Lagos J, Arata L, Fasce R, Fernandez J | |
| EPI_ISL_315088 | EPI1279245 | A/H3 | Chile | 2018-Jun-07 | A/Concepcion/47476/2018 | Instituto de Salud Publica de Chile | Instituto de Salud Publica de Chile | Tognarelli J, Lagos J, Arata L, Fasce R, Fernandez J | |
| EPI_ISL_315089 | EPI1279237 | A/H3 | Chile | 2018-May-30 | A/Puerto_Montt/46753/2018 | Instituto de Salud Publica de Chile | Instituto de Salud Publica de Chile | Tognarelli J, Lagos J, Arata L, Fasce R, Fernandez J | |
| EPI_ISL_315090 | EPI1279229 | A/H3 | Chile | 2018-May-30 | A/Concepcion/45941/2018 | Instituto de Salud Publica de Chile | Instituto de Salud Publica de Chile | Tognarelli J, Lagos J, Arata L, Fasce R, Fernandez J | |
| EPI_ISL_315095 | EPI1279221 | A/H3 | Chile | 2018-May-22 | A/Concepcion/45013/2018 | Instituto de Salud Publica de Chile | Instituto de Salud Publica de Chile | Tognarelli J, Lagos J, Arata L, Fasce R, Fernandez J | |
| EPI_ISL_315097 | EPI1279213 | A/H3 | Chile | 2018-May-29 | A/Concepcion/44979/2018 | Instituto de Salud Publica de Chile | Instituto de Salud Publica de Chile | Tognarelli J, Lagos J, Arata L, Fasce R, Fernandez J | |
| EPI_ISL_315099 | EPI1279205 | A/H3 | Chile | 2018-May-28 | A/Concepcion/44977/2018 | Instituto de Salud Publica de Chile | Instituto de Salud Publica de Chile | Tognarelli J, Lagos J, Arata L, Fasce R, Fernandez J | |
| EPI_ISL_322137 | EPI1279197 | A/H3 | Chile | 2018-May-23 | A/Alto_Hospicio/44625/2018 | Instituto de Salud Publica de Chile | Instituto de Salud Publica de Chile | Tognarelli J, Lagos J, Arata L, Fasce R, Fernandez J | |
| EPI_ISL_322138 | EPI1279189 | A/H3 | Chile | 2018-May-27 | A/Iquique/44553/2018 | Instituto de Salud Publica de Chile | Instituto de Salud Publica de Chile | Tognarelli J, Lagos J, Arata L, Fasce R, Fernandez J | |
| EPI_ISL_322151 | EPI1279181 | A/H3 | Chile | 2018-May-14 | A/Iquique/44550/2018 | Instituto de Salud Publica de Chile | Instituto de Salud Publica de Chile | Tognarelli J, Lagos J, Arata L, Fasce R, Fernandez J | |
| EPI_ISL_275891 | EPI1279173 | A/H3 | Chile | 2018-May-15 | A/Iquique/44531/2018 | Instituto de Salud Publica de Chile | Instituto de Salud Publica de Chile | Tognarelli J, Lagos J, Arata L, Fasce R, Fernandez J | |
| EPI_ISL_275901 | EPI1279157 | A/H3 | Chile | 2018-May-14 | A/Concepcion/44028/2018 | Instituto de Salud Publica de Chile | Instituto de Salud Publica de Chile | Tognarelli J, Lagos J, Arata L, Fasce R, Fernandez J | |
| EPI_ISL_275907 | EPI1279149 | A/H3 | Chile | 2018-May-23 | A/Concepcion/44024/2018 | Instituto de Salud Publica de Chile | Instituto de Salud Publica de Chile | Tognarelli J, Lagos J, Arata L, Fasce R, Fernandez J | |
| EPI_ISL_275913 | EPI1279141 | A/H3 | Chile | 2018-May-22 | A/Concepcion/44021/2018 | Instituto de Salud Publica de Chile | Instituto de Salud Publica de Chile | Tognarelli J, Lagos J, Arata L, Fasce R, Fernandez J | |
| EPI_ISL_275923 | EPI1279133 | A/H3 | Chile | 2018-May-21 | A/Concepcion/44019/2018 | Instituto de Salud Publica de Chile | Instituto de Salud Publica de Chile | Tognarelli J, Lagos J, Arata L, Fasce R, Fernandez J | |
| EPI_ISL_275937 | EPI1279125 | A/H3 | Chile | 2018-May-02 | A/Concepcion/44012/2018 | Instituto de Salud Publica de Chile | Instituto de Salud Publica de Chile | Tognarelli J, Lagos J, Arata L, Fasce R, Fernandez J | |
| EPI_ISL_281532 | EPI1279117 | A/H3 | Chile | 2018-May-18 | A/Curanilahue/44002/2018 | Instituto de Salud Publica de Chile | Instituto de Salud Publica de Chile | Tognarelli J, Lagos J, Arata L, Fasce R, Fernandez J | |
| EPI_ISL_290657 | EPI1279101 | A/H3 | Chile | 2018-May-08 | A/Concepcion/40308/2018 | Instituto de Salud Publica de Chile | Instituto de Salud Publica de Chile | Tognarelli J, Lagos J, Arata L, Fasce R, Fernandez J | |
| EPI_ISL_321226 | EPI1279093 | A/H3 | Chile | 2018-Apr-19 | A/Alto_Hospicio/37840/2018 | Instituto de Salud Publica de Chile | Instituto de Salud Publica de Chile | Tognarelli J, Lagos J, Arata L, Fasce R, Fernandez J | |
| EPI_ISL_321227 | EPI1279085 | A/H3 | Chile | 2018-Apr-21 | A/Alto_Hospicio/37839/2018 | Instituto de Salud Publica de Chile | Instituto de Salud Publica de Chile | Tognarelli J, Lagos J, Arata L, Fasce R, Fernandez J | |
| EPI_ISL_321231 | EPI1279077 | A/H3 | Chile | 2018-Apr-04 | A/Alto_Hospicio/32457/2018 | Instituto de Salud Publica de Chile | Instituto de Salud Publica de Chile | Tognarelli J, Lagos J, Arata L, Fasce R, Fernandez J | |
| EPI_ISL_321236 | EPI1279069 | A/H3 | Chile | 2018-Apr-02 | A/Iquique/32413/2018 | Instituto de Salud Publica de Chile | Instituto de Salud Publica de Chile | Tognarelli J, Lagos J, Arata L, Fasce R, Fernandez J | |
| EPI_ISL_304402 | EPI1279061 | A/H3 | Chile | 2018-Apr-06 | A/Concepcion/32371/2018 | Instituto de Salud Publica de Chile | Instituto de Salud Publica de Chile | Tognarelli J, Lagos J, Arata L, Fasce R, Fernandez J | |
| EPI_ISL_304403 | EPI1279053 | A/H3 | Chile | 2018-Apr-17 | A/Concepcion/32368/2018 | Instituto de Salud Publica de Chile | Instituto de Salud Publica de Chile | Tognarelli J, Lagos J, Arata L, Fasce R, Fernandez J | |
| EPI_ISL_314832 | EPI1257115 | A/H3 | Chile | 2018-Jan-31 | A/Santiago/9536/2018 | Instituto de Salud Publica de Chile | Instituto de Salud Publica de Chile | Tognarelli J, Lagos J, Arata L, Fasce R, Fernandez J | |
| EPI_ISL_314835 | EPI1257099 | A/H3 | Chile | 2018-Feb-01 | A/Santiago/9435/2018 | Instituto de Salud Publica de Chile | Instituto de Salud Publica de Chile | Tognarelli J, Lagos J, Arata L, Fasce R, Fernandez J | |
| EPI_ISL_314838 | EPI1257083 | A/H3 | Chile | 2018-Jan-20 | A/Santiago/7340/2018 | Instituto de Salud Publica de Chile | Instituto de Salud Publica de Chile | Tognarelli J, Lagos J, Arata L, Fasce R, Fernandez J | |
| EPI_ISL_314839 | EPI1257067 | A/H3 | Chile | 2018-Jan-20 | A/Santiago/7339/2018 | Instituto de Salud Publica de Chile | Instituto de Salud Publica de Chile | Tognarelli J, Lagos J, Arata L, Fasce R, Fernandez J | |
| EPI_ISL_315092 | EPI1257051 | A/H3 | Chile | 2018-Jan-20 | A/Santiago/7338/2018 | Instituto de Salud Publica de Chile | Instituto de Salud Publica de Chile | Tognarelli J, Lagos J, Arata L, Fasce R, Fernandez J | |
| EPI_ISL_315093 | EPI1257036 | A/H3 | Chile | 2018-Jan-06 | A/Santiago/2775/2018 | Instituto de Salud Publica de Chile | Instituto de Salud Publica de Chile | Tognarelli J, Lagos J, Arata L, Fasce R, Fernandez J | |
| EPI_ISL_322135 | EPI1257028 | A/H3 | Chile | 2017-Dec-21 | A/Iquique/1308/2018 | Instituto de Salud Publica de Chile | Instituto de Salud Publica de Chile | Tognarelli J, Lagos J, Arata L, Fasce R, Fernandez J | |
| EPI_ISL_322136 | EPI1257015 | A/H3 | Chile | 2017-Dec-27 | A/Santiago/162/2018 | Instituto de Salud Publica de Chile | Instituto de Salud Publica de Chile | Tognarelli J, Lagos J, Arata L, Fasce R, Fernandez J | |
| EPI_ISL_322139 | EPI1257004 | A/H3 | Chile | 2017-Dec-24 | A/Santiago/152/2018 | Instituto de Salud Publica de Chile | Instituto de Salud Publica de Chile | Tognarelli J, Lagos J, Arata L, Fasce R, Fernandez J | |
| EPI_ISL_322140 | EPI1255883 | A/H3 | Chile | 2018-Apr-23 | A/Osorno/35798/2018 | Instituto de Salud Publica de Chile | Instituto de Salud Publica de Chile |  | |
| EPI_ISL_322141 | EPI1255881 | A/H3 | Chile | 2018-May-02 | A/Valparaiso/35691/2018 | Instituto de Salud Publica de Chile | Instituto de Salud Publica de Chile |  | |
| EPI_ISL_322143 | EPI1255877 | A/H3 | Chile | 2018-Apr-03 | A/Rancagua/29452/2018 | Instituto de Salud Publica de Chile | Instituto de Salud Publica de Chile |  | |
| EPI_ISL_322144 | EPI1255875 | A/H3 | Chile | 2018-Mar-22 | A/Iquique/27192/2018 | Instituto de Salud Publica de Chile | Instituto de Salud Publica de Chile |  | |
| EPI_ISL_322145 | EPI1255873 | A/H3 | Chile | 2018-Mar-28 | A/Antofagasta/27188/2018 | Instituto de Salud Publica de Chile | Instituto de Salud Publica de Chile |  | |
| EPI_ISL_322146 | EPI1255871 | A/H3 | Chile | 2018-Mar-16 | A/PuntaArenas/23392/2018 | Instituto de Salud Publica de Chile | Instituto de Salud Publica de Chile |  | |
| EPI_ISL_322147 | EPI1255869 | A/H3 | Chile | 2018-Mar-18 | A/Concepcion/22807/2018 | Instituto de Salud Publica de Chile | Instituto de Salud Publica de Chile |  | |
| EPI_ISL_322148 | EPI1255867 | A/H3 | Chile | 2018-Mar-01 | A/Santiago/17989/2018 | Instituto de Salud Publica de Chile | Instituto de Salud Publica de Chile |  | |
| EPI_ISL_322150 | EPI1202722 | A/H3 | Chile | 2018-Jan-25 | A/PuntaArenas/10965/2018 | Instituto de Salud Publica de Chile | Instituto de Salud Publica de Chile |  | |
| EPI_ISL_322152 | EPI1202720 | A/H3 | Chile | 2018-Jan-22 | A/Talca/7105/2018 | Instituto de Salud Publica de Chile | Instituto de Salud Publica de Chile |  | |
| EPI_ISL_322153 | EPI1257027 | A/H3 | Ukraine | 2018-Apr-25 | A/Dnipro/415/2018 | Institute of Epidemiology and Infectious Diseases AMS of Ukraine | Centers for Disease Control and Prevention |  | |
| EPI_ISL_322154 | EPI1273980 | A/H3 | Chile | 2018-Jun-27 | A/Santiago/52649/2018 | Instituto de Salud Publica de Chile | Centers for Disease Control and Prevention |  | |
| EPI_ISL_322155 | EPI1273940 | A/H3 | Chile | 2018-Jul-26 | A/Santiago/61477/2018 | Instituto de Salud Publica de Chile | Centers for Disease Control and Prevention |  | |
| EPI_ISL_322156 | EPI1273908 | A/H3 | Chile | 2018-Jul-24 | A/Valparaiso/60603/2018 | Instituto de Salud Publica de Chile | Centers for Disease Control and Prevention |  | |
| EPI_ISL_322157 | EPI1273900 | A/H3 | Chile | 2018-Jul-08 | A/Rancagua/60646/2018 | Instituto de Salud Publica de Chile | Centers for Disease Control and Prevention |  | |
| EPI_ISL_322158 | EPI1136420 | A/H3 | Chile | 2017-Oct-06 | A/La Serena/76599/2017 | Instituto de Salud Publica de Chile | Centers for Disease Control and Prevention |  | |
| EPI_ISL_322159 | EPI1077871 | A/H3 | Chile | 2017-Jul-10 | A/Rio Bueno/56967/2017 | Instituto de Salud Publica de Chile | Centers for Disease Control and Prevention |  | |
| EPI_ISL_322160 | EPI1049402 | A/H3 | Chile | 2017-Jul-21 | A/Santiago/56525/2017 | Instituto de Salud Publica de Chile | Centers for Disease Control and Prevention |  | |
| EPI_ISL_322163 | EPI1049295 | A/H3 | Chile | 2017-Jul-19 | A/Punta Arenas/55840/2017 | Instituto de Salud Publica de Chile | Centers for Disease Control and Prevention |  | |
| EPI_ISL_322164 | EPI1049211 | A/H3 | Chile | 2017-Jul-13 | A/Rancagua/55146/2017 | Instituto de Salud Publica de Chile | Centers for Disease Control and Prevention |  | |
| EPI_ISL_322165 | EPI1049167 | A/H3 | Chile | 2017-Jul-12 | A/Lota/54994/2017 | Instituto de Salud Publica de Chile | Centers for Disease Control and Prevention |  | |
| EPI_ISL_322170 | EPI1049120 | A/H3 | Chile | 2017-Jul-12 | A/Coyhaique/54224/2017 | Instituto de Salud Publica de Chile | Centers for Disease Control and Prevention |  | |
| EPI_ISL_322593 | EPI1049039 | A/H3 | Chile | 2017-Jun-19 | A/Santiago/46637/2017 | Instituto de Salud Publica de Chile | Centers for Disease Control and Prevention |  | |
| EPI_ISL_322594 | EPI1281253 | A/H3 | Chile | 2018-May-25 | A/Vina_del_Mar/48528/2018 | Instituto de Salud Publica de Chile | Instituto de Salud Publica de Chile | Tognarelli J, Lagos J, Arata L, Fasce R, Fernandez J | |
| EPI_ISL_322595 | EPI1281252 | A/H3 | Chile | 2018-Apr-25 | A/Concepcion/44232/2018 | Instituto de Salud Publica de Chile | Instituto de Salud Publica de Chile | Tognarelli J, Lagos J, Arata L, Fasce R, Fernandez J | |
| EPI_ISL_322596 | EPI1281250 | A/H3 | Chile | 2018-Apr-04 | A/Alto_Hospicio/32520/2018 | Instituto de Salud Publica de Chile | Instituto de Salud Publica de Chile | Tognarelli J, Lagos J, Arata L, Fasce R, Fernandez J | |
| EPI_ISL_322597 | EPI1281249 | A/H3 | Chile | 2018-Mar-31 | A/Alto_Hospicio/32464/2018 | Instituto de Salud Publica de Chile | Instituto de Salud Publica de Chile | Tognarelli J, Lagos J, Arata L, Fasce R, Fernandez J | |
| EPI_ISL_315000 | EPI1108086 | A/H3 | Chile | 2017-Jul-31 | A/Antofagasta/60978/2017 | Instituto de Salud Publica de Chile | Instituto de Salud Publica de Chile |  | |
| EPI_ISL_315015 | EPI1108084 | A/H3 | Chile | 2017-Jul-30 | A/Iquique/60972/2017 | Instituto de Salud Publica de Chile | Instituto de Salud Publica de Chile |  | |
| EPI_ISL_315016 | EPI1108082 | A/H3 | Chile | 2017-Jul-12 | A/Osorno/60580/2017 | Instituto de Salud Publica de Chile | Instituto de Salud Publica de Chile |  | |
| EPI_ISL_315021 | EPI1081410 | A/H3 | Chile | 2017-Jul-03 | A/Iquique/56076/2017 | Instituto de Salud Publica de Chile | Instituto de Salud Publica de Chile |  | |
| EPI_ISL_315027 | EPI1049927 | A/H3 | Chile | 2017-Jun-16 | A/VinadelMar/47400/2017 | Instituto de Salud Publica de Chile | Instituto de Salud Publica de Chile |  | |
| EPI_ISL_315041 | EPI1047584 | A/H3 | Chile | 2017-Jun-11 | A/Osorno/46738/2017 | Instituto de Salud Publica de Chile | Instituto de Salud Publica de Chile |  | |
| EPI_ISL_321218 | EPI1047435 | A/H3 | Chile | 2017-May-16 | A/Calama/39937/2017 | Instituto de Salud Publica de Chile | Instituto de Salud Publica de Chile |  | |
| EPI_ISL_321224 | EPI1023704 | A/H3 | Chile | 2017-May-23 | A/Vaparaiso/39896/2017 | Instituto de Salud Publica de Chile | Instituto de Salud Publica de Chile |  | |
| EPI_ISL_321230 | EPI1274140 | A/H3 | Chile | 2018-Jul-11 | A/Puerto Montt/59138/2018 | Instituto de Salud Publica de Chile | Centers for Disease Control and Prevention |  | |
| EPI_ISL_321232 | EPI1274020 | A/H3 | Chile | 2018-Jul-08 | A/Calama/59025/2018 | Instituto de Salud Publica de Chile | Centers for Disease Control and Prevention |  | |
| EPI_ISL_321233 | EPI1274012 | A/H3 | Chile | 2018-Jul-22 | A/Vina Del Mar/60406/2018 | Instituto de Salud Publica de Chile | Centers for Disease Control and Prevention |  | |
| EPI_ISL_321234 | EPI1273964 | A/H3 | Chile | 2018-Jun-27 | A/Rancagua/54068/2018 | Instituto de Salud Publica de Chile | Centers for Disease Control and Prevention |  | |
| EPI_ISL_321240 | EPI1273956 | A/H3 | Chile | 2018-Jun-22 | A/Lota/52321/2018 | Instituto de Salud Publica de Chile | Centers for Disease Control and Prevention |  | |
| EPI_ISL_321241 | EPI1273948 | A/H3 | Chile | 2018-Jul-21 | A/Curico/60608/2018 | Instituto de Salud Publica de Chile | Centers for Disease Control and Prevention |  | |
| EPI_ISL_321256 | EPI1273932 | A/H3 | Chile | 2018-Jun-28 | A/Valparaiso/54264/2018 | Instituto de Salud Publica de Chile | Centers for Disease Control and Prevention |  | |
| EPI_ISL_269752 | EPI1273884 | A/H3 | Chile | 2018-Jun-28 | A/Osorno/54626/2018 | Instituto de Salud Publica de Chile | Centers for Disease Control and Prevention |  | |
| EPI_ISL_275697 | EPI1273836 | A/H3 | Chile | 2018-Jul-24 | A/Santiago/60852/2018 | Instituto de Salud Publica de Chile | Centers for Disease Control and Prevention |  | |
| EPI_ISL_275702 | EPI1256644 | A/H3 | Chile | 2018-Mar-05 | A/Coyhaique/36501/2018 | Instituto de Salud Publica de Chile | Centers for Disease Control and Prevention |  | |
| EPI_ISL_276046 | EPI1256532 | A/H3 | Chile | 2018-Apr-19 | A/Alto Hospicio/37840/2018 | Instituto de Salud Publica de Chile | Centers for Disease Control and Prevention |  | |
| EPI_ISL_282013 | EPI1256485 | A/H3 | Chile | 2018-Apr-17 | A/Conception/32368/2018 | Instituto de Salud Publica de Chile | Centers for Disease Control and Prevention |  | |
| EPI_ISL_286237 | EPI1256445 | A/H3 | Chile | 2018-Apr-27 | A/Iquique/37845/2018 | Instituto de Salud Publica de Chile | Centers for Disease Control and Prevention |  | |
| EPI_ISL_286238 | EPI1256438 | A/H3 | Chile | 2018-Apr-22 | A/Iquique/37846/2018 | Instituto de Salud Publica de Chile | Centers for Disease Control and Prevention |  | |
| EPI_ISL_286239 | EPI1256319 | A/H3 | Chile | 2018-May-14 | A/Coyhaique/39731/2018 | Instituto de Salud Publica de Chile | Centers for Disease Control and Prevention |  | |
| EPI_ISL_275899 | EPI1049340 | A/H3 | Chile | 2017-Jun-27 | A/Calama/56093/2017 | Instituto de Salud Publica de Chile | Centers for Disease Control and Prevention |  | |
| EPI_ISL_275911 | EPI1049325 | A/H3 | Chile | 2017-Jul-03 | A/Alto Hospicio/56076/2017 | Instituto de Salud Publica de Chile | Centers for Disease Control and Prevention |  | |
| EPI_ISL_275927 | EPI1049197 | A/H3 | Chile | 2017-Jul-12 | A/Vina Del Mar/55113/2017 | Instituto de Salud Publica de Chile | Centers for Disease Control and Prevention |  | |
| EPI_ISL_275929 | EPI1049104 | A/H3 | Chile | 2017-Jul-13 | A/La Serena/54022/2017 | Instituto de Salud Publica de Chile | Centers for Disease Control and Prevention |  | |
| EPI_ISL_269749 | EPI1023697 | B/Vic | Chile | 2017-May-05 | B/Santiago/34576/2017 | Instituto de Salud Publica de Chile | Instituto de Salud Publica de Chile |  | |
| EPI_ISL_276560 | EPI1127568 | B/Vic | Chile | 2017-Nov-08 | B/Rancagua/90687/2017 | Instituto de Salud Publica de Chile | Instituto de Salud Publica de Chile |  | |
| EPI_ISL_276561 | EPI1202902 | B/Vic | Chile | 2017-Jun-09 | B/VinadelMar/45053/2017 | Instituto de Salud Publica de Chile | Instituto de Salud Publica de Chile |  | |
| EPI_ISL_289184 | EPI1202904 | B/Vic | Chile | 2017-Dec-29 | B/Santiago/65/2017 | Instituto de Salud Publica de Chile | Instituto de Salud Publica de Chile |  | |
| EPI_ISL_304483 | EPI1255897 | B/Vic | Chile | 2018-Apr-18 | B/PuntaArenas/33524/2018 | Instituto de Salud Publica de Chile | Instituto de Salud Publica de Chile |  | |
| EPI_ISL_304484 | EPI1256716 | B/Vic | Chile | 2017-Dec-29 | B/Santiago/65/2018 | Instituto de Salud Publica de Chile | Instituto de Salud Publica de Chile | Tognarelli J, Lagos J, Arata L, Fasce R, Fernandez J | |
| EPI_ISL_314439 | EPI1256764 | B/Vic | Chile | 2018-Jan-02 | B/Valparaiso/958/2018 | Instituto de Salud Publica de Chile | Instituto de Salud Publica de Chile | Tognarelli J, Lagos J, Arata L, Fasce R, Fernandez J | |
| EPI_ISL_314440 | EPI1052816 | B/Vic | Chile | 2017-Jul-03 | B/Vina Del Mar/51186/2017 | Instituto de Salud Publica de Chile | Centers for Disease Control and Prevention |  | |
| EPI_ISL_314846 | EPI1052824 | B/Vic | Chile | 2017-Jul-10 | B/Santiago/54067/2017 | Instituto de Salud Publica de Chile | Centers for Disease Control and Prevention |  | |
| EPI_ISL_315050 | EPI1253949 | B/Vic | Chile | 2018-Apr-24 | B/Santiago/34511/2018 | Instituto de Salud Publica de Chile | Centers for Disease Control and Prevention |  | |
| EPI_ISL_315056 | EPI1253956 | B/Vic | Chile | 2018-Apr-18 | B/Punta Arenas/33524/2018 | Instituto de Salud Publica de Chile | Centers for Disease Control and Prevention |  | |
| EPI_ISL_321157 | EPI1273354 | B/Vic | Chile | 2018-Jun-05 | B/Santiago/47361/2018 | Instituto de Salud Publica de Chile | Centers for Disease Control and Prevention |  | |
| EPI_ISL_321158 | EPI1273362 | B/Vic | Chile | 2018-Jul-13 | B/Punta Arenas/58278/2018 | Instituto de Salud Publica de Chile | Centers for Disease Control and Prevention |  | |
| EPI_ISL_321159 | EPI1273370 | B/Vic | Chile | 2018-Jul-09 | B/Valparaiso/55955/2018 | Instituto de Salud Publica de Chile | Centers for Disease Control and Prevention |  | |
| EPI_ISL_321180 | EPI1273538 | B/Vic | Chile | 2018-Jun-22 | B/Santiago/51375/2018 | Instituto de Salud Publica de Chile | Centers for Disease Control and Prevention |  | |
| EPI_ISL_269750 | EPI1127566 | B/Yam | Chile | 2017-Nov-13 | B/Panguipulli/90686/2017 | Instituto de Salud Publica de Chile | Instituto de Salud Publica de Chile |  | |
| EPI_ISL_269751 | EPI1127564 | B/Yam | Chile | 2017-Nov-28 | B/Valparaiso/90673/2017 | Instituto de Salud Publica de Chile | Instituto de Salud Publica de Chile |  | |
| EPI_ISL_276051 | EPI1127562 | B/Yam | Chile | 2017-Nov-04 | B/Iquique/90647/2017 | Instituto de Salud Publica de Chile | Instituto de Salud Publica de Chile |  | |
| EPI_ISL_276052 | EPI1127560 | B/Yam | Chile | 2017-Nov-06 | B/Iquique/90641/2017 | Instituto de Salud Publica de Chile | Instituto de Salud Publica de Chile |  | |
| EPI_ISL_276055 | EPI1127558 | B/Yam | Chile | 2017-Nov-10 | B/Osorno/90300/201 | Instituto de Salud Publica de Chile | Instituto de Salud Publica de Chile |  | |
| EPI_ISL_276056 | EPI1127507 | B/Yam | Chile | 2017-Nov-27 | B/Talca/90016/2017 | Instituto de Salud Publica de Chile | Instituto de Salud Publica de Chile |  | |
| EPI_ISL_276057 | EPI1129279 | B/Yam | Chile | 2017-Nov-22 | B/Santiago/89505/2017 | Instituto de Salud Publica de Chile | Instituto de Salud Publica de Chile |  | |
| EPI_ISL_276058 | EPI1127433 | B/Yam | Chile | 2017-Nov-10 | B/Temuco/89447/2017 | Instituto de Salud Publica de Chile | Instituto de Salud Publica de Chile |  | |
| EPI_ISL_276550 | EPI1127431 | B/Yam | Chile | 2017-Nov-02 | B/SanAntonio/89187/2017 | Instituto de Salud Publica de Chile | Instituto de Salud Publica de Chile |  | |
| EPI_ISL_276551 | EPI1108265 | B/Yam | Chile | 2017-Aug-07 | B/PuntaArenas/63332/2017 | Instituto de Salud Publica de Chile | Instituto de Salud Publica de Chile |  | |
| EPI_ISL_276552 | EPI1108263 | B/Yam | Chile | 2017-Aug-11 | B/VinadelMar/62111/2017 | Instituto de Salud Publica de Chile | Instituto de Salud Publica de Chile |  | |
| EPI_ISL_276553 | EPI1108261 | B/Yam | Chile | 2017-Aug-10 | B/Santiago/61643/2017 | Instituto de Salud Publica de Chile | Instituto de Salud Publica de Chile |  | |
| EPI_ISL_276554 | EPI1108259 | B/Yam | Chile | 2017-Aug-03 | B/Rancagua/61641/2017 | Instituto de Salud Publica de Chile | Instituto de Salud Publica de Chile |  | |
| EPI_ISL_276555 | EPI1108257 | B/Yam | Chile | 2017-Jul-31 | B/VinadelMar/60832/2017 | Instituto de Salud Publica de Chile | Instituto de Salud Publica de Chile |  | |
| EPI_ISL_276556 | EPI1108158 | B/Yam | Chile | 2017-Aug-03 | B/Concepcion/60377/2017 | Instituto de Salud Publica de Chile | Instituto de Salud Publica de Chile |  | |
| EPI_ISL_276557 | EPI1108156 | B/Yam | Chile | 2017-Aug-04 | B/Talca/59846/2017 | Instituto de Salud Publica de Chile | Instituto de Salud Publica de Chile |  | |
| EPI_ISL_276558 | EPI1108152 | B/Yam | Chile | 2017-Aug-02 | B/Santiago/59264/2017 | Instituto de Salud Publica de Chile | Instituto de Salud Publica de Chile |  | |
| EPI_ISL_276559 | EPI1081433 | B/Yam | Chile | 2017-Jul-24 | B/Santiago/57042/2017 | Instituto de Salud Publica de Chile | Instituto de Salud Publica de Chile |  | |
| EPI_ISL_277223 | EPI1081427 | B/Yam | Chile | 2017-Jul-12 | B/VinadelMar/55135/2017 | Instituto de Salud Publica de Chile | Instituto de Salud Publica de Chile |  | |
| EPI_ISL_282020 | EPI1081425 | B/Yam | Chile | 2017-Jul-18 | B/Santiago/55087/2017 | Instituto de Salud Publica de Chile | Instituto de Salud Publica de Chile |  | |
| EPI_ISL_282021 | EPI1049951 | B/Yam | Chile | 2017-Jun-21 | B/Santiago/47994/2017 | Instituto de Salud Publica de Chile | Instituto de Salud Publica de Chile |  | |
| EPI_ISL_282024 | EPI1049949 | B/Yam | Chile | 2017-Jun-16 | B/Rancagua/47545/2017 | Instituto de Salud Publica de Chile | Instituto de Salud Publica de Chile |  | |
| EPI_ISL_286282 | EPI1049947 | B/Yam | Chile | 2017-Jun-26 | B/Santiago/47381/2017 | Instituto de Salud Publica de Chile | Instituto de Salud Publica de Chile |  | |
| EPI_ISL_286284 | EPI1049945 | B/Yam | Chile | 2017-Jun-14 | B/Santiago/46344/2017 | Instituto de Salud Publica de Chile | Instituto de Salud Publica de Chile |  | |
| EPI_ISL_286285 | EPI1049939 | B/Yam | Chile | 2017-Jun-09 | B/Valparaiso/44595/2017 | Instituto de Salud Publica de Chile | Instituto de Salud Publica de Chile |  | |
| EPI_ISL_286312 | EPI1049937 | B/Yam | Chile | 2017-May-13 | B/Iquique/43908/2017 | Instituto de Salud Publica de Chile | Instituto de Salud Publica de Chile |  | |
| EPI_ISL_286313 | EPI1023702 | B/Yam | Chile | 2017-May-23 | B/Santiago/40847/2017 | Instituto de Salud Publica de Chile | Instituto de Salud Publica de Chile |  | |
| EPI_ISL_286314 | EPI1023699 | B/Yam | Chile | 2017-May-29 | B/Santiago/39225/2017 | Instituto de Salud Publica de Chile | Instituto de Salud Publica de Chile |  | |
| EPI_ISL_286315 | EPI1137989 | B/Yam | Chile | 2017-Nov-07 | B/Punta Arenas/84895/2017 | Instituto de Salud Publica de Chile | Centers for Disease Control and Prevention |  | |
| EPI_ISL_286316 | EPI1137981 | B/Yam | Chile | 2017-Oct-19 | B/Puerto Montt/81437/2017 | Instituto de Salud Publica de Chile | Centers for Disease Control and Prevention |  | |
| EPI_ISL_289157 | EPI1137973 | B/Yam | Chile | 2017-Oct-25 | B/Santiago/81374/2017 | Instituto de Salud Publica de Chile | Centers for Disease Control and Prevention |  | |
| EPI_ISL_289158 | EPI1137965 | B/Yam | Chile | 2017-Oct-23 | B/Puerto Montt/81288/2017 | Instituto de Salud Publica de Chile | Centers for Disease Control and Prevention |  | |
| EPI_ISL_289159 | EPI1137957 | B/Yam | Chile | 2017-Oct-23 | B/Coyhaique/80788/2017 | Instituto de Salud Publica de Chile | Centers for Disease Control and Prevention |  | |
| EPI_ISL_289169 | EPI1137949 | B/Yam | Chile | 2017-Oct-23 | B/La Serena/80547/2017 | Instituto de Salud Publica de Chile | Centers for Disease Control and Prevention |  | |
| EPI_ISL_289179 | EPI1137941 | B/Yam | Chile | 2017-Oct-20 | B/Santiago/80166/2017 | Instituto de Salud Publica de Chile | Centers for Disease Control and Prevention |  | |
| EPI_ISL_289180 | EPI1137933 | B/Yam | Chile | 2017-Oct-16 | B/Santiago/80145/2017 | Instituto de Salud Publica de Chile | Centers for Disease Control and Prevention |  | |
| EPI_ISL_289181 | EPI1137925 | B/Yam | Chile | 2017-Oct-06 | B/Valdivia/79899/2017 | Instituto de Salud Publica de Chile | Centers for Disease Control and Prevention |  | |
| EPI_ISL_289182 | EPI1137917 | B/Yam | Chile | 2017-Oct-16 | B/Curico/79752/2017 | Instituto de Salud Publica de Chile | Centers for Disease Control and Prevention |  | |
| EPI_ISL_289183 | EPI1137909 | B/Yam | Chile | 2017-Oct-16 | B/Rancagua/79691/2017 | Instituto de Salud Publica de Chile | Centers for Disease Control and Prevention |  | |
| EPI_ISL_290834 | EPI1137901 | B/Yam | Chile | 2017-Oct-11 | B/Punta Arenas/78700/2017 | Instituto de Salud Publica de Chile | Centers for Disease Control and Prevention |  | |
| EPI_ISL_290835 | EPI1137893 | B/Yam | Chile | 2017-Oct-08 | B/Concepcion/78349/2017 | Instituto de Salud Publica de Chile | Centers for Disease Control and Prevention |  | |
| EPI_ISL_290836 | EPI1137885 | B/Yam | Chile | 2017-Oct-17 | B/Santiago/78341/2017 | Instituto de Salud Publica de Chile | Centers for Disease Control and Prevention |  | |
| EPI_ISL_290837 | EPI1137877 | B/Yam | Chile | 2017-Oct-12 | B/Talca/78092/2017 | Instituto de Salud Publica de Chile | Centers for Disease Control and Prevention |  | |
| EPI_ISL_290838 | EPI1137869 | B/Yam | Chile | 2017-Sep-12 | B/Valdivia/77617/2017 | Instituto de Salud Publica de Chile | Centers for Disease Control and Prevention |  | |
| EPI_ISL_290839 | EPI1137861 | B/Yam | Chile | 2017-Oct-09 | B/Santiago/76319/2017 | Instituto de Salud Publica de Chile | Centers for Disease Control and Prevention |  | |
| EPI_ISL_290840 | EPI1137853 | B/Yam | Chile | 2017-Oct-04 | B/Talca/75976/2017 | Instituto de Salud Publica de Chile | Centers for Disease Control and Prevention |  | |
| EPI_ISL_290841 | EPI1137845 | B/Yam | Chile | 2017-Oct-04 | B/Santiago/75456/2017 | Instituto de Salud Publica de Chile | Centers for Disease Control and Prevention |  | |
| EPI_ISL_290842 | EPI1137837 | B/Yam | Chile | 2017-Oct-02 | B/Santiago/75265/2017 | Instituto de Salud Publica de Chile | Centers for Disease Control and Prevention |  | |
| EPI_ISL_290843 | EPI1137829 | B/Yam | Chile | 2017-Sep-16 | B/Osorno/73742/2017 | Instituto de Salud Publica de Chile | Centers for Disease Control and Prevention |  | |
| EPI_ISL_290844 | EPI1137821 | B/Yam | Chile | 2017-Sep-23 | B/Vina Del Mar/73490/2017 | Instituto de Salud Publica de Chile | Centers for Disease Control and Prevention |  | |
| EPI_ISL_290845 | EPI1056577 | B/Yam | Chile | 2017-Jul-27 | B/Santiago/57867/2017 | Instituto de Salud Publica de Chile | Centers for Disease Control and Prevention |  | |
| EPI_ISL_290846 | EPI1052808 | B/Yam | Chile | 2017-Jul-23 | B/Concepcion/57850/2017 | Instituto de Salud Publica de Chile | Centers for Disease Control and Prevention |  | |
| EPI_ISL_290847 | EPI1052800 | B/Yam | Chile | 2017-Jul-15 | B/Iquique/57718/2017 | Instituto de Salud Publica de Chile | Centers for Disease Control and Prevention |  | |
| EPI_ISL_290848 | EPI1052792 | B/Yam | Chile | 2017-Jul-24 | B/Cerro Navia/57042/2017 | Instituto de Salud Publica de Chile | Centers for Disease Control and Prevention |  | |
| EPI_ISL_290849 | EPI1052784 | B/Yam | Chile | 2017-Jul-04 | B/Valdivia/55250/2017 | Instituto de Salud Publica de Chile | Centers for Disease Control and Prevention |  | |
| EPI_ISL_290850 | EPI1052776 | B/Yam | Chile | 2017-Jul-18 | B/Santiago/55161/2017 | Instituto de Salud Publica de Chile | Centers for Disease Control and Prevention |  | |
| EPI_ISL_290851 | EPI1052768 | B/Yam | Chile | 2017-Jul-13 | B/Rancagua/55148/2017 | Instituto de Salud Publica de Chile | Centers for Disease Control and Prevention |  | |
| EPI_ISL_290852 | EPI1052760 | B/Yam | Chile | 2017-Jul-12 | B/Vina Del Mar/55135/2017 | Instituto de Salud Publica de Chile | Centers for Disease Control and Prevention |  | |
| EPI_ISL_290853 | EPI1052752 | B/Yam | Chile | 2017-Jul-18 | B/San Ramon/55087/2017 | Instituto de Salud Publica de Chile | Centers for Disease Control and Prevention |  | |
| EPI_ISL_290854 | EPI1052744 | B/Yam | Chile | 2017-Jul-10 | B/Santiago/53524/2017 | Instituto de Salud Publica de Chile | Centers for Disease Control and Prevention |  | |
| EPI_ISL_290855 | EPI1052736 | B/Yam | Chile | 2017-Jul-12 | B/Santiago/53521/2017 | Instituto de Salud Publica de Chile | Centers for Disease Control and Prevention |  | |
| EPI_ISL_290856 | EPI1256994 | B/Yam | Chile | 2018-Jan-12 | B/Valparaiso/11158/2018 | Instituto de Salud Publica de Chile | Instituto de Salud Publica de Chile | Tognarelli J, Lagos J, Arata L, Fasce R, Fernandez J | |
| EPI_ISL_290857 | EPI1256980 | B/Yam | Chile | 2018-Feb-02 | B/Talca/9908/2018 | Instituto de Salud Publica de Chile | Instituto de Salud Publica de Chile | Tognarelli J, Lagos J, Arata L, Fasce R, Fernandez J | |
| EPI_ISL_304455 | EPI1256971 | B/Yam | Chile | 2018-Jan-28 | B/Santiago/9537/2018 | Instituto de Salud Publica de Chile | Instituto de Salud Publica de Chile | Tognarelli J, Lagos J, Arata L, Fasce R, Fernandez J | |
| EPI_ISL_304456 | EPI1256959 | B/Yam | Chile | 2018-Jan-23 | B/Santiago/7336/2018 | Instituto de Salud Publica de Chile | Instituto de Salud Publica de Chile | Tognarelli J, Lagos J, Arata L, Fasce R, Fernandez J | |
| EPI_ISL_304458 | EPI1256947 | B/Yam | Chile | 2018-Jan-19 | B/Punta_Arenas/6740/2018 | Instituto de Salud Publica de Chile | Instituto de Salud Publica de Chile | Tognarelli J, Lagos J, Arata L, Fasce R, Fernandez J | |
| EPI_ISL_304460 | EPI1256933 | B/Yam | Chile | 2018-Jan-07 | B/Iquique/5136/2018 | Instituto de Salud Publica de Chile | Instituto de Salud Publica de Chile | Tognarelli J, Lagos J, Arata L, Fasce R, Fernandez J | |
| EPI_ISL_304462 | EPI1256923 | B/Yam | Chile | 2017-Nov-28 | B/San_Antonio/4756/2018 | Instituto de Salud Publica de Chile | Instituto de Salud Publica de Chile | Tognarelli J, Lagos J, Arata L, Fasce R, Fernandez J | |
| EPI_ISL_304464 | EPI1256912 | B/Yam | Chile | 2017-Nov-08 | B/San_Antonio/4755/2018 | Instituto de Salud Publica de Chile | Instituto de Salud Publica de Chile | Tognarelli J, Lagos J, Arata L, Fasce R, Fernandez J | |
| EPI_ISL_304466 | EPI1256899 | B/Yam | Chile | 2017-Nov-04 | B/San_Antonio/4754/2018 | Instituto de Salud Publica de Chile | Instituto de Salud Publica de Chile | Tognarelli J, Lagos J, Arata L, Fasce R, Fernandez J | |
| EPI_ISL_304468 | EPI1256889 | B/Yam | Chile | 2017-Nov-08 | B/San_Antonio/4753/2018 | Instituto de Salud Publica de Chile | Instituto de Salud Publica de Chile | Tognarelli J, Lagos J, Arata L, Fasce R, Fernandez J | |
| EPI_ISL_304470 | EPI1256875 | B/Yam | Chile | 2017-Dec-13 | B/Valparaiso/4690/2018 | Instituto de Salud Publica de Chile | Instituto de Salud Publica de Chile | Tognarelli J, Lagos J, Arata L, Fasce R, Fernandez J | |
| EPI_ISL_304472 | EPI1256864 | B/Yam | Chile | 2018-Jan-04 | B/Concepcion/4568/2018 | Instituto de Salud Publica de Chile | Instituto de Salud Publica de Chile | Tognarelli J, Lagos J, Arata L, Fasce R, Fernandez J | |
| EPI_ISL_304474 | EPI1256851 | B/Yam | Chile | 2018-Jan-04 | B/Concepcion/4567/2018 | Instituto de Salud Publica de Chile | Instituto de Salud Publica de Chile | Tognarelli J, Lagos J, Arata L, Fasce R, Fernandez J | |
| EPI_ISL_304475 | EPI1256836 | B/Yam | Chile | 2017-Dec-24 | B/Concepcion/4566/2018 | Instituto de Salud Publica de Chile | Instituto de Salud Publica de Chile | Tognarelli J, Lagos J, Arata L, Fasce R, Fernandez J | |
| EPI_ISL_304477 | EPI1256828 | B/Yam | Chile | 2017-Dec-21 | B/Concepcion/4565/2018 | Instituto de Salud Publica de Chile | Instituto de Salud Publica de Chile | Tognarelli J, Lagos J, Arata L, Fasce R, Fernandez J | |
| EPI_ISL_304479 | EPI1256820 | B/Yam | Chile | 2018-Jan-15 | B/Santiago/3827/2018 | Instituto de Salud Publica de Chile | Instituto de Salud Publica de Chile | Tognarelli J, Lagos J, Arata L, Fasce R, Fernandez J | |
| EPI_ISL_304480 | EPI1256812 | B/Yam | Chile | 2018-Jan-03 | B/Osorno/3377/2018 | Instituto de Salud Publica de Chile | Instituto de Salud Publica de Chile | Tognarelli J, Lagos J, Arata L, Fasce R, Fernandez J | |
| EPI_ISL_304481 | EPI1256804 | B/Yam | Chile | 2018-Jan-03 | B/Osorno/3376/2018 | Instituto de Salud Publica de Chile | Instituto de Salud Publica de Chile | Tognarelli J, Lagos J, Arata L, Fasce R, Fernandez J | |
| EPI_ISL_304482 | EPI1256796 | B/Yam | Chile | 2018-Jan-10 | B/Santiago/3372/2018 | Instituto de Salud Publica de Chile | Instituto de Salud Publica de Chile | Tognarelli J, Lagos J, Arata L, Fasce R, Fernandez J | |
| EPI_ISL_314441 | EPI1256788 | B/Yam | Chile | 2018-Jan-09 | B/Santiago/3371/2018 | Instituto de Salud Publica de Chile | Instituto de Salud Publica de Chile | Tognarelli J, Lagos J, Arata L, Fasce R, Fernandez J | |
| EPI_ISL_314442 | EPI1256780 | B/Yam | Chile | 2018-Jan-08 | B/Valparaiso/2762/2018 | Instituto de Salud Publica de Chile | Instituto de Salud Publica de Chile | Tognarelli J, Lagos J, Arata L, Fasce R, Fernandez J | |
| EPI_ISL_314443 | EPI1256772 | B/Yam | Chile | 2017-Dec-12 | B/Iquique/1309/2018 | Instituto de Salud Publica de Chile | Instituto de Salud Publica de Chile | Tognarelli J, Lagos J, Arata L, Fasce R, Fernandez J | |
| EPI_ISL_314444 | EPI1256756 | B/Yam | Chile | 2018-Jan-01 | B/Valparaiso/957/2018 | Instituto de Salud Publica de Chile | Instituto de Salud Publica de Chile | Tognarelli J, Lagos J, Arata L, Fasce R, Fernandez J | |
| EPI_ISL_314445 | EPI1256748 | B/Yam | Chile | 2017-Dec-26 | B/Puerto_Montt/690/2018 | Instituto de Salud Publica de Chile | Instituto de Salud Publica de Chile | Tognarelli J, Lagos J, Arata L, Fasce R, Fernandez J | |
| EPI_ISL_314446 | EPI1256740 | B/Yam | Chile | 2017-Dec-26 | B/Puerto_Montt/327/2018 | Instituto de Salud Publica de Chile | Instituto de Salud Publica de Chile | Tognarelli J, Lagos J, Arata L, Fasce R, Fernandez J | |
| EPI_ISL_314840 | EPI1256732 | B/Yam | Chile | 2017-Dec-27 | B/Santiago/164/2018 | Instituto de Salud Publica de Chile | Instituto de Salud Publica de Chile | Tognarelli J, Lagos J, Arata L, Fasce R, Fernandez J | |
| EPI_ISL_314841 | EPI1256724 | B/Yam | Chile | 2017-Dec-26 | B/Santiago/163/2018 | Instituto de Salud Publica de Chile | Instituto de Salud Publica de Chile | Tognarelli J, Lagos J, Arata L, Fasce R, Fernandez J | |
| EPI_ISL_314844 | EPI1255893 | B/Yam | Chile | 2018-Feb-17 | B/SanAntonio/35580/2018 | Instituto de Salud Publica de Chile | Instituto de Salud Publica de Chile |  | |
| EPI_ISL_315051 | EPI1255887 | B/Yam | Chile | 2018-Mar-24 | B/Santiago/25798/2018 | Instituto de Salud Publica de Chile | Instituto de Salud Publica de Chile |  | |
| EPI_ISL_315052 | EPI1255885 | B/Yam | Chile | 2018-Mar-08 | B/Antofagasta/21482/2018 | Instituto de Salud Publica de Chile | Instituto de Salud Publica de Chile |  | |
| EPI_ISL_315053 | EPI1202900 | B/Yam | Chile | 2018-Feb-06 | B/LaSerena/11709/2018 | Instituto de Salud Publica de Chile | Instituto de Salud Publica de Chile |  | |
| EPI_ISL_315054 | EPI1202898 | B/Yam | Chile | 2018-Jan-19 | B/PuntaArena/6740/2018 | Instituto de Salud Publica de Chile | Instituto de Salud Publica de Chile |  | |
| EPI_ISL_315055 | EPI1202896 | B/Yam | Chile | 2017-Nov-08 | B/SanAntonio/4753/2017 | Instituto de Salud Publica de Chile | Instituto de Salud Publica de Chile |  | |
| EPI_ISL_315057 | EPI1202894 | B/Yam | Chile | 2017-Dec-21 | B/Concepcion/4565/2017 | Instituto de Salud Publica de Chile | Instituto de Salud Publica de Chile |  | |
| EPI_ISL_315058 | EPI1202890 | B/Yam | Chile | 2017-Dec-12 | B/Iquique/1309/2017 | Instituto de Salud Publica de Chile | Instituto de Salud Publica de Chile |  | |
| EPI_ISL_315059 | EPI1202886 | B/Yam | Chile | 2017-Dec-26 | B/PuertoMontt/327/2017 | Instituto de Salud Publica de Chile | Instituto de Salud Publica de Chile |  | |
| EPI_ISL_315060 | EPI1202884 | B/Yam | Chile | 2017-Nov-05 | B/SanFelipe/97130/2017 | Instituto de Salud Publica de Chile | Instituto de Salud Publica de Chile |  | |
| EPI_ISL_315061 | EPI1202880 | B/Yam | Chile | 2017-Dec-15 | B/Santiago/97812/2017 | Instituto de Salud Publica de Chile | Instituto de Salud Publica de Chile |  | |
| EPI_ISL_315062 | EPI1202876 | B/Yam | Chile | 2017-Dec-17 | B/Santiago/97811/2017 | Instituto de Salud Publica de Chile | Instituto de Salud Publica de Chile |  | |
| EPI_ISL_315063 | EPI1202872 | B/Yam | Chile | 2017-Dec-06 | B/Concepcion/96591/2017 | Instituto de Salud Publica de Chile | Instituto de Salud Publica de Chile |  | |
| EPI_ISL_315064 | EPI1202868 | B/Yam | Chile | 2017-Dec-08 | B/PuertoMontt/95507/2017 | Instituto de Salud Publica de Chile | Instituto de Salud Publica de Chile |  | |
| EPI_ISL_315065 | EPI1202864 | B/Yam | Chile | 2017-Nov-14 | B/PuertoMontt/91245/2017 | Instituto de Salud Publica de Chile | Instituto de Salud Publica de Chile |  | |
| EPI_ISL_315067 | EPI1202860 | B/Yam | Chile | 2017-Nov-22 | B/Santiago/89003/2017 | Instituto de Salud Publica de Chile | Instituto de Salud Publica de Chile |  | |
| EPI_ISL_315068 | EPI1202856 | B/Yam | Chile | 2017-Oct-11 | B/Santiago/80143/2017 | Instituto de Salud Publica de Chile | Instituto de Salud Publica de Chile |  | |
| EPI_ISL_315070 | EPI1202852 | B/Yam | Chile | 2017-Sep-15 | B/Santiago/71256/2017 | Instituto de Salud Publica de Chile | Instituto de Salud Publica de Chile |  | |
| EPI_ISL_315071 | EPI1202848 | B/Yam | Chile | 2017-Jul-14 | B/Santiago/56163/2017 | Instituto de Salud Publica de Chile | Instituto de Salud Publica de Chile |  | |
| EPI_ISL_315073 | EPI1202837 | B/Yam | Chile | 2017-Jun-30 | B/Santiago/50362/2017 | Instituto de Salud Publica de Chile | Instituto de Salud Publica de Chile |  | |
| EPI_ISL_315074 | EPI1273442 | B/Yam | Chile | 2018-Jul-06 | B/Copiapo/58962/2018 | Instituto de Salud Publica de Chile | Centers for Disease Control and Prevention |  | |
| EPI_ISL_315076 | EPI1273434 | B/Yam | Chile | 2018-Jun-27 | B/Valparaiso/54262/2018 | Instituto de Salud Publica de Chile | Centers for Disease Control and Prevention |  | |
| EPI_ISL_315077 | EPI1273386 | B/Yam | Chile | 2018-Jun-24 | B/Vina Del Mar/52468/2018 | Instituto de Salud Publica de Chile | Centers for Disease Control and Prevention |  | |
| EPI_ISL_315079 | EPI1273314 | B/Yam | Chile | 2018-Jul-21 | B/La Serena/60407/2018 | Instituto de Salud Publica de Chile | Centers for Disease Control and Prevention |  | |
| EPI_ISL_315080 | EPI1261287 | B/Yam | Chile | 2018-Apr-23 | B/Santiago/34517/2018 | Instituto de Salud Publica de Chile | Centers for Disease Control and Prevention |  | |
| EPI_ISL_315082 | EPI1261234 | B/Yam | Chile | 2018-May-16 | B/Santiago/39846/2018 | Instituto de Salud Publica de Chile | Centers for Disease Control and Prevention |  | |
| EPI_ISL_315083 | EPI1254003 | B/Yam | Chile | 2018-May-04 | B/Vinadelmar/29110/2018 | Instituto de Salud Publica de Chile | Centers for Disease Control and Prevention |  | |
| EPI_ISL_315085 | EPI1253995 | B/Yam | Chile | 2018-Apr-09 | B/Santiago/30340/2018 | Instituto de Salud Publica de Chile | Centers for Disease Control and Prevention |  | |
| EPI_ISL_316477 | EPI1253988 | B/Yam | Chile | 2018-Apr-12 | B/Laserena/31038/2018 | Instituto de Salud Publica de Chile | Centers for Disease Control and Prevention |  | |
| EPI_ISL_316484 | EPI1253980 | B/Yam | Chile | 2018-Apr-19 | B/Laserena/33170/2018 | Instituto de Salud Publica de Chile | Centers for Disease Control and Prevention |  | |
| EPI_ISL_321152 | EPI1253972 | B/Yam | Chile | 2018-Apr-12 | B/Sanantonio/35578/2018 | Instituto de Salud Publica de Chile | Centers for Disease Control and Prevention |  | |
| EPI_ISL_321161 | EPI1253964 | B/Yam | Chile | 2018-Apr-27 | B/Valparaiso/35671/2018 | Instituto de Salud Publica de Chile | Centers for Disease Control and Prevention |  | |
| EPI_ISL_321167 | EPI1138005 | B/Yam | Chile | 2017-Oct-25 | B/Concepcion/85156/2017 | Instituto de Salud Publica de Chile | Centers for Disease Control and Prevention |  | |
| EPI_ISL_321168 | EPI1137997 | B/Yam | Chile | 2017-Oct-30 | B/Puerto Montt/85126/2017 | Instituto de Salud Publica de Chile | Centers for Disease Control and Prevention |  | |
| EPI_ISL_321169 | EPI1273450 | B/Yam | Chile | 2018-Jun-21 | B/Concepcion/52325/2018 | Instituto de Salud Publica de Chile | Centers for Disease Control and Prevention |  | |
| EPI_ISL_321181 | EPI1273546 | B/Yam | Chile | 2018-Jun-08 | B/Osorno/47366/2018 | Instituto de Salud Publica de Chile | Centers for Disease Control and Prevention |  | |
| EPI_ISL_321185 | EPI1273578 | B/Yam | Chile | 2018-Jun-27 | B/Santiago/53060/2018 | Instituto de Salud Publica de Chile | Centers for Disease Control and Prevention |  | |
| EPI_ISL_322131 | EPI1279021 | B/Yam | Chile | 2018-May-17 | B/Santiago/40365/2018 | Instituto de Salud Publica de Chile | Instituto de Salud Publica de Chile | Tognarelli J, Lagos J, Arata L, Fasce R, Fernandez J | |
| EPI_ISL_322132 | EPI1279029 | B/Yam | Chile | 2018-May-14 | B/Iquique/44551/2018 | Instituto de Salud Publica de Chile | Instituto de Salud Publica de Chile | Tognarelli J, Lagos J, Arata L, Fasce R, Fernandez J | |
| EPI_ISL_312915 | EPI1282554 | A/H1pdm09 | Colombia | 2018-Jun-28 | A/Colombia/9827/2018 | Instituto Nacional de Salud de Columbia | Centers for Disease Control and Prevention |  | |
| EPI_ISL_312916 | EPI1282546 | A/H1pdm09 | Colombia | 2018-Jun-28 | A/Colombia/9829/2018 | Instituto Nacional de Salud de Columbia | Centers for Disease Control and Prevention |  | |
| EPI_ISL_312917 | EPI1282538 | A/H1pdm09 | Colombia | 2018-Jun-26 | A/Colombia/9833/2018 | Instituto Nacional de Salud de Columbia | Centers for Disease Control and Prevention |  | |
| EPI_ISL_312918 | EPI1282530 | A/H1pdm09 | Colombia | 2018-Jul-11 | A/Colombia/0103/2018 | Instituto Nacional de Salud de Columbia | Centers for Disease Control and Prevention |  | |
| EPI_ISL_312919 | EPI1282522 | A/H1pdm09 | Colombia | 2018-Jul-01 | A/Colombia/0277/2018 | Instituto Nacional de Salud de Columbia | Centers for Disease Control and Prevention |  | |
| EPI_ISL_312920 | EPI1282506 | A/H1pdm09 | Colombia | 2018-Jul-12 | A/Colombia/0358/2018 | Instituto Nacional de Salud de Columbia | Centers for Disease Control and Prevention |  | |
| EPI_ISL_312921 | EPI1282498 | A/H1pdm09 | Colombia | 2018-Jun-25 | A/Colombia/0369/2018 | Instituto Nacional de Salud de Columbia | Centers for Disease Control and Prevention |  | |
| EPI_ISL_312922 | EPI1282490 | A/H1pdm09 | Colombia | 2018-Jul-03 | A/Colombia/0389/2018 | Instituto Nacional de Salud de Columbia | Centers for Disease Control and Prevention |  | |
| EPI_ISL_312923 | EPI1282474 | A/H1pdm09 | Colombia | 2018-Jul-08 | A/Colombia/0465/2018 | Instituto Nacional de Salud de Columbia | Centers for Disease Control and Prevention |  | |
| EPI_ISL_312924 | EPI1282458 | A/H1pdm09 | Colombia | 2018-Jul-14 | A/Colombia/0540/2018 | Instituto Nacional de Salud de Columbia | Centers for Disease Control and Prevention |  | |
| EPI_ISL_312925 | EPI1282450 | A/H1pdm09 | Colombia | 2018-Jul-16 | A/Colombia/0544/2018 | Instituto Nacional de Salud de Columbia | Centers for Disease Control and Prevention |  | |
| EPI_ISL_322901 | EPI1282442 | A/H1pdm09 | Colombia | 2018-Jul-12 | A/Colombia/0559/2018 | Instituto Nacional de Salud de Columbia | Centers for Disease Control and Prevention |  | |
| EPI_ISL_322903 | EPI1282434 | A/H1pdm09 | Colombia | 2018-Jul-13 | A/Colombia/0602/2018 | Instituto Nacional de Salud de Columbia | Centers for Disease Control and Prevention |  | |
| EPI_ISL_322905 | EPI1282418 | A/H1pdm09 | Colombia | 2018-Jul-05 | A/Colombia/0638/2018 | Instituto Nacional de Salud de Columbia | Centers for Disease Control and Prevention |  | |
| EPI_ISL_322906 | EPI1282404 | A/H1pdm09 | Colombia | 2018-Jun-14 | A/Colombia/9414/2018 | Instituto Nacional de Salud de Columbia | Centers for Disease Control and Prevention |  | |
| EPI_ISL_322907 | EPI1245799 | A/H1pdm09 | Colombia | 2018-Mar-18 | A/Colombia/7888/2018 | Instituto Nacional de Salud de Columbia | Centers for Disease Control and Prevention |  | |
| EPI_ISL_322908 | EPI1245791 | A/H1pdm09 | Colombia | 2018-Mar-17 | A/Colombia/7886/2018 | Instituto Nacional de Salud de Columbia | Centers for Disease Control and Prevention |  | |
| EPI_ISL_322910 | EPI1245783 | A/H1pdm09 | Colombia | 2018-Mar-16 | A/Colombia/7884/2018 | Instituto Nacional de Salud de Columbia | Centers for Disease Control and Prevention |  | |
| EPI_ISL_322912 | EPI1245775 | A/H1pdm09 | Colombia | 2018-Mar-03 | A/Colombia/7741/2018 | Instituto Nacional de Salud de Columbia | Centers for Disease Control and Prevention |  | |
| EPI_ISL_322913 | EPI1245767 | A/H1pdm09 | Colombia | 2018-Mar-05 | A/Colombia/7735/2018 | Instituto Nacional de Salud de Columbia | Centers for Disease Control and Prevention |  | |
| EPI_ISL_322914 | EPI1245761 | A/H1pdm09 | Colombia | 2018-Apr-10 | A/Colombia/8173/2018 | Instituto Nacional de Salud de Columbia | Centers for Disease Control and Prevention |  | |
| EPI_ISL_322916 | EPI1245753 | A/H1pdm09 | Colombia | 2018-Apr-06 | A/Colombia/8171/2018 | Instituto Nacional de Salud de Columbia | Centers for Disease Control and Prevention |  | |
| EPI_ISL_322917 | EPI1245745 | A/H1pdm09 | Colombia | 2018-Apr-05 | A/Colombia/8170/2018 | Instituto Nacional de Salud de Columbia | Centers for Disease Control and Prevention |  | |
| EPI_ISL_322918 | EPI1245737 | A/H1pdm09 | Colombia | 2018-Mar-23 | A/Colombia/8089/2018 | Instituto Nacional de Salud de Columbia | Centers for Disease Control and Prevention |  | |
| EPI_ISL_322919 | EPI1245729 | A/H1pdm09 | Colombia | 2018-Apr-15 | A/Colombia/8237/2018 | Instituto Nacional de Salud de Columbia | Centers for Disease Control and Prevention |  | |
| EPI_ISL_322920 | EPI1245721 | A/H1pdm09 | Colombia | 2018-Apr-14 | A/Colombia/8233/2018 | Instituto Nacional de Salud de Columbia | Centers for Disease Control and Prevention |  | |
| EPI_ISL_268382 | EPI1282822 | A/H3 | Colombia | 2018-Jul-04 | A/Colombia/9948/2018 | Instituto Nacional de Salud de Columbia | Centers for Disease Control and Prevention |  | |
| EPI_ISL_268383 | EPI1282814 | A/H3 | Colombia | 2018-Jun-27 | A/Colombia/0113/2018 | Instituto Nacional de Salud de Columbia | Centers for Disease Control and Prevention |  | |
| EPI_ISL_268385 | EPI1282806 | A/H3 | Colombia | 2018-Jul-18 | A/Colombia/0297/2018 | Instituto Nacional de Salud de Columbia | Centers for Disease Control and Prevention |  | |
| EPI_ISL_268386 | EPI1282802 | A/H3 | Colombia | 2018-Jul-13 | A/Colombia/0291/2018 | Instituto Nacional de Salud de Columbia | Centers for Disease Control and Prevention |  | |
| EPI_ISL_277493 | EPI1249057 | A/H3 | Colombia | 2018-Apr-06 | A/Colombia/8139/2018 | Instituto Nacional de Salud de Columbia | Centers for Disease Control and Prevention |  | |
| EPI_ISL_277494 | EPI1163391 | A/H3 | Colombia | 2017-Oct-01 | A/Colombia/6525/2017 | Instituto Nacional de Salud de Columbia | Centers for Disease Control and Prevention |  | |
| EPI_ISL_277495 | EPI1163383 | A/H3 | Colombia | 2017-Nov-12 | A/Colombia/6446/2017 | Instituto Nacional de Salud de Columbia | Centers for Disease Control and Prevention |  | |
| EPI_ISL_277496 | EPI1163375 | A/H3 | Colombia | 2017-Nov-01 | A/Colombia/6434/2017 | Instituto Nacional de Salud de Columbia | Centers for Disease Control and Prevention |  | |
| EPI_ISL_277497 | EPI1163367 | A/H3 | Colombia | 2017-Oct-27 | A/Colombia/6380/2017 | Instituto Nacional de Salud de Columbia | Centers for Disease Control and Prevention |  | |
| EPI_ISL_277498 | EPI1163359 | A/H3 | Colombia | 2017-Oct-20 | A/Colombia/6187/2017 | Instituto Nacional de Salud de Columbia | Centers for Disease Control and Prevention |  | |
| EPI_ISL_277499 | EPI1163351 | A/H3 | Colombia | 2017-Oct-20 | A/Colombia/6186/2017 | Instituto Nacional de Salud de Columbia | Centers for Disease Control and Prevention |  | |
| EPI_ISL_277500 | EPI1166513 | A/H3 | Colombia | 2017-Oct-05 | A/Colombia/6062/2017 | Instituto Nacional de Salud de Columbia | Centers for Disease Control and Prevention |  | |
| EPI_ISL_277501 | EPI1161687 | A/H3 | Colombia | 2017-Dec-04 | A/Colombia/6741/2017 | Instituto Nacional de Salud de Columbia | Centers for Disease Control and Prevention |  | |
| EPI_ISL_277502 | EPI1161679 | A/H3 | Colombia | 2017-Dec-04 | A/Colombia/6740/2017 | Instituto Nacional de Salud de Columbia | Centers for Disease Control and Prevention |  | |
| EPI_ISL_277503 | EPI1161671 | A/H3 | Colombia | 2017-Dec-04 | A/Colombia/6739/2017 | Instituto Nacional de Salud de Columbia | Centers for Disease Control and Prevention |  | |
| EPI_ISL_277504 | EPI1161663 | A/H3 | Colombia | 2017-Dec-02 | A/Colombia/6717/2017 | Instituto Nacional de Salud de Columbia | Centers for Disease Control and Prevention |  | |
| EPI_ISL_277849 | EPI1161655 | A/H3 | Colombia | 2017-Nov-10 | A/Colombia/6646/2017 | Instituto Nacional de Salud de Columbia | Centers for Disease Control and Prevention |  | |
| EPI_ISL_292545 | EPI1161647 | A/H3 | Colombia | 2017-Nov-07 | A/Colombia/6639/2017 | Instituto Nacional de Salud de Columbia | Centers for Disease Control and Prevention |  | |
| EPI_ISL_292546 | EPI1146114 | A/H3 | Colombia | 2017-Oct-12 | A/Colombia/6063/2017 | Instituto Nacional de Salud de Columbia | Centers for Disease Control and Prevention |  | |
| EPI_ISL_295848 | EPI1146106 | A/H3 | Colombia | 2017-Oct-06 | A/Colombia/6059/2017 | Instituto Nacional de Salud de Columbia | Centers for Disease Control and Prevention |  | |
| EPI_ISL_295849 | EPI1059926 | A/H3 | Colombia | 2017-Jul-10 | A/Colombia/5158/2017 | Instituto Nacional de Salud de Columbia | Centers for Disease Control and Prevention |  | |
| EPI_ISL_295850 | EPI1058317 | A/H3 | Colombia | 2017-Aug-03 | A/Colombia/5238/2017 | Instituto Nacional de Salud de Columbia | Centers for Disease Control and Prevention |  | |
| EPI_ISL_295851 | EPI1058309 | A/H3 | Colombia | 2017-Jul-30 | A/Colombia/5207/2017 | Instituto Nacional de Salud de Columbia | Centers for Disease Control and Prevention |  | |
| EPI_ISL_295852 | EPI1058301 | A/H3 | Colombia | 2017-Jul-19 | A/Colombia/5174/2017 | Instituto Nacional de Salud de Columbia | Centers for Disease Control and Prevention |  | |
| EPI_ISL_295853 | EPI1058293 | A/H3 | Colombia | 2017-Jun-13 | A/Colombia/5133/2017 | Instituto Nacional de Salud de Columbia | Centers for Disease Control and Prevention |  | |
| EPI_ISL_296077 | EPI1058285 | A/H3 | Colombia | 2017-Jun-12 | A/Colombia/5131/2017 | Instituto Nacional de Salud de Columbia | Centers for Disease Control and Prevention |  | |
| EPI_ISL_296079 | EPI1058277 | A/H3 | Colombia | 2017-Jul-21 | A/Colombia/5121/2017 | Instituto Nacional de Salud de Columbia | Centers for Disease Control and Prevention |  | |
| EPI_ISL_296080 | EPI1058269 | A/H3 | Colombia | 2017-Jun-06 | A/Colombia/5118/2017 | Instituto Nacional de Salud de Columbia | Centers for Disease Control and Prevention |  | |
| EPI_ISL_296081 | EPI1058261 | A/H3 | Colombia | 2017-Jul-17 | A/Colombia/5099/2017 | Instituto Nacional de Salud de Columbia | Centers for Disease Control and Prevention |  | |
| EPI_ISL_296082 | EPI1058253 | A/H3 | Colombia | 2017-Jul-17 | A/Colombia/5071/2017 | Instituto Nacional de Salud de Columbia | Centers for Disease Control and Prevention |  | |
| EPI_ISL_296083 | EPI1058245 | A/H3 | Colombia | 2017-Jul-13 | A/Colombia/4979/2017 | Instituto Nacional de Salud de Columbia | Centers for Disease Control and Prevention |  | |
| EPI_ISL_296084 | EPI1058237 | A/H3 | Colombia | 2017-Jul-06 | A/Colombia/4923/2017 | Instituto Nacional de Salud de Columbia | Centers for Disease Control and Prevention |  | |
| EPI_ISL_313543 | EPI1058229 | A/H3 | Colombia | 2017-Jun-30 | A/Colombia/4830/2017 | Instituto Nacional de Salud de Columbia | Centers for Disease Control and Prevention |  | |
| EPI_ISL_322951 | EPI1017200 | A/H3 | Colombia | 2017-May-20 | A/Colombia/961/2017 | Instituto Nacional de Salud de Columbia | Centers for Disease Control and Prevention |  | |
| EPI_ISL_322952 | EPI1017192 | A/H3 | Colombia | 2017-May-16 | A/Colombia/960/2017 | Instituto Nacional de Salud de Columbia | Centers for Disease Control and Prevention |  | |
| EPI_ISL_322953 | EPI1017176 | A/H3 | Colombia | 2017-May-05 | A/Colombia/3875/2017 | Instituto Nacional de Salud de Columbia | Centers for Disease Control and Prevention |  | |
| EPI_ISL_322954 | EPI1017168 | A/H3 | Colombia | 2017-May-05 | A/Colombia/3846/2017 | Instituto Nacional de Salud de Columbia | Centers for Disease Control and Prevention |  | |
| EPI_ISL_277651 | EPI1059098 | B/Yam | Colombia | 2017-Jun-25 | B/Colombia/4799/2017 | Instituto Nacional de Salud de Columbia | Centers for Disease Control and Prevention |  | |
| EPI_ISL_277652 | EPI1059106 | B/Yam | Colombia | 2017-Jul-03 | B/Colombia/4831/2017 | Instituto Nacional de Salud de Columbia | Centers for Disease Control and Prevention |  | |
| EPI_ISL_277653 | EPI1059114 | B/Yam | Colombia | 2017-May-26 | B/Colombia/5117/2017 | Instituto Nacional de Salud de Columbia | Centers for Disease Control and Prevention |  | |
| EPI_ISL_277654 | EPI1059122 | B/Yam | Colombia | 2017-Jul-21 | B/Colombia/5199/2017 | Instituto Nacional de Salud de Columbia | Centers for Disease Control and Prevention |  | |
| EPI_ISL_277655 | EPI1059130 | B/Yam | Colombia | 2017-Jul-31 | B/Colombia/5202/2017 | Instituto Nacional de Salud de Columbia | Centers for Disease Control and Prevention |  | |
| EPI_ISL_277656 | EPI1059138 | B/Yam | Colombia | 2017-Jul-30 | B/Colombia/5237/2017 | Instituto Nacional de Salud de Columbia | Centers for Disease Control and Prevention |  | |
| EPI_ISL_292693 | EPI1147287 | B/Yam | Colombia | 2017-Oct-31 | B/Colombia/6457/2017 | Instituto Nacional de Salud de Columbia | Centers for Disease Control and Prevention |  | |
| EPI_ISL_292694 | EPI1147295 | B/Yam | Colombia | 2017-Nov-14 | B/Colombia/6543/2017 | Instituto Nacional de Salud de Columbia | Centers for Disease Control and Prevention |  | |
| EPI_ISL_313074 | EPI1246680 | B/Yam | Colombia | 2018-Mar-20 | B/Colombia/8093/2018 | Instituto Nacional de Salud de Columbia | Centers for Disease Control and Prevention |  | |
| EPI_ISL_313075 | EPI1246688 | B/Yam | Colombia | 2018-Mar-22 | B/Colombia/8172/2018 | Instituto Nacional de Salud de Columbia | Centers for Disease Control and Prevention |  | |
| EPI_ISL_313198 | EPI1247633 | B/Yam | Colombia | 2018-Mar-23 | B/Colombia/8036/2018 | Instituto Nacional de Salud de Columbia | Centers for Disease Control and Prevention |  | |
| EPI_ISL_313221 | EPI1247814 | B/Yam | Colombia | 2018-Mar-13 | B/Colombia/7901/2018 | Instituto Nacional de Salud de Columbia | Centers for Disease Control and Prevention |  | |
| EPI_ISL_322849 | EPI1281953 | B/Yam | Colombia | 2018-Jul-18 | B/Colombia/0603/2018 | Instituto Nacional de Salud de Columbia | Centers for Disease Control and Prevention |  | |
| EPI_ISL_322852 | EPI1281975 | B/Yam | Colombia | 2018-Jul-08 | B/Colombia/0485/2018 | Instituto Nacional de Salud de Columbia | Centers for Disease Control and Prevention |  | |
| EPI_ISL_322853 | EPI1281982 | B/Yam | Colombia | 2018-Jun-07 | B/Colombia/0011/2018 | Instituto Nacional de Salud de Columbia | Centers for Disease Control and Prevention |  | |
| EPI_ISL_282869 | EPI1166388 | A/H3 | Costa Rica | 2017-Nov-12 | A/Costa Rica/6973/2017 | Laboratorio Nacional de Influenza | Centers for Disease Control and Prevention |  | |
| EPI_ISL_282870 | EPI1166364 | A/H3 | Costa Rica | 2017-Nov-18 | A/Costa Rica/6947/2017 | Laboratorio Nacional de Influenza | Centers for Disease Control and Prevention |  | |
| EPI_ISL_282871 | EPI1166356 | A/H3 | Costa Rica | 2017-Nov-17 | A/Costa Rica/6886/2017 | Laboratorio Nacional de Influenza | Centers for Disease Control and Prevention |  | |
| EPI_ISL_282872 | EPI1166348 | A/H3 | Costa Rica | 2017-Nov-16 | A/Costa Rica/6885/2017 | Laboratorio Nacional de Influenza | Centers for Disease Control and Prevention |  | |
| EPI_ISL_282873 | EPI1166340 | A/H3 | Costa Rica | 2017-Nov-17 | A/Costa Rica/6871/2017 | Laboratorio Nacional de Influenza | Centers for Disease Control and Prevention |  | |
| EPI_ISL_282874 | EPI1166332 | A/H3 | Costa Rica | 2017-Nov-17 | A/Costa Rica/6850/2017 | Laboratorio Nacional de Influenza | Centers for Disease Control and Prevention |  | |
| EPI_ISL_282875 | EPI1166324 | A/H3 | Costa Rica | 2017-Nov-15 | A/Costa Rica/6788/2017 | Laboratorio Nacional de Influenza | Centers for Disease Control and Prevention |  | |
| EPI_ISL_296088 | EPI1166516 | A/H3 | Costa Rica | 2017-Nov-13 | A/Costa Rica/6568/2017 | Laboratorio Nacional de Influenza | Centers for Disease Control and Prevention |  | |
| EPI_ISL_296090 | EPI1166311 | A/H3 | Costa Rica | 2017-Nov-13 | A/Costa Rica/6556/2017 | Laboratorio Nacional de Influenza | Centers for Disease Control and Prevention |  | |
| EPI_ISL_296091 | EPI1165986 | A/H3 | Costa Rica | 2017-Nov-27 | A/Costa Rica/7481/2017 | Laboratorio Nacional de Influenza | Centers for Disease Control and Prevention |  | |
| EPI_ISL_296460 | EPI1165970 | A/H3 | Costa Rica | 2017-Nov-22 | A/Costa Rica/7256/2017 | Laboratorio Nacional de Influenza | Centers for Disease Control and Prevention |  | |
| EPI_ISL_296472 | EPI1165962 | A/H3 | Costa Rica | 2017-Nov-21 | A/Costa Rica/7181/2017 | Laboratorio Nacional de Influenza | Centers for Disease Control and Prevention |  | |
| EPI_ISL_296473 | EPI1165879 | A/H3 | Costa Rica | 2017-Nov-13 | A/Costa Rica/6790/2017 | Laboratorio Nacional de Influenza | Centers for Disease Control and Prevention |  | |
| EPI_ISL_296475 | EPI1163440 | A/H3 | Costa Rica | 2017-Nov-21 | A/Costa Rica/7285/2017 | Laboratorio Nacional de Influenza | Centers for Disease Control and Prevention |  | |
| EPI_ISL_296515 | EPI1163432 | A/H3 | Costa Rica | 2017-Nov-20 | A/Costa Rica/6964/2017 | Laboratorio Nacional de Influenza | Centers for Disease Control and Prevention |  | |
| EPI_ISL_296516 | EPI1166303 | A/H3 | Costa Rica | 2017-Nov-09 | A/Costa Rica/6370/2017 | Laboratorio Nacional de Influenza | Centers for Disease Control and Prevention |  | |
| EPI_ISL_296517 | EPI1086105 | A/H3 | Costa Rica | 2017-Aug-17 | A/Costa Rica/9473/2017 | Laboratorio Nacional de Influenza | Centers for Disease Control and Prevention |  | |
| EPI_ISL_296518 | EPI1086097 | A/H3 | Costa Rica | 2017-Aug-07 | A/Costa Rica/8476/2017 | Laboratorio Nacional de Influenza | Centers for Disease Control and Prevention |  | |
| EPI_ISL_296519 | EPI1086089 | A/H3 | Costa Rica | 2017-Jul-14 | A/Costa Rica/6684/2017 | Laboratorio Nacional de Influenza | Centers for Disease Control and Prevention |  | |
| EPI_ISL_296520 | EPI1086081 | A/H3 | Costa Rica | 2017-Jul-04 | A/Costa Rica/5406/2017 | Laboratorio Nacional de Influenza | Centers for Disease Control and Prevention |  | |
| EPI_ISL_296521 | EPI1086073 | A/H3 | Costa Rica | 2017-Jul-04 | A/Costa Rica/5326/2017 | Laboratorio Nacional de Influenza | Centers for Disease Control and Prevention |  | |
| EPI_ISL_296522 | EPI1086065 | A/H3 | Costa Rica | 2017-Jun-27 | A/Costa Rica/4869/2017 | Laboratorio Nacional de Influenza | Centers for Disease Control and Prevention |  | |
| EPI_ISL_296525 | EPI1086057 | A/H3 | Costa Rica | 2017-Jun-26 | A/Costa Rica/4667/2017 | Laboratorio Nacional de Influenza | Centers for Disease Control and Prevention |  | |
| EPI_ISL_282214 | EPI1082239 | B/Vic | Costa Rica | 2017-Jun-16 | B/Costa Rica/4040/2017 | Laboratorio Nacional de Influenza | Centers for Disease Control and Prevention |  | |
| EPI_ISL_282217 | EPI1082263 | B/Vic | Costa Rica | 2017-Aug-07 | B/Costa Rica/8330/2017 | Laboratorio Nacional de Influenza | Centers for Disease Control and Prevention |  | |
| EPI_ISL_282798 | EPI1085506 | B/Vic | Costa Rica | 2017-Aug-11 | B/Costa Rica/9213/2017 | Laboratorio Nacional de Influenza | Centers for Disease Control and Prevention |  | |
| EPI_ISL_296257 | EPI1164674 | B/Vic | Costa Rica | 2017-Nov-06 | B/Costa Rica/6551/2017 | Laboratorio Nacional de Influenza | Centers for Disease Control and Prevention |  | |
| EPI_ISL_296611 | EPI1166983 | B/Vic | Costa Rica | 2017-Dec-07 | B/Costa Rica/8338/2017 | Laboratorio Nacional de Influenza | Centers for Disease Control and Prevention |  | |
| EPI_ISL_282216 | EPI1082255 | B/Yam | Costa Rica | 2017-Aug-07 | B/Costa Rica/8474/2017 | Laboratorio Nacional de Influenza | Centers for Disease Control and Prevention |  | |
| EPI_ISL_282218 | EPI1082271 | B/Yam | Costa Rica | 2017-Aug-03 | B/Costa Rica/7952/2017 | Laboratorio Nacional de Influenza | Centers for Disease Control and Prevention |  | |
| EPI_ISL_282219 | EPI1082279 | B/Yam | Costa Rica | 2017-Aug-01 | B/Costa Rica/7727/2017 | Laboratorio Nacional de Influenza | Centers for Disease Control and Prevention |  | |
| EPI_ISL_283372 | EPI1089689 | B/Yam | Costa Rica | 2017-Aug-23 | B/Costa Rica/9789/2017 | Laboratorio Nacional de Influenza | Centers for Disease Control and Prevention |  | |
| EPI_ISL_296610 | EPI1166976 | B/Yam | Costa Rica | 2017-Nov-01 | B/Costa Rica/5694/2017 | Laboratorio Nacional de Influenza | Centers for Disease Control and Prevention |  | |
| EPI_ISL_299992 | EPI1268412 | A/H1pdm09 | Ecuador | 2018-Mar-19 | A/Ecuador/3174/2018 | INSPI | Centers for Disease Control and Prevention |  | |
| EPI_ISL_299993 | EPI1268404 | A/H1pdm09 | Ecuador | 2018-Mar-12 | A/Ecuador/3158/2018 | INSPI | Centers for Disease Control and Prevention |  | |
| EPI_ISL_299994 | EPI1268396 | A/H1pdm09 | Ecuador | 2018-Jul-03 | A/Ecuador/1168/2018 | INSPI | Centers for Disease Control and Prevention |  | |
| EPI_ISL_299995 | EPI1268388 | A/H1pdm09 | Ecuador | 2018-Mar-19 | A/Ecuador/7261/2018 | INSPI | Centers for Disease Control and Prevention |  | |
| EPI_ISL_299996 | EPI1268380 | A/H1pdm09 | Ecuador | 2018-Mar-06 | A/Ecuador/7215/2018 | INSPI | Centers for Disease Control and Prevention |  | |
| EPI_ISL_299997 | EPI1268372 | A/H1pdm09 | Ecuador | 2018-Mar-12 | A/Ecuador/7239/2018 | INSPI | Centers for Disease Control and Prevention |  | |
| EPI_ISL_300075 | EPI1268364 | A/H1pdm09 | Ecuador | 2018-Mar-13 | A/Ecuador/7240/2018 | INSPI | Centers for Disease Control and Prevention |  | |
| EPI_ISL_300076 | EPI1268356 | A/H1pdm09 | Ecuador | 2018-Feb-19 | A/Ecuador/653/2018 | INSPI | Centers for Disease Control and Prevention |  | |
| EPI_ISL_300077 | EPI1268349 | A/H1pdm09 | Ecuador | 2018-Feb-17 | A/Ecuador/1864/2018 | INSPI | Centers for Disease Control and Prevention |  | |
| EPI_ISL_300078 | EPI1268341 | A/H1pdm09 | Ecuador | 2018-Feb-07 | A/Ecuador/3904/2018 | INSPI | Centers for Disease Control and Prevention |  | |
| EPI_ISL_300079 | EPI1268333 | A/H1pdm09 | Ecuador | 2018-Feb-02 | A/Ecuador/2627/2018 | INSPI | Centers for Disease Control and Prevention |  | |
| EPI_ISL_300080 | EPI1268325 | A/H1pdm09 | Ecuador | 2018-Mar-01 | A/Ecuador/655/2018 | INSPI | Centers for Disease Control and Prevention |  | |
| EPI_ISL_300081 | EPI1268317 | A/H1pdm09 | Ecuador | 2018-Jun-02 | A/Ecuador/1155/2018 | INSPI | Centers for Disease Control and Prevention |  | |
| EPI_ISL_300082 | EPI1268309 | A/H1pdm09 | Ecuador | 2018-Apr-01 | A/Ecuador/1124/2018 | INSPI | Centers for Disease Control and Prevention |  | |
| EPI_ISL_300083 | EPI1252095 | A/H1pdm09 | Ecuador | 2017-Dec-12 | A/Ecuador/2953/2017 | INSPI | Centers for Disease Control and Prevention |  | |
| EPI_ISL_300084 | EPI1196402 | A/H1pdm09 | Ecuador | 2018-Jan-03 | A/Ecuador/262/2018 | INSPI | Centers for Disease Control and Prevention |  | |
| EPI_ISL_300085 | EPI1196386 | A/H1pdm09 | Ecuador | 2018-Jan-06 | A/Ecuador/2985/2018 | INSPI | Centers for Disease Control and Prevention |  | |
| EPI_ISL_300086 | EPI1196367 | A/H1pdm09 | Ecuador | 2018-Jan-10 | A/Ecuador/3748/2018 | INSPI | Centers for Disease Control and Prevention |  | |
| EPI_ISL_300087 | EPI1195421 | A/H1pdm09 | Ecuador | 2017-Dec-28 | A/Ecuador/997/2017 | INSPI | Centers for Disease Control and Prevention |  | |
| EPI_ISL_302838 | EPI1182054 | A/H1pdm09 | Ecuador | 2018-Jan-12 | A/Ecuador/14/2018 | INSPI | Centers for Disease Control and Prevention |  | |
| EPI_ISL_302974 | EPI1182046 | A/H1pdm09 | Ecuador | 2017-Dec-24 | A/Ecuador/614/2017 | INSPI | Centers for Disease Control and Prevention |  | |
| EPI_ISL_302977 | EPI1182039 | A/H1pdm09 | Ecuador | 2018-Jan-05 | A/Ecuador/72/2018 | INSPI | Centers for Disease Control and Prevention |  | |
| EPI_ISL_302980 | EPI1182031 | A/H1pdm09 | Ecuador | 2018-Jan-05 | A/Ecuador/263/2018 | INSPI | Centers for Disease Control and Prevention |  | |
| EPI_ISL_314049 | EPI1182023 | A/H1pdm09 | Ecuador | 2017-Nov-06 | A/Ecuador/533/2017 | INSPI | Centers for Disease Control and Prevention |  | |
| EPI_ISL_320241 | EPI1182015 | A/H1pdm09 | Ecuador | 2017-Dec-27 | A/Ecuador/3670/2017 | INSPI | Centers for Disease Control and Prevention |  | |
| EPI_ISL_320242 | EPI1182007 | A/H1pdm09 | Ecuador | 2018-Jan-02 | A/Ecuador/1755/2018 | INSPI | Centers for Disease Control and Prevention |  | |
| EPI_ISL_320243 | EPI1181999 | A/H1pdm09 | Ecuador | 2018-Jan-11 | A/Ecuador/385/2018 | INSPI | Centers for Disease Control and Prevention |  | |
| EPI_ISL_320244 | EPI1181991 | A/H1pdm09 | Ecuador | 2018-Jan-05 | A/Ecuador/739/2018 | INSPI | Centers for Disease Control and Prevention |  | |
| EPI_ISL_320245 | EPI1181984 | A/H1pdm09 | Ecuador | 2017-Dec-23 | A/Ecuador/625/2017 | INSPI | Centers for Disease Control and Prevention |  | |
| EPI_ISL_320246 | EPI1181976 | A/H1pdm09 | Ecuador | 2018-Jan-02 | A/Ecuador/016/2018 | INSPI | Centers for Disease Control and Prevention |  | |
| EPI_ISL_320247 | EPI1181969 | A/H1pdm09 | Ecuador | 2018-Jan-10 | A/Ecuador/270/2018 | INSPI | Centers for Disease Control and Prevention |  | |
| EPI_ISL_320248 | EPI1181961 | A/H1pdm09 | Ecuador | 2018-Jan-10 | A/Ecuador/271/2018 | INSPI | Centers for Disease Control and Prevention |  | |
| EPI_ISL_320249 | EPI1181365 | A/H1pdm09 | Ecuador | 2017-Dec-27 | A/Ecuador/731/2017 | INSPI | Centers for Disease Control and Prevention |  | |
| EPI_ISL_320250 | EPI1181357 | A/H1pdm09 | Ecuador | 2017-Dec-22 | A/Ecuador/609/2017 | INSPI | Centers for Disease Control and Prevention |  | |
| EPI_ISL_320251 | EPI1181349 | A/H1pdm09 | Ecuador | 2018-Jan-03 | A/Ecuador/032/2018 | INSPI | Centers for Disease Control and Prevention |  | |
| EPI_ISL_320252 | EPI1181341 | A/H1pdm09 | Ecuador | 2018-Jan-10 | A/Ecuador/272/2018 | INSPI | Centers for Disease Control and Prevention |  | |
| EPI_ISL_320253 | EPI1181333 | A/H1pdm09 | Ecuador | 2017-Dec-12 | A/Ecuador/2495/2017 | INSPI | Centers for Disease Control and Prevention |  | |
| EPI_ISL_320254 | EPI1181325 | A/H1pdm09 | Ecuador | 2018-Jan-19 | A/Ecuador/3798/2018 | INSPI | Centers for Disease Control and Prevention |  | |
| EPI_ISL_272886 | EPI1035215 | A/H3 | Ecuador | 2017-May-18 | A/Ecuador/1120/2017 | INSPI | Centers for Disease Control and Prevention |  | |
| EPI_ISL_299890 | EPI1180611 | A/H3 | Ecuador | 2017-Dec-17 | A/Ecuador/3868/2017 | INSPI | Centers for Disease Control and Prevention |  | |
| EPI_ISL_299991 | EPI1181317 | A/H3 | Ecuador | 2018-Jan-09 | A/Ecuador/103/2018 | INSPI | Centers for Disease Control and Prevention |  | |
| EPI_ISL_306258 | EPI1212715 | A/H3 | Ecuador | 2017-Dec-25 | A/Ecuador/1196/2017 | INSPI | Centers for Disease Control and Prevention |  | |
| EPI_ISL_320329 | EPI1268998 | A/H3 | Ecuador | 2018-Mar-20 | A/Ecuador/1898/2018 | INSPI | Centers for Disease Control and Prevention |  | |
| EPI_ISL_320330 | EPI1269006 | A/H3 | Ecuador | 2018-Apr-21 | A/Ecuador/1917/2018 | INSPI | Centers for Disease Control and Prevention |  | |
| EPI_ISL_320331 | EPI1269014 | A/H3 | Ecuador | 2018-May-26 | A/Ecuador/1938/2018 | INSPI | Centers for Disease Control and Prevention |  | |
| EPI_ISL_320332 | EPI1269022 | A/H3 | Ecuador | 2018-Mar-16 | A/Ecuador/4206/2018 | INSPI | Centers for Disease Control and Prevention |  | |
| EPI_ISL_300088 | EPI1182059 | B/Yam | Ecuador | 2017-Dec-19 | B/Ecuador/578/2017 | INSPI | Centers for Disease Control and Prevention |  | |
| EPI_ISL_300090 | EPI1182068 | B/Yam | Ecuador | 2017-Dec-27 | B/Ecuador/3673/2017 | INSPI | Centers for Disease Control and Prevention |  | |
| EPI_ISL_300091 | EPI1182076 | B/Yam | Ecuador | 2017-Dec-13 | B/Ecuador/1178/2017 | INSPI | Centers for Disease Control and Prevention |  | |
| EPI_ISL_320891 | EPI1272120 | B/Yam | Ecuador | 2018-Jul-03 | B/Ecuador/1418/2018 | INSPI | Centers for Disease Control and Prevention |  | |
| EPI_ISL_320922 | EPI1272360 | B/Yam | Ecuador | 2018-Jul-04 | B/Ecuador/1171/2018 | INSPI | Centers for Disease Control and Prevention |  | |
| EPI_ISL_320924 | EPI1272373 | B/Yam | Ecuador | 2018-Apr-29 | B/Ecuador/3953/2018 | INSPI | Centers for Disease Control and Prevention |  | |
| EPI_ISL_320925 | EPI1272381 | B/Yam | Ecuador | 2018-Jun-08 | B/Ecuador/3979/2018 | INSPI | Centers for Disease Control and Prevention |  | |
| EPI_ISL_320926 | EPI1272389 | B/Yam | Ecuador | 2018-Jun-28 | B/Ecuador/3997/2018 | INSPI | Centers for Disease Control and Prevention |  | |
| EPI_ISL_320927 | EPI1272397 | B/Yam | Ecuador | 2018-Jun-14 | B/Ecuador/1500/2018 | INSPI | Centers for Disease Control and Prevention |  | |
| EPI_ISL_320929 | EPI1272410 | B/Yam | Ecuador | 2018-Jun-29 | B/Ecuador/1218/2018 | INSPI | Centers for Disease Control and Prevention |  | |
| EPI_ISL_320930 | EPI1272418 | B/Yam | Ecuador | 2018-Jun-29 | B/Ecuador/0518/2018 | INSPI | Centers for Disease Control and Prevention |  | |
| EPI_ISL_320931 | EPI1272426 | B/Yam | Ecuador | 2018-Jun-18 | B/Ecuador/4278/2018 | INSPI | Centers for Disease Control and Prevention |  | |
| EPI_ISL_320934 | EPI1272447 | B/Yam | Ecuador | 2018-Jun-02 | B/Ecuador/2875/2018 | INSPI | Centers for Disease Control and Prevention |  | |
| EPI_ISL_320935 | EPI1272455 | B/Yam | Ecuador | 2018-Jul-06 | B/Ecuador/2018/2018 | INSPI | Centers for Disease Control and Prevention |  | |
| EPI_ISL_320936 | EPI1272463 | B/Yam | Ecuador | 2018-Mar-23 | B/Ecuador/1901/2018 | INSPI | Centers for Disease Control and Prevention |  | |
| EPI_ISL_312934 | EPI1260790 | A/H1pdm09 | Mexico | 2018-Jun-26 | A/Yucatan/InDRE1845/2018 | Instituto de diagnóstico y Referencia Epidemiologicos INDRE | Instituto de diagnóstico y Referencia Epidemiologicos (INDRE) |  | |
| EPI_ISL_316419 | EPI1260791 | A/H1pdm09 | Mexico | 2018-Jun-28 | A/Yucatan/InDRE1846/2018 | Instituto de diagnóstico y Referencia Epidemiologicos INDRE | Instituto de diagnóstico y Referencia Epidemiologicos (INDRE) |  | |
| EPI_ISL_316420 | EPI1282953 | A/H1pdm09 | Mexico | 2018-Jul-11 | A/Campeche/InDRE1975/2018 | Instituto de diagnóstico y Referencia Epidemiologicos INDRE | Instituto de diagnóstico y Referencia Epidemiologicos (INDRE) |  | |
| EPI_ISL_320190 | EPI1282954 | A/H1pdm09 | Mexico | 2018-Jul-09 | A/Campeche/InDRE1979/2018 | Instituto de diagnóstico y Referencia Epidemiologicos INDRE | Instituto de diagnóstico y Referencia Epidemiologicos (INDRE) |  | |
| EPI_ISL_320192 | EPI1245869 | A/H1pdm09 | Mexico | 2018-Jan-03 | A/Mexico/64/2018 | Laboratorio de Virus Respiratorio | Centers for Disease Control and Prevention |  | |
| EPI_ISL_320194 | EPI1267904 | A/H1pdm09 | Mexico | 2018-May-19 | A/Mexico/1703/2018 | Laboratorio de Virus Respiratorio | Centers for Disease Control and Prevention |  | |
| EPI_ISL_320195 | EPI1267920 | A/H1pdm09 | Mexico | 2018-May-16 | A/Mexico/1701/2018 | Laboratorio de Virus Respiratorio | Centers for Disease Control and Prevention |  | |
| EPI_ISL_320196 | EPI1267936 | A/H1pdm09 | Mexico | 2018-May-18 | A/Mexico/1670/2018 | Laboratorio de Virus Respiratorio | Centers for Disease Control and Prevention |  | |
| EPI_ISL_320197 | EPI1267944 | A/H1pdm09 | Mexico | 2018-May-17 | A/Mexico/1669/2018 | Laboratorio de Virus Respiratorio | Centers for Disease Control and Prevention |  | |
| EPI_ISL_320198 | EPI1267952 | A/H1pdm09 | Mexico | 2018-May-22 | A/Mexico/1657/2018 | Laboratorio de Virus Respiratorio | Centers for Disease Control and Prevention |  | |
| EPI_ISL_320199 | EPI1267960 | A/H1pdm09 | Mexico | 2018-May-04 | A/Mexico/1633/2018 | Laboratorio de Virus Respiratorio | Centers for Disease Control and Prevention |  | |
| EPI_ISL_320201 | EPI1267968 | A/H1pdm09 | Mexico | 2018-May-28 | A/Mexico/1718/2018 | Laboratorio de Virus Respiratorio | Centers for Disease Control and Prevention |  | |
| EPI_ISL_320202 | EPI1267976 | A/H1pdm09 | Mexico | 2018-May-25 | A/Mexico/1717/2018 | Laboratorio de Virus Respiratorio | Centers for Disease Control and Prevention |  | |
| EPI_ISL_320203 | EPI1267991 | A/H1pdm09 | Mexico | 2018-May-30 | A/Mexico/1722/2018 | Laboratorio de Virus Respiratorio | Centers for Disease Control and Prevention |  | |
| EPI_ISL_322981 | EPI1267999 | A/H1pdm09 | Mexico | 2018-Jun-07 | A/Mexico/1747/2018 | Laboratorio de Virus Respiratorio | Centers for Disease Control and Prevention |  | |
| EPI_ISL_322982 | EPI1268007 | A/H1pdm09 | Mexico | 2018-May-22 | A/Mexico/1746/2018 | Laboratorio de Virus Respiratorio | Centers for Disease Control and Prevention |  | |
| EPI_ISL_276651 | EPI1053512 | A/H3 | Mexico | 2017-Jul-25 | A/Chiapas/InDRE2528/2017 | Instituto de diagnóstico y Referencia Epidemiologicos INDRE | Instituto de diagnóstico y Referencia Epidemiologicos (INDRE) |  | |
| EPI_ISL_291624 | EPI1301448 | A/H3 | Mexico | 2017-Nov-17 | A/Mexico/SAR30012N/2017 |  | Import from public-domain | Sitz,C.R.; Koster,B.L.; Balansay-Ames,M.S.; Graf,P.C.; Myers,C.A. | |
| EPI_ISL_291625 | EPI1301540 | A/H3 | Mexico | 2017-Nov-13 | A/Mexico/SAR30007N/2017 |  | Import from public-domain | Sitz,C.R.; Koster,B.L.; Balansay-Ames,M.S.; Graf,P.C.; Myers,C.A. | |
| EPI_ISL_291626 | EPI1268639 | A/H3 | Mexico | 2018-May-09 | A/Mexico/1641/2018 | Laboratorio de Virus Respiratorio | Centers for Disease Control and Prevention |  | |
| EPI_ISL_291627 | EPI1268631 | A/H3 | Mexico | 2018-May-07 | A/Mexico/1651/2018 | Laboratorio de Virus Respiratorio | Centers for Disease Control and Prevention |  | |
| EPI_ISL_291628 | EPI1268624 | A/H3 | Mexico | 2018-May-24 | A/Mexico/1707/2018 | Laboratorio de Virus Respiratorio | Centers for Disease Control and Prevention |  | |
| EPI_ISL_291629 | EPI1141867 | A/H3 | Mexico | 2017-Aug-30 | A/Mexico/2638/2017 | Laboratorio de Virus Respiratorio | Centers for Disease Control and Prevention |  | |
| EPI_ISL_291630 | EPI1141859 | A/H3 | Mexico | 2017-Aug-22 | A/Mexico/2630/2017 | Laboratorio de Virus Respiratorio | Centers for Disease Control and Prevention |  | |
| EPI_ISL_291631 | EPI1141851 | A/H3 | Mexico | 2017-Aug-16 | A/Mexico/2624/2017 | Laboratorio de Virus Respiratorio | Centers for Disease Control and Prevention |  | |
| EPI_ISL_291632 | EPI1141843 | A/H3 | Mexico | 2017-Aug-14 | A/Mexico/2623/2017 | Laboratorio de Virus Respiratorio | Centers for Disease Control and Prevention |  | |
| EPI_ISL_291633 | EPI1141836 | A/H3 | Mexico | 2017-Aug-14 | A/Mexico/2611/2017 | Laboratorio de Virus Respiratorio | Centers for Disease Control and Prevention |  | |
| EPI_ISL_291634 | EPI1141828 | A/H3 | Mexico | 2017-Aug-10 | A/Mexico/2610/2017 | Laboratorio de Virus Respiratorio | Centers for Disease Control and Prevention |  | |
| EPI_ISL_291635 | EPI1141820 | A/H3 | Mexico | 2017-Jul-31 | A/Mexico/2577/2017 | Laboratorio de Virus Respiratorio | Centers for Disease Control and Prevention |  | |
| EPI_ISL_291636 | EPI1141813 | A/H3 | Mexico | 2017-Aug-02 | A/Mexico/2570/2017 | Laboratorio de Virus Respiratorio | Centers for Disease Control and Prevention |  | |
| EPI_ISL_291637 | EPI1141805 | A/H3 | Mexico | 2017-Aug-03 | A/Mexico/2567/2017 | Laboratorio de Virus Respiratorio | Centers for Disease Control and Prevention |  | |
| EPI_ISL_291638 | EPI1141797 | A/H3 | Mexico | 2017-Jul-28 | A/Mexico/2557/2017 | Laboratorio de Virus Respiratorio | Centers for Disease Control and Prevention |  | |
| EPI_ISL_291639 | EPI1141789 | A/H3 | Mexico | 2017-Jul-26 | A/Mexico/2553/2017 | Laboratorio de Virus Respiratorio | Centers for Disease Control and Prevention |  | |
| EPI_ISL_320282 | EPI1141782 | A/H3 | Mexico | 2017-Jul-10 | A/Mexico/2523/2017 | Laboratorio de Virus Respiratorio | Centers for Disease Control and Prevention |  | |
| EPI_ISL_320283 | EPI1141774 | A/H3 | Mexico | 2017-Jul-08 | A/Mexico/2514/2017 | Laboratorio de Virus Respiratorio | Centers for Disease Control and Prevention |  | |
| EPI_ISL_320284 | EPI1141766 | A/H3 | Mexico | 2017-Jun-29 | A/Mexico/2496/2017 | Laboratorio de Virus Respiratorio | Centers for Disease Control and Prevention |  | |
| EPI_ISL_327645 | EPI1141758 | A/H3 | Mexico | 2017-Jul-06 | A/Mexico/2495/2017 | Laboratorio de Virus Respiratorio | Centers for Disease Control and Prevention |  | |
| EPI_ISL_327646 | EPI1141750 | A/H3 | Mexico | 2017-Jul-05 | A/Mexico/2483/2017 | Laboratorio de Virus Respiratorio | Centers for Disease Control and Prevention |  | |
| EPI_ISL_320261 | EPI1268465 | B/Vic | Mexico | 2018-May-14 | B/Mexico/1710/2018 | Laboratorio de Virus Respiratorio | Centers for Disease Control and Prevention |  | |
| EPI_ISL_320262 | EPI1268473 | B/Vic | Mexico | 2018-May-30 | B/Mexico/1721/2018 | Laboratorio de Virus Respiratorio | Centers for Disease Control and Prevention |  | |
| EPI_ISL_320263 | EPI1268481 | B/Vic | Mexico | 2018-May-23 | B/Mexico/1719/2018 | Laboratorio de Virus Respiratorio | Centers for Disease Control and Prevention |  | |
| EPI_ISL_320890 | EPI1272112 | B/Yam | Mexico | 2018-May-07 | B/Mexico/1650/2018 | Laboratorio de Virus Respiratorio | Centers for Disease Control and Prevention |  | |
| EPI_ISL_320892 | EPI1272128 | B/Yam | Mexico | 2018-Jun-08 | B/Mexico/1739/2018 | Laboratorio de Virus Respiratorio | Centers for Disease Control and Prevention |  | |
| EPI_ISL_320894 | EPI1272144 | B/Yam | Mexico | 2018-May-28 | B/Mexico/1736/2018 | Laboratorio de Virus Respiratorio | Centers for Disease Control and Prevention |  | |
| EPI_ISL_320928 | EPI1272405 | B/Yam | Mexico | 2018-Jun-04 | B/Mexico/1711/2018 | Laboratorio de Virus Respiratorio | Centers for Disease Control and Prevention |  | |
| EPI_ISL_321953 | EPI1277886 | A/H1pdm09 | Paraguay | 2018-Jul-12 | A/Paraguay/0530/2018 | Central Laboratory of Public Health | Centers for Disease Control and Prevention |  | |
| EPI_ISL_321954 | EPI1277894 | A/H1pdm09 | Paraguay | 2018-Jul-15 | A/Paraguay/2453/2018 | Central Laboratory of Public Health | Centers for Disease Control and Prevention |  | |
| EPI_ISL_321955 | EPI1277902 | A/H1pdm09 | Paraguay | 2018-Jul-09 | A/Paraguay/9540/2018 | Central Laboratory of Public Health | Centers for Disease Control and Prevention |  | |
| EPI_ISL_321964 | EPI1277969 | A/H1pdm09 | Paraguay | 2018-Apr-13 | A/Paraguay/9264/2018 | Central Laboratory of Public Health | Centers for Disease Control and Prevention |  | |
| EPI_ISL_321966 | EPI1277982 | A/H1pdm09 | Paraguay | 2018-Jun-22 | A/Paraguay/7692/2018 | Central Laboratory of Public Health | Centers for Disease Control and Prevention |  | |
| EPI_ISL_321970 | EPI1278012 | A/H1pdm09 | Paraguay | 2018-Jun-06 | A/Paraguay/3715/2018 | Central Laboratory of Public Health | Centers for Disease Control and Prevention |  | |
| EPI_ISL_321971 | EPI1278020 | A/H1pdm09 | Paraguay | 2018-Jun-04 | A/Paraguay/3711/2018 | Central Laboratory of Public Health | Centers for Disease Control and Prevention |  | |
| EPI_ISL_321976 | EPI1278060 | A/H1pdm09 | Paraguay | 2018-May-22 | A/Paraguay/0622/2018 | Central Laboratory of Public Health | Centers for Disease Control and Prevention |  | |
| EPI_ISL_331099 | EPI1318107 | A/H1pdm09 | Paraguay | 2018-May-22 | A/Paraguay/280622/2018 | Laboratorio Central de Salud Publica | Laboratorio Central de Salud Publica | Cynthia,Vazquez; Shirley,Villalba; Maria Jose,Ortega; Andrea,Gomez de la Fuente; Juan,Torales | |
| EPI_ISL_331102 | EPI1318110 | A/H1pdm09 | Paraguay | 2018-Jun-04 | A/Paraguay/283711/2018 | Laboratorio Central de Salud Publica | Laboratorio Central de Salud Publica | Cynthia,Vazquez; Shirley,Villalba; Maria Jose,Ortega; Andrea,Gomez de la Fuente; Juan,Torales | |
| EPI_ISL_281550 | EPI1318116 | A/H3 | Paraguay | 2018-Jul-19 | A/Paraguay/291838/2018 | Laboratorio Central de Salud Publica | Laboratorio Central de Salud Publica | Cynthia,Vazquez; Shirley,Villalba; Maria Jose,Ortega; Andrea,Gomez de la Fuente; Juan,Torales | |
| EPI_ISL_281551 | EPI1318115 | A/H3 | Paraguay | 2018-Jul-09 | A/Paraguay/290469/2018 | Laboratorio Central de Salud Publica | Laboratorio Central de Salud Publica | Cynthia,Vazquez; Shirley,Villalba; Maria Jose,Ortega; Andrea,Gomez de la Fuente; Juan,Torales | |
| EPI_ISL_281552 | EPI1318113 | A/H3 | Paraguay | 2018-Jun-15 | A/Paraguay/285719/2018 | Laboratorio Central de Salud Publica | Laboratorio Central de Salud Publica | Cynthia,Vazquez; Shirley,Villalba; Maria Jose,Ortega; Andrea,Gomez de la Fuente; Juan,Torales | |
| EPI_ISL_281727 | EPI1318112 | A/H3 | Paraguay | 2018-Jun-18 | A/Paraguay/285647/2018 | Laboratorio Central de Salud Publica | Laboratorio Central de Salud Publica | Cynthia,Vazquez; Shirley,Villalba; Maria Jose,Ortega; Andrea,Gomez de la Fuente; Juan,Torales | |
| EPI_ISL_321864 | EPI1318109 | A/H3 | Paraguay | 2018-May-25 | A/Paraguay/281722/2018 | Laboratorio Central de Salud Publica | Laboratorio Central de Salud Publica | Cynthia,Vazquez; Shirley,Villalba; Maria Jose,Ortega; Andrea,Gomez de la Fuente; Juan,Torales | |
| EPI_ISL_321865 | EPI1318105 | A/H3 | Paraguay | 2018-May-21 | A/Paraguay/280011/2018 | Laboratorio Central de Salud Publica | Laboratorio Central de Salud Publica | Cynthia,Vazquez; Shirley,Villalba; Maria Jose,Ortega; Andrea,Gomez de la Fuente; Juan,Torales | |
| EPI_ISL_321866 | EPI1318102 | A/H3 | Paraguay | 2018-May-18 | A/Paraguay/279130/2018 | Laboratorio Central de Salud Publica | Laboratorio Central de Salud Publica | Cynthia,Vazquez; Shirley,Villalba; Maria Jose,Ortega; Andrea,Gomez de la Fuente; Juan,Torales | |
| EPI_ISL_321867 | EPI1318101 | A/H3 | Paraguay | 2018-May-05 | A/Paraguay/278607/2018 | Laboratorio Central de Salud Publica | Laboratorio Central de Salud Publica | Cynthia,Vazquez; Shirley,Villalba; Maria Jose,Ortega; Andrea,Gomez de la Fuente; Juan,Torales | |
| EPI_ISL_321868 | EPI1277306 | A/H3 | Paraguay | 2018-May-18 | A/Paraguay/9130/2018 | Central Laboratory of Public Health | Centers for Disease Control and Prevention |  | |
| EPI_ISL_321870 | EPI1277298 | A/H3 | Paraguay | 2018-Jun-07 | A/Paraguay/3983/2018 | Central Laboratory of Public Health | Centers for Disease Control and Prevention |  | |
| EPI_ISL_321872 | EPI1277290 | A/H3 | Paraguay | 2018-Jun-28 | A/Paraguay/7726/2018 | Central Laboratory of Public Health | Centers for Disease Control and Prevention |  | |
| EPI_ISL_321873 | EPI1277282 | A/H3 | Paraguay | 2018-May-28 | A/Paraguay/1723/2018 | Central Laboratory of Public Health | Centers for Disease Control and Prevention |  | |
| EPI_ISL_321874 | EPI1277274 | A/H3 | Paraguay | 2018-May-25 | A/Paraguay/1722/2018 | Central Laboratory of Public Health | Centers for Disease Control and Prevention |  | |
| EPI_ISL_321875 | EPI1277266 | A/H3 | Paraguay | 2018-Jun-01 | A/Paraguay/3985/2018 | Central Laboratory of Public Health | Centers for Disease Control and Prevention |  | |
| EPI_ISL_321876 | EPI1277258 | A/H3 | Paraguay | 2018-May-21 | A/Paraguay/0011/2018 | Central Laboratory of Public Health | Centers for Disease Control and Prevention |  | |
| EPI_ISL_321877 | EPI1277250 | A/H3 | Paraguay | 2018-Mar-02 | A/Paraguay/4199/2018 | Central Laboratory of Public Health | Centers for Disease Control and Prevention |  | |
| EPI_ISL_321878 | EPI1277242 | A/H3 | Paraguay | 2018-May-18 | A/Paraguay/8607/2018 | Central Laboratory of Public Health | Centers for Disease Control and Prevention |  | |
| EPI_ISL_321879 | EPI1277226 | A/H3 | Paraguay | 2018-Mar-12 | A/Paraguay/9159/2018 | Central Laboratory of Public Health | Centers for Disease Control and Prevention |  | |
| EPI_ISL_321880 | EPI1277210 | A/H3 | Paraguay | 2018-Jul-19 | A/Paraguay/1838/2018 | Central Laboratory of Public Health | Centers for Disease Control and Prevention |  | |
| EPI_ISL_331094 | EPI1277202 | A/H3 | Paraguay | 2018-Jul-15 | A/Paraguay/1074/2018 | Central Laboratory of Public Health | Centers for Disease Control and Prevention |  | |
| EPI_ISL_331095 | EPI1277194 | A/H3 | Paraguay | 2018-Jul-19 | A/Paraguay/1839/2018 | Central Laboratory of Public Health | Centers for Disease Control and Prevention |  | |
| EPI_ISL_331097 | EPI1277186 | A/H3 | Paraguay | 2018-Jul-16 | A/Paraguay/1296/2018 | Central Laboratory of Public Health | Centers for Disease Control and Prevention |  | |
| EPI_ISL_331101 | EPI1277178 | A/H3 | Paraguay | 2018-Jul-24 | A/Paraguay/3019/2018 | Central Laboratory of Public Health | Centers for Disease Control and Prevention |  | |
| EPI_ISL_331104 | EPI1079436 | A/H3 | Paraguay | 2017-May-10 | A/Paraguay/8340/2017 | Central Laboratory of Public Health | Centers for Disease Control and Prevention |  | |
| EPI_ISL_331105 | EPI1078030 | A/H3 | Paraguay | 2017-May-19 | A/Paraguay/9992/2017 | Central Laboratory of Public Health | Centers for Disease Control and Prevention |  | |
| EPI_ISL_331107 | EPI1078022 | A/H3 | Paraguay | 2017-May-15 | A/Paraguay/8969/2017 | Central Laboratory of Public Health | Centers for Disease Control and Prevention |  | |
| EPI_ISL_331108 | EPI1078014 | A/H3 | Paraguay | 2017-May-12 | A/Paraguay/8873/2017 | Central Laboratory of Public Health | Centers for Disease Control and Prevention |  | |
| EPI_ISL_322001 | EPI1278255 | B/Vic | Paraguay | 2018-Jun-29 | B/Paraguay/8729/2018 | Central Laboratory of Public Health | Centers for Disease Control and Prevention |  | |
| EPI_ISL_322002 | EPI1278263 | B/Vic | Paraguay | 2018-Jul-02 | B/Paraguay/8730/2018 | Central Laboratory of Public Health | Centers for Disease Control and Prevention |  | |
| EPI_ISL_322011 | EPI1278335 | B/Vic | Paraguay | 2018-Jun-08 | B/Paraguay/5924/2018 | Central Laboratory of Public Health | Centers for Disease Control and Prevention |  | |
| EPI_ISL_322013 | EPI1278351 | B/Vic | Paraguay | 2018-May-23 | B/Paraguay/0620/2018 | Central Laboratory of Public Health | Centers for Disease Control and Prevention |  | |
| EPI_ISL_322017 | EPI1278383 | B/Vic | Paraguay | 2018-Mar-02 | B/Paraguay/4209/2018 | Central Laboratory of Public Health | Centers for Disease Control and Prevention |  | |
| EPI_ISL_322020 | EPI1278407 | B/Vic | Paraguay | 2018-May-09 | B/Paraguay/7301/2018 | Central Laboratory of Public Health | Centers for Disease Control and Prevention |  | |
| EPI_ISL_331093 | EPI1318100 | B/Vic | Paraguay | 2018-May-09 | B/Paraguay/277301/2018 | Laboratorio Central de Salud Publica | Laboratorio Central de Salud Publica | Cynthia,Vazquez; Shirley,Villalba; Maria Jose,Ortega; Andrea,Gomez de la Fuente; Juan,Torales | |
| EPI_ISL_331098 | EPI1318106 | B/Vic | Paraguay | 2018-May-23 | B/Paraguay/280620/2018 | Laboratorio Central de Salud Publica | Laboratorio Central de Salud Publica | Cynthia,Vazquez; Shirley,Villalba; Maria Jose,Ortega; Andrea,Gomez de la Fuente; Juan,Torales | |
| EPI_ISL_331100 | EPI1318108 | B/Vic | Paraguay | 2018-May-28 | B/Paraguay/281400/2018 | Laboratorio Central de Salud Publica | Laboratorio Central de Salud Publica | Cynthia,Vazquez; Shirley,Villalba; Maria Jose,Ortega; Andrea,Gomez de la Fuente; Juan,Torales | |
| EPI_ISL_331106 | EPI1318114 | B/Vic | Paraguay | 2018-Jun-08 | B/Paraguay/285924/2018 | Laboratorio Central de Salud Publica | Laboratorio Central de Salud Publica | Cynthia,Vazquez; Shirley,Villalba; Maria Jose,Ortega; Andrea,Gomez de la Fuente; Juan,Torales | |
| EPI_ISL_281681 | EPI1318098 | B/Yam | Paraguay | 2018-Mar-28 | B/Paraguay/263659/2018 | Central Laboratory of Public Health | Laboratorio Central de Salud Publica | Cynthia,Vazquez; Shirley,Villalba; Maria Jose,Ortega; Andrea,Gomez de la Fuente; Juan,Torales | |
| EPI_ISL_282048 | EPI1318099 | B/Yam | Paraguay | 2018-Apr-17 | B/Paraguay/270336/2018 | Laboratorio Central de Salud Publica | Laboratorio Central de Salud Publica | Cynthia,Vazquez; Shirley,Villalba; Maria Jose,Ortega; Andrea,Gomez de la Fuente; Juan,Torales | |
| EPI_ISL_321997 | EPI1318111 | B/Yam | Paraguay | 2018-Jun-10 | B/Paraguay/283761/2018 | Laboratorio Central de Salud Publica | Laboratorio Central de Salud Publica | Cynthia,Vazquez; Shirley,Villalba; Maria Jose,Ortega; Andrea,Gomez de la Fuente; Juan,Torales | |
| EPI_ISL_322014 | EPI1318117 | B/Yam | Paraguay | 2018-Jul-17 | B/Paraguay/291605/2018 | Laboratorio Central de Salud Publica | Laboratorio Central de Salud Publica | Cynthia,Vazquez; Shirley,Villalba; Maria Jose,Ortega; Andrea,Gomez de la Fuente; Juan,Torales | |
| EPI_ISL_322016 | EPI1079018 | B/Yam | Paraguay | 2017-May-09 | B/Paraguay/8072/2017 | Central Laboratory of Public Health | Centers for Disease Control and Prevention |  | |
| EPI_ISL_322021 | EPI1081609 | B/Yam | Paraguay | 2017-May-09 | B/Paraguay/8065/2017 | Central Laboratory of Public Health | Centers for Disease Control and Prevention |  | |
| EPI_ISL_322022 | EPI1278223 | B/Yam | Paraguay | 2018-Jul-29 | B/Paraguay/3318/2018 | Central Laboratory of Public Health | Centers for Disease Control and Prevention |  | |
| EPI_ISL_322024 | EPI1278359 | B/Yam | Paraguay | 2018-Apr-17 | B/Paraguay/0336/2018 | Central Laboratory of Public Health | Centers for Disease Control and Prevention |  | |
| EPI_ISL_329917 | EPI1278375 | B/Yam | Paraguay | 2018-Jun-13 | B/Paraguay/4596/2018 | Central Laboratory of Public Health | Centers for Disease Control and Prevention |  | |
| EPI_ISL_331091 | EPI1278415 | B/Yam | Paraguay | 2018-Jun-18 | B/Paraguay/6288/2018 | Central Laboratory of Public Health | Centers for Disease Control and Prevention |  | |
| EPI_ISL_331092 | EPI1278423 | B/Yam | Paraguay | 2018-Jul-30 | B/Paraguay/3517/2018 | Central Laboratory of Public Health | Centers for Disease Control and Prevention |  | |
| EPI_ISL_331103 | EPI1278439 | B/Yam | Paraguay | 2018-Mar-19 | B/Paraguay/0343/2018 | Central Laboratory of Public Health | Centers for Disease Control and Prevention |  | |
| EPI_ISL_331109 | EPI1311134 | B/Yam | Paraguay | 2018-Apr-24 | B/Paraguay/2784/2018 | Central Laboratory of Public Health | Centers for Disease Control and Prevention |  | |
| EPI_ISL_319804 | EPI1278052 | A/H1pdm09 | Peru | 2018-Jun-06 | A/Peru/59818/2018 | Laboratorio de Referencia Nacional Virus Respiratorios, Instituto Nacional de Salud | Centers for Disease Control and Prevention |  | |
| EPI_ISL_319805 | EPI1278028 | A/H1pdm09 | Peru | 2018-Jun-11 | A/Peru/45918/2018 | Laboratorio de Referencia Nacional Virus Respiratorios, Instituto Nacional de Salud | Centers for Disease Control and Prevention |  | |
| EPI_ISL_319806 | EPI1277954 | A/H1pdm09 | Peru | 2018-Jun-09 | A/Peru/67118/2018 | Laboratorio de Referencia Nacional Virus Respiratorios, Instituto Nacional de Salud | Centers for Disease Control and Prevention |  | |
| EPI_ISL_319807 | EPI1277931 | A/H1pdm09 | Peru | 2018-Jun-04 | A/Peru/96218/2018 | Laboratorio de Referencia Nacional Virus Respiratorios, Instituto Nacional de Salud | Centers for Disease Control and Prevention |  | |
| EPI_ISL_319808 | EPI1277785 | A/H1pdm09 | Peru | 2018-Jun-07 | A/Peru/65918/2018 | Laboratorio de Referencia Nacional Virus Respiratorios, Instituto Nacional de Salud | Centers for Disease Control and Prevention |  | |
| EPI_ISL_319809 | EPI1277762 | A/H1pdm09 | Peru | 2018-Jun-06 | A/Peru/96418/2018 | Laboratorio de Referencia Nacional Virus Respiratorios, Instituto Nacional de Salud | Centers for Disease Control and Prevention |  | |
| EPI_ISL_319810 | EPI1267133 | A/H1pdm09 | Peru | 2018-Jun-11 | A/Peru/2718/2018 | Laboratorio de Referencia Nacional Virus Respiratorios, Instituto Nacional de Salud | Centers for Disease Control and Prevention |  | |
| EPI_ISL_319811 | EPI1267125 | A/H1pdm09 | Peru | 2018-Jun-08 | A/Peru/2318/2018 | Laboratorio de Referencia Nacional Virus Respiratorios, Instituto Nacional de Salud | Centers for Disease Control and Prevention |  | |
| EPI_ISL_319812 | EPI1267117 | A/H1pdm09 | Peru | 2018-Jun-07 | A/Peru/9318/2018 | Laboratorio de Referencia Nacional Virus Respiratorios, Instituto Nacional de Salud | Centers for Disease Control and Prevention |  | |
| EPI_ISL_319813 | EPI1267109 | A/H1pdm09 | Peru | 2018-Jun-08 | A/Peru/7718/2018 | Laboratorio de Referencia Nacional Virus Respiratorios, Instituto Nacional de Salud | Centers for Disease Control and Prevention |  | |
| EPI_ISL_319814 | EPI1267101 | A/H1pdm09 | Peru | 2018-Jun-06 | A/Peru/5118/2018 | Laboratorio de Referencia Nacional Virus Respiratorios, Instituto Nacional de Salud | Centers for Disease Control and Prevention |  | |
| EPI_ISL_319815 | EPI1267096 | A/H1pdm09 | Peru | 2018-Jun-05 | A/Peru/4918/2018 | Laboratorio de Referencia Nacional Virus Respiratorios, Instituto Nacional de Salud | Centers for Disease Control and Prevention |  | |
| EPI_ISL_319816 | EPI1267088 | A/H1pdm09 | Peru | 2018-Jun-06 | A/Peru/3718/2018 | Laboratorio de Referencia Nacional Virus Respiratorios, Instituto Nacional de Salud | Centers for Disease Control and Prevention |  | |
| EPI_ISL_319817 | EPI1267080 | A/H1pdm09 | Peru | 2018-Jun-07 | A/Peru/2618/2018 | Laboratorio de Referencia Nacional Virus Respiratorios, Instituto Nacional de Salud | Centers for Disease Control and Prevention |  | |
| EPI_ISL_319818 | EPI1267072 | A/H1pdm09 | Peru | 2018-Jun-01 | A/Peru/6518/2018 | Laboratorio de Referencia Nacional Virus Respiratorios, Instituto Nacional de Salud | Centers for Disease Control and Prevention |  | |
| EPI_ISL_319819 | EPI1267064 | A/H1pdm09 | Peru | 2018-Jun-04 | A/Peru/6418/2018 | Laboratorio de Referencia Nacional Virus Respiratorios, Instituto Nacional de Salud | Centers for Disease Control and Prevention |  | |
| EPI_ISL_319820 | EPI1267056 | A/H1pdm09 | Peru | 2018-May-31 | A/Peru/6218/2018 | Laboratorio de Referencia Nacional Virus Respiratorios, Instituto Nacional de Salud | Centers for Disease Control and Prevention |  | |
| EPI_ISL_319821 | EPI1267048 | A/H1pdm09 | Peru | 2018-May-31 | A/Peru/5218/2018 | Laboratorio de Referencia Nacional Virus Respiratorios, Instituto Nacional de Salud | Centers for Disease Control and Prevention |  | |
| EPI_ISL_319822 | EPI1267040 | A/H1pdm09 | Peru | 2018-Jun-06 | A/Peru/9918/2018 | Laboratorio de Referencia Nacional Virus Respiratorios, Instituto Nacional de Salud | Centers for Disease Control and Prevention |  | |
| EPI_ISL_319823 | EPI1267034 | A/H1pdm09 | Peru | 2018-Jun-05 | A/Peru/5618/2018 | Laboratorio de Referencia Nacional Virus Respiratorios, Instituto Nacional de Salud | Centers for Disease Control and Prevention |  | |
| EPI_ISL_319824 | EPI1267026 | A/H1pdm09 | Peru | 2018-May-24 | A/Peru/7218/2018 | Laboratorio de Referencia Nacional Virus Respiratorios, Instituto Nacional de Salud | Centers for Disease Control and Prevention |  | |
| EPI_ISL_319825 | EPI1267018 | A/H1pdm09 | Peru | 2018-May-24 | A/Peru/6718/2018 | Laboratorio de Referencia Nacional Virus Respiratorios, Instituto Nacional de Salud | Centers for Disease Control and Prevention |  | |
| EPI_ISL_319826 | EPI1267010 | A/H1pdm09 | Peru | 2018-May-28 | A/Peru/6318/2018 | Laboratorio de Referencia Nacional Virus Respiratorios, Instituto Nacional de Salud | Centers for Disease Control and Prevention |  | |
| EPI_ISL_319827 | EPI1267002 | A/H1pdm09 | Peru | 2018-May-22 | A/Peru/9818/2018 | Laboratorio de Referencia Nacional Virus Respiratorios, Instituto Nacional de Salud | Centers for Disease Control and Prevention |  | |
| EPI_ISL_321937 | EPI1266994 | A/H1pdm09 | Peru | 2018-Apr-13 | A/Peru/2918/2018 | Laboratorio de Referencia Nacional Virus Respiratorios, Instituto Nacional de Salud | Centers for Disease Control and Prevention |  | |
| EPI_ISL_321940 | EPI1266986 | A/H1pdm09 | Peru | 2018-Jun-08 | A/Peru/9118/2018 | Laboratorio de Referencia Nacional Virus Respiratorios, Instituto Nacional de Salud | Centers for Disease Control and Prevention |  | |
| EPI_ISL_321959 | EPI1266978 | A/H1pdm09 | Peru | 2018-Feb-20 | A/Peru/7418/2018 | Laboratorio de Referencia Nacional Virus Respiratorios, Instituto Nacional de Salud | Centers for Disease Control and Prevention |  | |
| EPI_ISL_321962 | EPI1266970 | A/H1pdm09 | Peru | 2018-Mar-05 | A/Peru/7118/2018 | Laboratorio de Referencia Nacional Virus Respiratorios, Instituto Nacional de Salud | Centers for Disease Control and Prevention |  | |
| EPI_ISL_321972 | EPI1266962 | A/H1pdm09 | Peru | 2018-Jun-27 | A/Peru/0618/2018 | Laboratorio de Referencia Nacional Virus Respiratorios, Instituto Nacional de Salud | Centers for Disease Control and Prevention |  | |
| EPI_ISL_321975 | EPI1266954 | A/H1pdm09 | Peru | 2018-Jun-06 | A/Peru/4018/2018 | Laboratorio de Referencia Nacional Virus Respiratorios, Instituto Nacional de Salud | Centers for Disease Control and Prevention |  | |
| EPI_ISL_273677 | EPI1266357 | A/H3 | Peru | 2018-May-30 | A/Peru/3018/2018 | Laboratorio de Referencia Nacional Virus Respiratorios, Instituto Nacional de Salud | Centers for Disease Control and Prevention |  | |
| EPI_ISL_273679 | EPI1266349 | A/H3 | Peru | 2018-May-25 | A/Peru/5918/2018 | Laboratorio de Referencia Nacional Virus Respiratorios, Instituto Nacional de Salud | Centers for Disease Control and Prevention |  | |
| EPI_ISL_273681 | EPI1266341 | A/H3 | Peru | 2018-Apr-10 | A/Peru/0718/2018 | Laboratorio de Referencia Nacional Virus Respiratorios, Instituto Nacional de Salud | Centers for Disease Control and Prevention |  | |
| EPI_ISL_273683 | EPI1266333 | A/H3 | Peru | 2018-Apr-10 | A/Peru/0918/2018 | Laboratorio de Referencia Nacional Virus Respiratorios, Instituto Nacional de Salud | Centers for Disease Control and Prevention |  | |
| EPI_ISL_273685 | EPI1266325 | A/H3 | Peru | 2018-Apr-09 | A/Peru/2118/2018 | Laboratorio de Referencia Nacional Virus Respiratorios, Instituto Nacional de Salud | Centers for Disease Control and Prevention |  | |
| EPI_ISL_273687 | EPI1266317 | A/H3 | Peru | 2018-Apr-09 | A/Peru/1318/2018 | Laboratorio de Referencia Nacional Virus Respiratorios, Instituto Nacional de Salud | Centers for Disease Control and Prevention |  | |
| EPI_ISL_273688 | EPI1266309 | A/H3 | Peru | 2018-Apr-03 | A/Peru/3318/2018 | Laboratorio de Referencia Nacional Virus Respiratorios, Instituto Nacional de Salud | Centers for Disease Control and Prevention |  | |
| EPI_ISL_273689 | EPI1197552 | A/H3 | Peru | 2017-Dec-07 | A/Peru/3517/2017 | Laboratorio de Referencia Nacional Virus Respiratorios, Instituto Nacional de Salud | Centers for Disease Control and Prevention |  | |
| EPI_ISL_273690 | EPI1180968 | A/H3 | Peru | 2017-Dec-07 | A/Peru/3717/2017 | Laboratorio de Referencia Nacional Virus Respiratorios, Instituto Nacional de Salud | Centers for Disease Control and Prevention |  | |
| EPI_ISL_273691 | EPI1174591 | A/H3 | Peru | 2017-Dec-07 | A/Peru/3817/2017 | Laboratorio de Referencia Nacional Virus Respiratorios, Instituto Nacional de Salud | Centers for Disease Control and Prevention |  | |
| EPI_ISL_273692 | EPI1174583 | A/H3 | Peru | 2017-Dec-15 | A/Peru/810517/2017 | Laboratorio de Referencia Nacional Virus Respiratorios, Instituto Nacional de Salud | Centers for Disease Control and Prevention |  | |
| EPI_ISL_273693 | EPI1174567 | A/H3 | Peru | 2017-Nov-28 | A/Peru/751017/2017 | Laboratorio de Referencia Nacional Virus Respiratorios, Instituto Nacional de Salud | Centers for Disease Control and Prevention |  | |
| EPI_ISL_273694 | EPI1174559 | A/H3 | Peru | 2017-Nov-16 | A/Peru/806817/2017 | Laboratorio de Referencia Nacional Virus Respiratorios, Instituto Nacional de Salud | Centers for Disease Control and Prevention |  | |
| EPI_ISL_273695 | EPI1174551 | A/H3 | Peru | 2017-Nov-09 | A/Peru/4617/2017 | Laboratorio de Referencia Nacional Virus Respiratorios, Instituto Nacional de Salud | Centers for Disease Control and Prevention |  | |
| EPI_ISL_273696 | EPI1174543 | A/H3 | Peru | 2017-Nov-08 | A/Peru/5117/2017 | Laboratorio de Referencia Nacional Virus Respiratorios, Instituto Nacional de Salud | Centers for Disease Control and Prevention |  | |
| EPI_ISL_298367 | EPI1174535 | A/H3 | Peru | 2017-Dec-15 | A/Peru/7117/2017 | Laboratorio de Referencia Nacional Virus Respiratorios, Instituto Nacional de Salud | Centers for Disease Control and Prevention |  | |
| EPI_ISL_298368 | EPI1174527 | A/H3 | Peru | 2017-Dec-07 | A/Peru/3917/2017 | Laboratorio de Referencia Nacional Virus Respiratorios, Instituto Nacional de Salud | Centers for Disease Control and Prevention |  | |
| EPI_ISL_298369 | EPI1174519 | A/H3 | Peru | 2017-Nov-16 | A/Peru/7617/2017 | Laboratorio de Referencia Nacional Virus Respiratorios, Instituto Nacional de Salud | Centers for Disease Control and Prevention |  | |
| EPI_ISL_298370 | EPI1174511 | A/H3 | Peru | 2017-Dec-04 | A/Peru/7917/2017 | Laboratorio de Referencia Nacional Virus Respiratorios, Instituto Nacional de Salud | Centers for Disease Control and Prevention |  | |
| EPI_ISL_298371 | EPI1174503 | A/H3 | Peru | 2017-Dec-01 | A/Peru/2117/2017 | Laboratorio de Referencia Nacional Virus Respiratorios, Instituto Nacional de Salud | Centers for Disease Control and Prevention |  | |
| EPI_ISL_298372 | EPI1174495 | A/H3 | Peru | 2017-Nov-10 | A/Peru/5217/2017 | Laboratorio de Referencia Nacional Virus Respiratorios, Instituto Nacional de Salud | Centers for Disease Control and Prevention |  | |
| EPI_ISL_298373 | EPI1039089 | A/H3 | Peru | 2017-Jun-14 | A/Peru/9417/2017 | Laboratorio de Referencia Nacional Virus Respiratorios, Instituto Nacional de Salud | Centers for Disease Control and Prevention |  | |
| EPI_ISL_298374 | EPI1039081 | A/H3 | Peru | 2017-Jun-11 | A/Peru/1617/2017 | Laboratorio de Referencia Nacional Virus Respiratorios, Instituto Nacional de Salud | Centers for Disease Control and Prevention |  | |
| EPI_ISL_298375 | EPI1039073 | A/H3 | Peru | 2017-Jun-12 | A/Peru/0817/2017 | Laboratorio de Referencia Nacional Virus Respiratorios, Instituto Nacional de Salud | Centers for Disease Control and Prevention |  | |
| EPI_ISL_298376 | EPI1039065 | A/H3 | Peru | 2017-Jun-12 | A/Peru/1017/2017 | Laboratorio de Referencia Nacional Virus Respiratorios, Instituto Nacional de Salud | Centers for Disease Control and Prevention |  | |
| EPI_ISL_298378 | EPI1039057 | A/H3 | Peru | 2017-Jun-07 | A/Peru/6917/2017 | Laboratorio de Referencia Nacional Virus Respiratorios, Instituto Nacional de Salud | Centers for Disease Control and Prevention |  | |
| EPI_ISL_298379 | EPI1039049 | A/H3 | Peru | 2017-Jun-11 | A/Peru/8817/2017 | Laboratorio de Referencia Nacional Virus Respiratorios, Instituto Nacional de Salud | Centers for Disease Control and Prevention |  | |
| EPI_ISL_299938 | EPI1039041 | A/H3 | Peru | 2017-Jun-07 | A/Peru/7017/2017 | Laboratorio de Referencia Nacional Virus Respiratorios, Instituto Nacional de Salud | Centers for Disease Control and Prevention |  | |
| EPI_ISL_303134 | EPI1039033 | A/H3 | Peru | 2017-Jun-06 | A/Peru/5417/2017 | Laboratorio de Referencia Nacional Virus Respiratorios, Instituto Nacional de Salud | Centers for Disease Control and Prevention |  | |
| EPI_ISL_319723 | EPI1039025 | A/H3 | Peru | 2017-Jun-06 | A/Peru/8317/2017 | Laboratorio de Referencia Nacional Virus Respiratorios, Instituto Nacional de Salud | Centers for Disease Control and Prevention |  | |
| EPI_ISL_319724 | EPI1039017 | A/H3 | Peru | 2017-May-31 | A/Peru/2217/2017 | Laboratorio de Referencia Nacional Virus Respiratorios, Instituto Nacional de Salud | Centers for Disease Control and Prevention |  | |
| EPI_ISL_319725 | EPI1039003 | A/H3 | Peru | 2017-May-19 | A/Peru/6817/2017 | Laboratorio de Referencia Nacional Virus Respiratorios, Instituto Nacional de Salud | Centers for Disease Control and Prevention |  | |
| EPI_ISL_319726 | EPI1038989 | A/H3 | Peru | 2017-May-24 | A/Peru/0517/2017 | Laboratorio de Referencia Nacional Virus Respiratorios, Instituto Nacional de Salud | Centers for Disease Control and Prevention |  | |
| EPI_ISL_319727 | EPI1038969 | A/H3 | Peru | 2017-May-17 | A/Peru/8117/2017 | Laboratorio de Referencia Nacional Virus Respiratorios, Instituto Nacional de Salud | Centers for Disease Control and Prevention |  | |
| EPI_ISL_319728 | EPI1038953 | A/H3 | Peru | 2017-May-10 | A/Peru/7217/2017 | Laboratorio de Referencia Nacional Virus Respiratorios, Instituto Nacional de Salud | Centers for Disease Control and Prevention |  | |
| EPI_ISL_319729 | EPI1038939 | A/H3 | Peru | 2017-May-09 | A/Peru/5817/2017 | Laboratorio de Referencia Nacional Virus Respiratorios, Instituto Nacional de Salud | Centers for Disease Control and Prevention |  | |
| EPI_ISL_274426 | EPI1042464 | B/Vic | Peru | 2017-Jun-07 | B/Peru/0017/2017 | Laboratorio de Referencia Nacional Virus Respiratorios, Instituto Nacional de Salud | Centers for Disease Control and Prevention |  | |
| EPI_ISL_298454 | EPI1175188 | B/Vic | Peru | 2017-Dec-07 | B/Peru/3217/2017 | Laboratorio de Referencia Nacional Virus Respiratorios, Instituto Nacional de Salud | Centers for Disease Control and Prevention |  | |
| EPI_ISL_298455 | EPI1175196 | B/Vic | Peru | 2017-Dec-07 | B/Peru/4117/2017 | Laboratorio de Referencia Nacional Virus Respiratorios, Instituto Nacional de Salud | Centers for Disease Control and Prevention |  | |
| EPI_ISL_298457 | EPI1175212 | B/Vic | Peru | 2017-Dec-07 | B/Peru/3117/2017 | Laboratorio de Referencia Nacional Virus Respiratorios, Instituto Nacional de Salud | Centers for Disease Control and Prevention |  | |
| EPI_ISL_298458 | EPI1175220 | B/Vic | Peru | 2017-Dec-07 | B/Peru/4417/2017 | Laboratorio de Referencia Nacional Virus Respiratorios, Instituto Nacional de Salud | Centers for Disease Control and Prevention |  | |
| EPI_ISL_298460 | EPI1175236 | B/Vic | Peru | 2017-Dec-13 | B/Peru/7917/2017 | Laboratorio de Referencia Nacional Virus Respiratorios, Instituto Nacional de Salud | Centers for Disease Control and Prevention |  | |
| EPI_ISL_300147 | EPI1182451 | B/Vic | Peru | 2017-Dec-07 | B/Peru/754817/2017 | Laboratorio de Referencia Nacional Virus Respiratorios, Instituto Nacional de Salud | Centers for Disease Control and Prevention |  | |
| EPI_ISL_300148 | EPI1182459 | B/Vic | Peru | 2017-Dec-07 | B/Peru/754617/2017 | Laboratorio de Referencia Nacional Virus Respiratorios, Instituto Nacional de Salud | Centers for Disease Control and Prevention |  | |
| EPI_ISL_305184 | EPI1206860 | B/Vic | Peru | 2017-Dec-12 | B/Peru/6917/2017 | Laboratorio de Referencia Nacional Virus Respiratorios, Instituto Nacional de Salud | Centers for Disease Control and Prevention |  | |
| EPI_ISL_305187 | EPI1206884 | B/Vic | Peru | 2017-Nov-07 | B/Peru/2817/2017 | Laboratorio de Referencia Nacional Virus Respiratorios, Instituto Nacional de Salud | Centers for Disease Control and Prevention |  | |
| EPI_ISL_319689 | EPI1266040 | B/Vic | Peru | 2018-May-31 | B/Peru/7218/2018 | Laboratorio de Referencia Nacional Virus Respiratorios, Instituto Nacional de Salud | Centers for Disease Control and Prevention |  | |
| EPI_ISL_298456 | EPI1175204 | B/Yam | Peru | 2017-Nov-30 | B/Peru/4517/2017 | Laboratorio de Referencia Nacional Virus Respiratorios, Instituto Nacional de Salud | Centers for Disease Control and Prevention |  | |
| EPI_ISL_298459 | EPI1175228 | B/Yam | Peru | 2017-Dec-12 | B/Peru/7117/2017 | Laboratorio de Referencia Nacional Virus Respiratorios, Instituto Nacional de Salud | Centers for Disease Control and Prevention |  | |
| EPI_ISL_298461 | EPI1175244 | B/Yam | Peru | 2017-Dec-22 | B/Peru/5117/2017 | Laboratorio de Referencia Nacional Virus Respiratorios, Instituto Nacional de Salud | Centers for Disease Control and Prevention |  | |
| EPI_ISL_305185 | EPI1206868 | B/Yam | Peru | 2017-Dec-13 | B/Peru/7417/2017 | Laboratorio de Referencia Nacional Virus Respiratorios, Instituto Nacional de Salud | Centers for Disease Control and Prevention |  | |
| EPI_ISL_305186 | EPI1206876 | B/Yam | Peru | 2017-Dec-13 | B/Peru/7517/2017 | Laboratorio de Referencia Nacional Virus Respiratorios, Instituto Nacional de Salud | Centers for Disease Control and Prevention |  | |
| EPI_ISL_319688 | EPI1266036 | B/Yam | Peru | 2018-Apr-12 | B/Peru/5318/2018 | Laboratorio de Referencia Nacional Virus Respiratorios, Instituto Nacional de Salud | Centers for Disease Control and Prevention |  | |
| EPI_ISL_319690 | EPI1266048 | B/Yam | Peru | 2018-Apr-17 | B/Peru/3718/2018 | Laboratorio de Referencia Nacional Virus Respiratorios, Instituto Nacional de Salud | Centers for Disease Control and Prevention |  | |
| EPI_ISL_319691 | EPI1266056 | B/Yam | Peru | 2018-May-22 | B/Peru/6618/2018 | Laboratorio de Referencia Nacional Virus Respiratorios, Instituto Nacional de Salud | Centers for Disease Control and Prevention |  | |
| EPI_ISL_319692 | EPI1266064 | B/Yam | Peru | 2018-May-11 | B/Peru/7818/2018 | Laboratorio de Referencia Nacional Virus Respiratorios, Instituto Nacional de Salud | Centers for Disease Control and Prevention |  | |
| EPI_ISL_319693 | EPI1266072 | B/Yam | Peru | 2018-May-05 | B/Peru/7418/2018 | Laboratorio de Referencia Nacional Virus Respiratorios, Instituto Nacional de Salud | Centers for Disease Control and Prevention |  | |
| EPI_ISL_329988 | EPI1311472 | A/H1pdm09 | Uruguay | 2018-Aug-02 | A/Uruguay/664/2018 | Departamento de Laboratorio de Salud Pública (DLSP) | National Influenza Center, Ministry of Health |  | |
| EPI_ISL_329989 | EPI1311473 | A/H1pdm09 | Uruguay | 2018-Aug-07 | A/uruguay/670/2018 | Departamento de Laboratorio de Salud Pública (DLSP) | National Influenza Center, Ministry of Health |  | |
| EPI_ISL_329990 | EPI1311474 | A/H1pdm09 | Uruguay | 2018-Aug-09 | A/Uruguay/679/2018 | Departamento de Laboratorio de Salud Pública (DLSP) | National Influenza Center, Ministry of Health |  | |
| EPI_ISL_329991 | EPI1311475 | A/H1pdm09 | Uruguay | 2018-Aug-09 | A/Uruguay/682/2018 | Departamento de Laboratorio de Salud Pública (DLSP) | National Influenza Center, Ministry of Health |  | |
| EPI_ISL_329992 | EPI1311476 | A/H1pdm09 | Uruguay | 2018-Aug-21 | A/Uruguay/840/2018 | Departamento de Laboratorio de Salud Pública (DLSP) | National Influenza Center, Ministry of Health |  | |
| EPI_ISL_329993 | EPI1311477 | A/H1pdm09 | Uruguay | 2018-Aug-22 | A/Uruguay/819/2018 | Departamento de Laboratorio de Salud Pública (DLSP) | National Influenza Center, Ministry of Health |  | |
| EPI_ISL_330180 | EPI1312486 | A/H1pdm09 | Uruguay | 2018-Jul-30 | A/Uruguay/597/2018 | Departamento de Laboratorio de Salud Pública (DLSP) | Centers for Disease Control and Prevention |  | |
| EPI_ISL_330181 | EPI1312494 | A/H1pdm09 | Uruguay | 2018-Jul-24 | A/Uruguay/577/2018 | Departamento de Laboratorio de Salud Pública (DLSP) | Centers for Disease Control and Prevention |  | |
| EPI_ISL_330182 | EPI1312502 | A/H1pdm09 | Uruguay | 2018-Jul-18 | A/Uruguay/540/2018 | Departamento de Laboratorio de Salud Pública (DLSP) | Centers for Disease Control and Prevention |  | |
| EPI_ISL_330183 | EPI1312510 | A/H1pdm09 | Uruguay | 2018-Jul-16 | A/Uruguay/516/2018 | Departamento de Laboratorio de Salud Pública (DLSP) | Centers for Disease Control and Prevention |  | |
| EPI_ISL_330184 | EPI1312518 | A/H1pdm09 | Uruguay | 2018-Jun-27 | A/Uruguay/397/2018 | Departamento de Laboratorio de Salud Pública (DLSP) | Centers for Disease Control and Prevention |  | |
| EPI_ISL_330185 | EPI1312526 | A/H1pdm09 | Uruguay | 2018-May-10 | A/Uruguay/137/2018 | Departamento de Laboratorio de Salud Pública (DLSP) | Centers for Disease Control and Prevention |  | |
| EPI_ISL_272932 | EPI1311471 | A/H3 | Uruguay | 2018-Aug-27 | A/Uruguay/870/2018 | Departamento de Laboratorio de Salud Pública (DLSP) | National Influenza Center, Ministry of Health |  | |
| EPI_ISL_272934 | EPI1311470 | A/H3 | Uruguay | 2018-Aug-27 | A/Uruguay/860/2018 | Departamento de Laboratorio de Salud Pública (DLSP) | National Influenza Center, Ministry of Health |  | |
| EPI_ISL_272935 | EPI1311469 | A/H3 | Uruguay | 2018-Aug-03 | A/Uruguay/633/2018 | Departamento de Laboratorio de Salud Pública (DLSP) | National Influenza Center, Ministry of Health |  | |
| EPI_ISL_272936 | EPI1313319 | A/H3 | Uruguay | 2018-Aug-01 | A/Uruguay/610/2018 | Departamento de Laboratorio de Salud Pública (DLSP) | Centers for Disease Control and Prevention |  | |
| EPI_ISL_272937 | EPI1313096 | A/H3 | Uruguay | 2018-Apr-02 | A/Uruguay/079/2018 | Departamento de Laboratorio de Salud Pública (DLSP) | Centers for Disease Control and Prevention |  | |
| EPI_ISL_272939 | EPI1313064 | A/H3 | Uruguay | 2018-Aug-01 | A/Uruguay/609/2018 | Departamento de Laboratorio de Salud Pública (DLSP) | Centers for Disease Control and Prevention |  | |
| EPI_ISL_275870 | EPI1048983 | A/H3 | Uruguay | 2017-Jun-22 | A/Uruguay/412/2017 | Departamento de Laboratorio de Salud Pública (DLSP) | Centers for Disease Control and Prevention |  | |
| EPI_ISL_275872 | EPI1048967 | A/H3 | Uruguay | 2017-Jun-22 | A/Uruguay/400/2017 | Departamento de Laboratorio de Salud Pública (DLSP) | Centers for Disease Control and Prevention |  | |
| EPI_ISL_275874 | EPI1048951 | A/H3 | Uruguay | 2017-Jun-15 | A/Uruguay/343/2017 | Departamento de Laboratorio de Salud Pública (DLSP) | Centers for Disease Control and Prevention |  | |
| EPI_ISL_275876 | EPI1048936 | A/H3 | Uruguay | 2017-Jul-03 | A/Uruguay/525/2017 | Departamento de Laboratorio de Salud Pública (DLSP) | Centers for Disease Control and Prevention |  | |
| EPI_ISL_275878 | EPI1048917 | A/H3 | Uruguay | 2017-Jun-15 | A/Uruguay/332/2017 | Departamento de Laboratorio de Salud Pública (DLSP) | Centers for Disease Control and Prevention |  | |
| EPI_ISL_275880 | EPI1048900 | A/H3 | Uruguay | 2017-Jun-29 | A/Uruguay/468/2017 | Departamento de Laboratorio de Salud Pública (DLSP) | Centers for Disease Control and Prevention |  | |
| EPI_ISL_275882 | EPI1048882 | A/H3 | Uruguay | 2017-Jun-21 | A/Uruguay/384/2017 | Departamento de Laboratorio de Salud Pública (DLSP) | Centers for Disease Control and Prevention |  | |
| EPI_ISL_275884 | EPI1048867 | A/H3 | Uruguay | 2017-Jun-19 | A/Uruguay/375/2017 | Departamento de Laboratorio de Salud Pública (DLSP) | Centers for Disease Control and Prevention |  | |
| EPI_ISL_329985 | EPI1035665 | A/H3 | Uruguay | 2017-May-16 | A/Uruguay/89/2017 | Departamento de Laboratorio de Salud Pública (DLSP) | Centers for Disease Control and Prevention |  | |
| EPI_ISL_329986 | EPI1035649 | A/H3 | Uruguay | 2017-May-31 | A/Uruguay/170/2017 | Departamento de Laboratorio de Salud Pública (DLSP) | Centers for Disease Control and Prevention |  | |
| EPI_ISL_329987 | EPI1035641 | A/H3 | Uruguay | 2017-Jun-02 | A/Uruguay/163/2017 | Departamento de Laboratorio de Salud Pública (DLSP) | Centers for Disease Control and Prevention |  | |
| EPI_ISL_330253 | EPI1035633 | A/H3 | Uruguay | 2017-May-30 | A/Uruguay/146/2017 | Departamento de Laboratorio de Salud Pública (DLSP) | Centers for Disease Control and Prevention |  | |
| EPI_ISL_330257 | EPI1035625 | A/H3 | Uruguay | 2017-May-31 | A/Uruguay/143/2017 | Departamento de Laboratorio de Salud Pública (DLSP) | Centers for Disease Control and Prevention |  | |
| EPI_ISL_330285 | EPI1035610 | A/H3 | Uruguay | 2017-May-18 | A/Uruguay/108/2017 | Departamento de Laboratorio de Salud Pública (DLSP) | Centers for Disease Control and Prevention |  | |
| EPI_ISL_272654 | EPI1034156 | B/Vic | Uruguay | 2017-May-30 | B/Uruguay/124/2017 | Departamento de Laboratorio de Salud Pública (DLSP) | Centers for Disease Control and Prevention |  | |
| EPI_ISL_272656 | EPI1034172 | B/Vic | Uruguay | 2017-May-31 | B/Uruguay/137/2017 | Departamento de Laboratorio de Salud Pública (DLSP) | Centers for Disease Control and Prevention |  | |
| EPI_ISL_272657 | EPI1034180 | B/Vic | Uruguay | 2017-May-31 | B/Uruguay/138/2017 | Departamento de Laboratorio de Salud Pública (DLSP) | Centers for Disease Control and Prevention |  | |
| EPI_ISL_272658 | EPI1034188 | B/Vic | Uruguay | 2017-May-31 | B/Uruguay/140/2017 | Departamento de Laboratorio de Salud Pública (DLSP) | Centers for Disease Control and Prevention |  | |
| EPI_ISL_276585 | EPI1053015 | B/Vic | Uruguay | 2017-Jun-30 | B/Uruguay/511/2017 | Departamento de Laboratorio de Salud Pública (DLSP) | Centers for Disease Control and Prevention |  | |
| EPI_ISL_276586 | EPI1053023 | B/Vic | Uruguay | 2017-Jun-19 | B/Uruguay/379/2017 | Departamento de Laboratorio de Salud Pública (DLSP) | Centers for Disease Control and Prevention |  | |
| EPI_ISL_276590 | EPI1053055 | B/Vic | Uruguay | 2017-Jun-20 | B/Uruguay/383/2017 | Departamento de Laboratorio de Salud Pública (DLSP) | Centers for Disease Control and Prevention |  | |
| EPI_ISL_272651 | EPI1311466 | B/Yam | Uruguay | 2018-Aug-01 | B/Uruguay/626/2018 | Departamento de Laboratorio de Salud Pública (DLSP) | National Influenza Center, Ministry of Health |  | |
| EPI_ISL_272652 | EPI1311467 | B/Yam | Uruguay | 2018-Aug-23 | B/Uruguay/835/2018 | Departamento de Laboratorio de Salud Pública (DLSP) | National Influenza Center, Ministry of Health |  | |
| EPI_ISL_272653 | EPI1311468 | B/Yam | Uruguay | 2018-Aug-29 | B/Uruguay/899/2018 | Departamento de Laboratorio de Salud Pública (DLSP) | National Influenza Center, Ministry of Health |  | |
| EPI_ISL_272655 | EPI1034133 | B/Yam | Uruguay | 2017-May-23 | B/Uruguay/106/2017 | Departamento de Laboratorio de Salud Pública (DLSP) | Centers for Disease Control and Prevention |  | |
| EPI_ISL_276587 | EPI1034141 | B/Yam | Uruguay | 2017-May-25 | B/Uruguay/113/2017 | Departamento de Laboratorio de Salud Pública (DLSP) | Centers for Disease Control and Prevention |  | |
| EPI_ISL_276588 | EPI1034148 | B/Yam | Uruguay | 2017-May-26 | B/Uruguay/119/2017 | Departamento de Laboratorio de Salud Pública (DLSP) | Centers for Disease Control and Prevention |  | |
| EPI_ISL_276589 | EPI1034164 | B/Yam | Uruguay | 2017-May-29 | B/Uruguay/126/2017 | Departamento de Laboratorio de Salud Pública (DLSP) | Centers for Disease Control and Prevention |  | |
| EPI_ISL_329982 | EPI1053031 | B/Yam | Uruguay | 2017-Jun-20 | B/Uruguay/403/2017 | Departamento de Laboratorio de Salud Pública (DLSP) | Centers for Disease Control and Prevention |  | |
| EPI_ISL_329983 | EPI1053039 | B/Yam | Uruguay | 2017-Jun-30 | B/Uruguay/527/2017 | Departamento de Laboratorio de Salud Pública (DLSP) | Centers for Disease Control and Prevention |  | |
| EPI_ISL_329984 | EPI1053047 | B/Yam | Uruguay | 2017-Jun-15 | B/Uruguay/338/2017 | Departamento de Laboratorio de Salud Pública (DLSP) | Centers for Disease Control and Prevention |  | |
| EPI_ISL_330219 | EPI1312796 | B/Yam | Uruguay | 2018-Jul-24 | B/Uruguay/571/2018 | Departamento de Laboratorio de Salud Pública (DLSP) | Centers for Disease Control and Prevention |  | |
| EPI_ISL_330220 | EPI1312804 | B/Yam | Uruguay | 2018-Jul-19 | B/Uruguay/553/2018 | Departamento de Laboratorio de Salud Pública (DLSP) | Centers for Disease Control and Prevention |  | |
| EPI_ISL_330221 | EPI1312812 | B/Yam | Uruguay | 2018-Feb-20 | B/Uruguay/033/2018 | Departamento de Laboratorio de Salud Pública (DLSP) | Centers for Disease Control and Prevention |  | |
