# Supplementary material for: Genetic evolution of influenza viruses among selected countries in Latin America, 2017–2018
Source: PLoS One. 2020 Mar 10;15(3):e0227962. doi: 10.1371/journal.pone.0227962 (PMC7064222; doi:10.1371/journal.pone.0227962)
Supplement: Attachment — Submitted filename: PLoSOne-Leite-2019-KX.pdf [file pone.0227962.s002.pdf]

Review for *PLoS One* manuscript "Genetic evolution of influenza viruses among selected countries in Latin America, 2017-2018."

Overview

The manuscript analyzes influenza virus evolution in Latin America using sequence data in the GISAID database. The authors identify a set of hemagglutinin sequences representing influenza A/H1pdm09, A/H3, and B generated from Latin American sequencing centers and from the US WHO Collaborating Center based on samples collected at Latin American sentinel sites from 2017 to 2018. They perform a phylogenetic analysis of these sequences, identify the substitutions that occur along the phylogeny, and calculate the frequencies of viral clades. They find that sequences from Latin America are broadly aligned with clade frequencies worldwide.

The manuscript addresses the important topic of influenza's genetic diversity in an under-surveilled part of the world. The analyses are appropriately conducted according to standard methods, although the phylogenetic methods themselves are not especially novel. I find that the manuscript could be strengthened by providing more precision about major conclusions regarding the evolution of viruses in Latin American and their clade frequencies relative to other areas of the world. Much of this work can be done through rewriting and clarifying the discussion, but some simple additional analyses would substantially strengthen the manuscript as well.

Major comments

1. The authors state in the discussion that viruses identified in Latin America resemble the viruses that are detected globally (lines 353-355). Although the authors compare the genetic groups that they identify with the vaccine strains selected in different years, they do not provide sufficient analyses to show to what extent the viruses sequenced in Latin America resemble global genetic diversity. To address this important and central question of geographic distributions, it would be helpful to do some of the following, in declining order of difficulty and importance:
    a. Provide a plot of global clade frequencies to parallel the plots of clade frequencies in Latin America in Figure 6. Generating these clade frequencies directly from global sequence data may be laborious, but the authors may be able to reproduce figures from other papers or to discuss clade frequencies in Latin America in comparison to those reported globally on sites like nextstrain.org.
    b. Provide a global phylogeny of influenza that includes some sequences from Latin America (subsampling the dataset would likely be necessary to conduct a more easily interpretable analysis), and label the sequences from Latin America. If these sequences are dispersed through the global tree, then this finding would strengthen the authors' argument that viruses in Latin America resemble global genetic diversity.
    c. Various other studies have addressed the geographic distribution of influenza, though not necessarily with respect to Latin America in particular (one example: https://www.ncbi.nlm.nih.gov/pubmed/26053121). The authors should cite more of this relevant literature to provide additional context for their conclusions.
2. The authors write in the discussion that "the viruses that circulated in these countries during

the early part of the 2017 Southern Hemisphere influenza season evolved and changed as compared to those that circulated at the end of the 2018 Southern Hemisphere season" (lines 350-353). This statement is imprecise and could benefit from additional clarification. Evolution could refer to the accumulation of neutral mutations, antigenic drift, competition between clades carrying distinct antigenic mutations, and many other phenomena. In the rest of their study, the authors already identified some of the specific molecular changes that occurred, and while their analyses are not powered to identify the particular evolutionary forces at work, they could make this part of the discussion more detailed and precise.

Minor comments

1. The authors should provide the standard acknowledgements table required for use of GISAID sequences.

2. Axis labels on Figure 1 are upside down.

3. Sequence names in Figures 2-5 are mostly illegible, and the authors might consider replacing each sequence name with a colored dot representing time of collection instead.

4. lines 202-212: The number of HA sequences included in the final analysis (1395) is less than the numbers produced by the participating NICs and the number uploaded by the WHO CC at the US CDC (761 + 1169). Please clarify the reason for the discrepancy. Were the excluded sequences ones that had been passaged, or duplicates of other sequences, or was there some other reason?

5. When defining clades and subclades, as in lines 227 and 232-233, for example, please clarify whether and when the clades being defined are part of a standard nomenclature.

6. In lines 365, 375, 382, 391, and 410, the authors write that about the "reduction" of a virus with ferret anti-sera raised against particular viral strains. Perhaps the authors should consider using the more common and clearer term "inhibition" or "hemagglutination inhibition" instead.
