# Supplementary material for: Genetic evolution of influenza viruses among selected countries in Latin America, 2017–2018
Source: PLoS One. 2020 Mar 10;15(3):e0227962. doi: 10.1371/journal.pone.0227962 (PMC7064222; doi:10.1371/journal.pone.0227962)
Supplement: Attachment — Submitted filename: Palekar_GSD_AmericasPlos revisions_responses.docx [file pone.0227962.s003.docx]

Dear Editors,

Thank you for your thoughtful review of our manuscript. Please find the responses to your comments below.

Sincerely,

Rakhee Palekar

**General comments**

Comment 1: The manuscript has to undergo language editing.

Response 1: This was done by a native English speaker, who is an influenza expert.

Comment 2: Samples were collected during a limited time period, please add a Discussion point as to how this could have affected the results.

Response 2: We have added a sentence in the discussion.

Comment 3: In order to properly interpret the data from this manuscript, a comparison with global genetic diversity of the virus would be necessary.

**Journal requirements**

Comment. 4: Please ensure that your manuscript meets PLOS ONE's style requirements, including those for file naming.

Response 4: We have done this.

Comment 5: Please change Fig.1, so that the names of the axes are clearly legible.

Response 5: We have updated the figure.

**Reviewer specific comments**

Reviewer #1:

Comment 5: On the other hand, the quantity and analysis period of clinical samples and HA sequences of influenza virus is still limited, which influence the reliability of the study to some extent.

Response: Thank you for the comment.

Reviewer #2

Comment 6: In line 100 reference style is not correct. Please make sure all the references are correct and in appropriate order and style.

Response #6: The reference was corrected.

Comment 7: Also make the sample numbers very clear it make the reader to confuse about the sample numbers for each specific strain of Influenza virus.

Comment 7: We have done this.

Reviewer #3

Comment 8: The authors state in the discussion that viruses identified in Latin America resemble the viruses that are detected globally (lines 353-355). Although the authors compare the genetic groups that they identify with the vaccine strains selected in different years, they do not provide sufficient analyses to show to what extent the viruses sequenced in Latin America resemble global genetic diversity. To address this important and central question of geographic distributions, it would be helpful to do some of the following, in declining order of difficulty and importance:

1. Provide a plot of global clade frequencies to parallel the plots of clade frequencies in Latin America in Figure 6. Generating these clade frequencies directly from global sequence data may be laborious, but the authors may be able to reproduce figures from other papers or to discuss clade frequencies in Latin America in comparison to those reported globally on sites like nextstrain.org.

Response: We have added Figure 7 showing the global clades frequencies. The global findings align with the study results.

2. Provide a global phylogeny of influenza that includes some sequences from Latin America (subsampling the dataset would likely be necessary to conduct a more easily interpretable analysis), and label the sequences from Latin America. If these sequences are dispersed through the global tree, then this finding would strengthen the authors' argument that viruses in Latin America resemble global genetic diversity.

Response: Based on the global distribution of the phylogenetic groups and clades, the phylogenetic inferences obtained for Latin America resembles the global genetic diversity. Since the main focus of the study was the Latin America countries, new phylogenetics trees were not added to avoid overloading the paper.

3. Various other studies have addressed the geographic distribution of influenza, though not necessarily with respect to Latin America in particular (one example: https://www.ncbi.nlm.nih.gov/pubmed/26053121). The authors should cite more of this relevant literature to provide additional context for their conclusions.

Response: We have added additional relevant literature and more points to the discussion and conclusions.

Comment 9: The authors write in the discussion that "the viruses that circulated in these countries during the early part of the 2017 Southern Hemisphere influenza season evolved and changed as compared to those that circulated at the end of the 2018 Southern Hemisphere season" (lines 350-353). This statement is imprecise and could benefit from additional clarification. Evolution could refer to the accumulation of neutral mutations, antigenic drift, competition between clades carrying distinct antigenic mutations, and many other phenomena. In the rest of their study, the authors already identified some of the specific molecular changes that occurred, and while their analyses are not powered to identify the particular evolutionary forces at work, they could make this part of the discussion more detailed and precise.

Response 9: We have updated this language.

Comment 10: The authors should provide the standard acknowledgements table required for use of GISAID sequences.

Response 10: A new supplemental table was provided in the GISAID standard acknowledgement table format.

Comment 11: axis labels on Figure 1 are upside down.

Response 11: The axis labels were fixed.

Comment 12: Sequence names in Figures 2-5 are mostly illegible, and the authors might consider replacing each sequence name with a colored dot representing time of collection instead.

Comment 12: The sequences names were maintained in order to show the country of origin of the sequences.

Comment 13: lines 202-212: The number of HA sequences included in the final analysis (1395) is less than the numbers produced by the participating NICs and the number uploaded by the WHO CC at the US CDC (761 + 1169). Please clarify the reason for the discrepancy. Were the excluded sequences ones that had been passaged, or duplicates of other sequences, or was there some other reason?

Response 13: Only sequences obtained from original clinical samples were included in the analysis; sequences obtained from multiple virus-passages, incomplete HA sequences, or HA sequences with mismatches/gaps were excluded (lines 187 to 190). This explains the “discrepancy.” We added a line in the Results to clarify this.

Comment 14: When defining clades and subclades, as in lines 227 and 232-233, for example, please clarify whether and when the clades being defined are part of a standard nomenclature.

Response 14: We have reviewed this.

Comment 15: in lines 365, 375, 382, 391, and 410, the authors write that about the "reduction" of a virus with ferret anti-sera raised against particular viral strains. Perhaps the authors should consider using the more common and clearer term "inhibition" or "hemagglutination inhibition" instead.

Response 15: We have made this change in the Discussion.
