# Supplementary material for: Genetic evolution of influenza viruses among selected countries in Latin America, 2017–2018
Source: PLoS One. 2020 Mar 10;15(3):e0227962. doi: 10.1371/journal.pone.0227962 (PMC7064222; doi:10.1371/journal.pone.0227962)
Supplement: Attachment — Submitted filename: PLoSOne-Leite-2019-KX-resubmission.docx [file pone.0227962.s004.docx]

**Review for *PLoS One* manuscript “Genetic evolution of influenza viruses among selected countries in Latin America, 2017-2018.”**

The manuscript analyzes influenza virus evolution in Latin America using next-generation sequencing data. In particular, the authors study the extent to which viral genetic diversity in Latin American countries reflects broader, global patterns of genetic diversity. Since the previously submitted version of the manuscript, which I reviewed, the authors have improved the comparison of influenza clade frequencies in Latin America compared to the rest of the world by including a new Figure 7, generated from Nextstrain. I am generally satisfied that the authors have addressed my concerns about presenting a more explicit comparison of regional and global genetic diversity, but the analysis in Figure 7 contains a few remaining oddities that the authors need to correct.

Figure 7 shows global clade frequencies for the four influenza subtypes under study and is derived from the visualization interface at nextstrain.org. It is not clear to me why the y-axes of these plots end at 12% (they should run from 0-100%), or why the diversity of viruses appears to decline after about mid-2018. Both anomalies should be corrected by the authors. For example, the nextstrain.org interface shows that for B/Yamagata, the 172Q clade makes up nearly all viral diversity following 2017, but in Figure 7, the clade appears to decline in frequency until it makes up none of the viral diversity under analysis. It seems likely that the authors set a cutoff date that causes the odd appearance of the graph, but this visualization choice is confusing. It would be better for the authors to present all clade frequencies between 2017 and the present.
