# Supplementary material for: Genetic evolution of influenza viruses among selected countries in Latin America, 2017–2018
Source: PLoS One. 2020 Mar 10;15(3):e0227962. doi: 10.1371/journal.pone.0227962 (PMC7064222; doi:10.1371/journal.pone.0227962)
Supplement: Attachment — Submitted filename: Reviewer#3_response.docx [file pone.0227962.s005.docx]

Reviewer #3 Comment:

Figure 7 shows global clade frequencies for the four influenza subtypes under study and is derived from the visualization interface at [nextstrain.org](http://nextstrain.org). It is not clear to me why the y-axes of these plots end at 12% (they should run from 0-100%), or why the diversity of viruses appears to decline after about mid-2018. Both anomalies should be corrected by the authors. For example, the [nextstrain.org](http://nextstrain.org) interface shows that for B/Yamagata, the 172Q clade makes up nearly all viral diversity following 2017, but in Figure 7, the clade appears to decline in frequency until it makes up none of the viral diversity under analysis. It seems likely that the authors set a cutoff date that causes the odd appearance of the graph, but this visualization choice is confusing. It would be better for the authors to present all clade frequencies between 2017 and the present.

Response: We have edited the figure as per the request.
